# New melphalan derivatives for the treatment of retinoblastoma in combination with thermotherapy†

**DOI:** 10.1039/d4md00211c

**Published:** 2024-05-21

**Authors:** Soumaila Zebret, Mouna Hadiji, Jan Romano-deGea, Aurélien Bornet, Daniel Ortiz, Farzaneh Fadaei-Tirani, Christina Stathopoulos, Patrycja Nowak-Sliwinska, Francis L. Munier, Paul J. Dyson

**Affiliations:** a Institute of Chemical Sciences and Engineering (ISIC), École Polytechnique Fédérale de Lausanne (EPFL) 1015 Lausanne Switzerland paul.dyson@epfl.ch; b Jules-Gonin Eye Hospital, Fondation Asile des Aveugles, University of Lausanne 1004 Lausanne Switzerland francis.munier@fa2.ch; c School of Pharmaceutical Sciences, University of Geneva 1211 Geneva Switzerland; d Institute of Pharmaceutical Sciences of Western Switzerland, University of Geneva 1211 Geneva Switzerland

## Abstract

Of the different modalities used to treat retinoblastoma, a chemothermotherapeutic regimen combining carboplatin and thermotherapy (also termed focal therapy), and the application of melphalan as a monotherapy, are particularly successful. Some studies indicate that melphalan shows potential when applied in combination with focal therapy, and yet is not applied in this combination. Here we describe a series of synthetically modified melphalan derivatives that display enhanced cytotoxicity relative to melphalan itself, with some displaying further enhancements in cytotoxicity when applied in combination with heat (used as a model for thermotherapy). The synthetic approach, which involves modifying melphalan with perfluorous chains of varying lengths *via* an ester linker, could lead to a more effective treatment option for retinoblastoma with reduced side-effects, which is a key limitation of melphalan.

## Abstract 2

Melphalan, a drug in retinoblastoma treatment, was not designed for combination with focal therapy, but is more active when combined with heat. Incorporating perfluorous chains to the drug rsulted in thermoresponsive and increased cytotoxicity.

## Graphical abstract

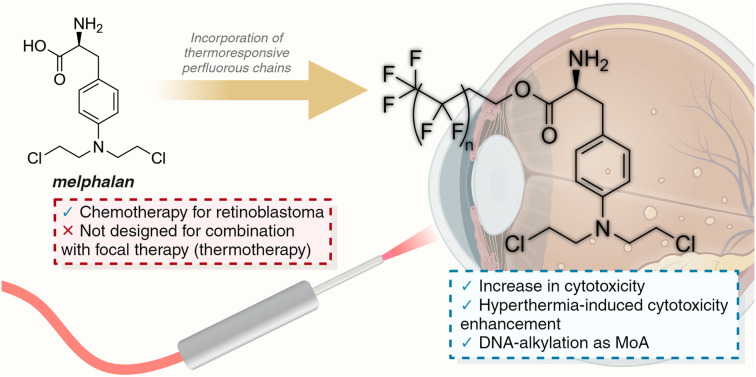

## Introduction

Chemotherapy is extensively used as an anticancer treatment modality, although the associated side-effects due to a lack of selectivity hinder efficacy,^1–7^ which in certain cases can be reduced by employing combination strategies. In this respect, strategically combining different modalities with chemotherapy is an attractive option to enhance overall efficacy of the treatment.^8,9^ One such approach is thermotherapy, which sensitises tumours towards chemotherapy,^10^ and if applied solely on the tumour, improves drug selectivity and potentially reduces side-effects.^11^ The combination of chemotherapy (principally carboplatin) and thermotherapy (application of a near-infrared laser directly onto the tumour, also referred to as focal therapy) has emerged as an advantageous strategy that overcomes certain obstacles in retinoblastoma treatment.^12–21^ The ability of thermotherapy to selectively enhance the potency of carboplatin in the heated area creates a targeted cytotoxicity to reduce the main limitations of the treatment of this rare paediatric malignancy.^12,22–26^

Melphalan is a well-established chemotherapeutic for retinoblastoma treatment^22,27–29^ and a number of studies indicate that it could be more effective when the tumour is heated.^18,22,23,30–33^ Nonetheless, melphalan is not routinely used in combination with focal therapy in the clinic and the high toxicity and fast hydrolytic deactivation of melphalan^34^ limit its use in intravenous chemotherapy for children, and consequently, it is often substituted in the clinic by other less toxic drugs that are also less efficient.^27,33,35^ Since melphalan was not developed for combination with focal therapy, it would be advantageous to modify its structure so that it synergises with heat to reduce systemic toxicity. Several thermoresponsive drugs incorporating alkyl and perfluorous chains have been previously developed.^36^ Particularly, chlorambucil, a structurally related drug to melphalan, was covalently modified with perfluorous chains to create compounds that are selectively activated in response to mild hyperthermia.^37,38^ Following a similar strategy, we modified melphalan with perfluorous chains *via* an ester linker in order to confer the resulting compounds with enhanced cytotoxicity and a degree of thermoresponsive behaviour. The synthesis and characterization of these new melphalan derivatives and a preliminary evaluation of the cytotoxicity to retinoblastoma cells are reported herein.

## Results and discussion

**Scheme 1:**
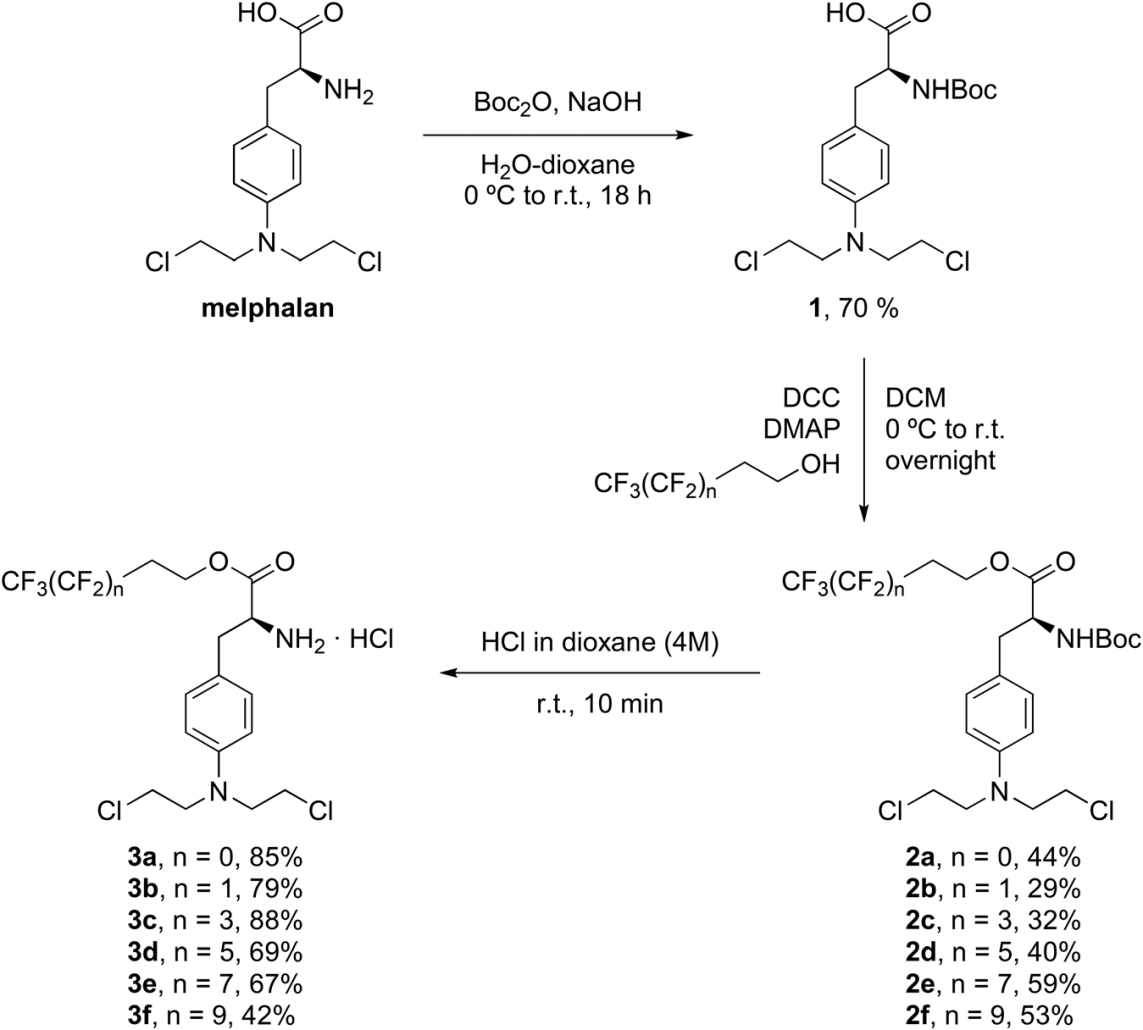
Synthesis of the perfluorous chain-modified melphalan derivatives 3a–3f.

Perfluorous chain-modified melphalan derivatives, 3a–3f, were synthesised using the three step synthetic procedure shown in Scheme 1. First, due to the sensitivity of the amino group in melphalan and the possibility of amide coupling side-reactions, protection with a *tert*-butoxycarbonyl group was performed using a literature protocol to afford 1 in 70% yield.^39^ Second, the perfluorous chains were coupled to melphalan using DCC/DMAP-mediated esterification employing the appropriate perfluorous alcohols,^38^ affording 2a–2f in moderate yields (29–59%). Third, the amino group was deprotected using HCl in dioxane^39^ to afford the desired products 3a–3f as hydrochloride salts in moderate yields (42–88%).

**Fig. 1:**
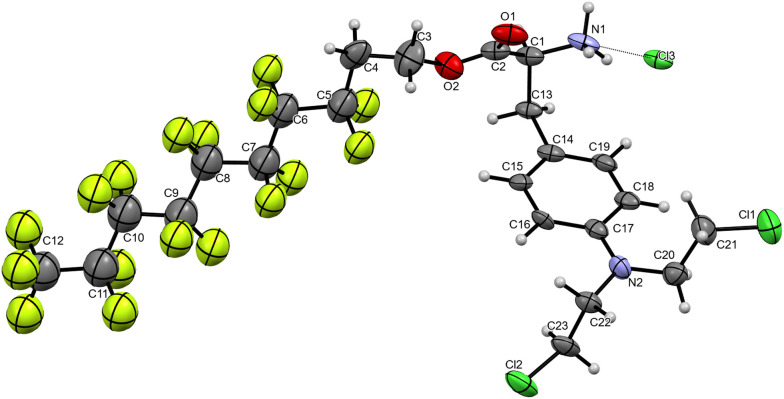
X-Ray single crystal structure of 3e. Thermal ellipsoids are drawn with 50% probability. Solvate molecules have been removed for clarity. Selected bond lengths (Å): Cl1–C21, 1.76(2); Cl2–C23, 1.761(19); N2–C17, 1.40(2); N1–C1, 1.49(2); O1–C2, 1.21(2); O2–C2, 1.29(2); C–F_avg_, 1.339(12).

All compounds were fully characterised by NMR spectroscopy and mass spectrometry. The peaks were assigned using ^19^F–^19^F COSY and ^19^F–^13^C HSQC NMR spectroscopy. No major differences were observed between the NMR spectra of melphalan, amino-protected and deprotected perfluorous chain modified melphalan, which highlights the minimal impact of the incorporation of the strongly electron-withdrawing perfluorous chain on the melphalan core due to the presence of the insulating ethylene linker (Fig. S1 and S2†). In addition, single crystals of 2d–2f and 3e were grown, with the resulting X-ray diffraction structures, corroborating the identity of the compounds. The compounds crystallised in chiral crystallographic space groups as the (*S*)-enantiomer, confirming the obtention of the desired stereoisomer. The structure of 3e is shown in Fig. 1 and the structures of intermediates 2d–2f are provided in Fig. S3 and Table S1 of the ESI.† As expected, 3e was crystallised as the hydrochloride salt and the structure contains a CF_3_(CF_2_)_7_CH_2_CH_2_ chain covalently linked to melphalan *via* an ester group.

### Biological evaluation of 3a–3f

The cytotoxicity of melphalan and the melphalan derivatives 3a–3f was evaluated on the Y79 human retinoblastoma cell line in 2D settings using an incubation time of 24 hours at a temperature of 37 °C and 5% CO_2_, or using conditions mimicking mild hyperthermia, *i.e.* incubation at 42 °C for 1 hour followed by 23 hours at 37 °C, respectively. The IC_50_ values were determined using the presto blue cell viability assay (Table 1).

**Table 1:** IC_50_ values of melphalan and derivatives 3a–3f on the human retinoblastoma (Y79) cell line after 24 hours at 37 °C or 1 hour at 42 °C followed by 23 hours at 37 °C and calculated *n*-octanol/water partition coefficients

Compound	IC_50_ (μM)		log *P*_ow_^a^
	37 °C	42 °C	
Melphalan	59 ± 5	26 ± 2	0.89
3a	1.2 ± 0.1	1.1 ± 0.1	2.96
3b	2.2 ± 0.1	1.8 ± 0.1	3.72
3c	3.4 ± 0.2	2.1 ± 0.2	5.13
3d	5.9 ± 0.3	3.3 ± 0.2	6.60
3e	13 ± 0.4	6.6 ± 0.4	8.15
3f	>200	183 ± 20	9.40

a The *n*-octanol/water partition coefficients were calculated using the SwissADME tool.^40^

Compounds 3b–3f are considerably more cytotoxic than melphalan and some also exhibit a hyperthermia-induced cytotoxicity enhancement. Such an increase in cytotoxicity, possibly a consequence of the increase in lipophilicity, could allow the administration of much lower doses in a clinical setting. An increase in cytotoxicity has also been observed in other melphalan derivatives that were modified at the carboxyl position with methyl and ethyl ester derivatives of melphalan having IC_50_ values of 1.1 ± 0.3 and 1.2 ± 0.3 μM, respectively, on the myeloma RPMI 8226 cell line, representing an 8-fold increase compared to melphalan (8.9 ± 0.3 μM).^41^ In comparison, the melphalan derivatives developed in this study present up to 50-times lower IC_50_ values than the parent drug. Compounds 3d and 3e present optimal cytotoxic behaviour, *i.e.* being approximately twice as cytotoxic at the elevated temperature, similar to the effect observed for melphalan, but with approximately 5 to 10-fold lower IC_50_ values compared to the parent drug. With shorter perfluorous chains, the thermoresponsive behaviour is not observed, although the compounds are the most cytotoxic of the series. Indeed, the cytotoxicity decreases as the length of the perfluorous chain increases (see Table 1 and Fig. S4†), with 3f, the compound with the longest perfluorous chain, being considerably less cytotoxic than melphalan. This gradual decrease in cytotoxicity correlates with the increase of the lipophilicity of the compounds. Note that the relationship between the cytotoxicity of the compounds, their thermoresponsive behaviour and the optimal length of the incorporated perfluorous chain is difficult to predict. The cytotoxicity and the hyperthermia-induced toxicity increase behaviour has been reported to increase with the length of the chain in perfluorinated derivatives of chlorambucil or ruthenium arene complexes,^38,42^ whereas it was shown to decrease in platinum(iv) carboplatin prodrugs with perfluorinated axial ligands.^43^

The cytotoxicity of the two most promising derivatives, *i.e.*3d and 3e, was subsequently evaluated on human retinoblastoma (Y79) and healthy human retina (RPE1) immortalised cells after 72 hours of incubation in 2D settings under standard conditions, *i.e.* at 37 °C and 5% CO_2_, and also under conditions that mimic mild hyperthermia, *i.e.* at 42 °C for 1 hour followed by 71 hours at 37 °C, respectively, see Table 2.

**Table 2:** IC_50_ values of 3d and 3e for human retinoblastoma cells (Y79) and healthy retina immortalised cells (RPE1) after incubating for 72 hours at 37 °C and using mild hyperthermia conditions: 1 hour at 42 °C followed by 71 hours at 37 °C

Compound	Y79		RPE1	
	IC_50_ (μM)		IC_50_ (μM)	
	37 °C	42 °C	37 °C	42 °C
3d	2.3 ± 0.1	2.1 ± 0.2	39.3 ± 5.0	31.5 ± 2.7
3e	4.5 ± 0.1	4.4 ± 0.3	52.8 ± 9.3	42.3 ± 6.0

Compounds 3d and 3e are more than an order of magnitude less cytotoxic to the healthy RPE1 cells than the tumoral Y79 cells, highlighting the remarkable selectivity of these compounds towards the retinoblastoma cells. Notably, the application of heat has comparatively little impact on the cytotoxicity towards the Y79 and the RPE1 cell lines at 72 hours, which is not unexpected given the long incubation period. In a clinical setting, the higher cytotoxicity of 3d and 3e could be advantageous, especially given their selectivity towards cancer cells. Moreover, based on the short heating times applied in the clinic, typically for 20 minutes approximately 30 minutes after injection of a drug,^44^3d and 3e are expected to show increased efficacy at elevated temperatures based on the data shown in Table 1.

### Mechanism of action

**Fig. 2:**
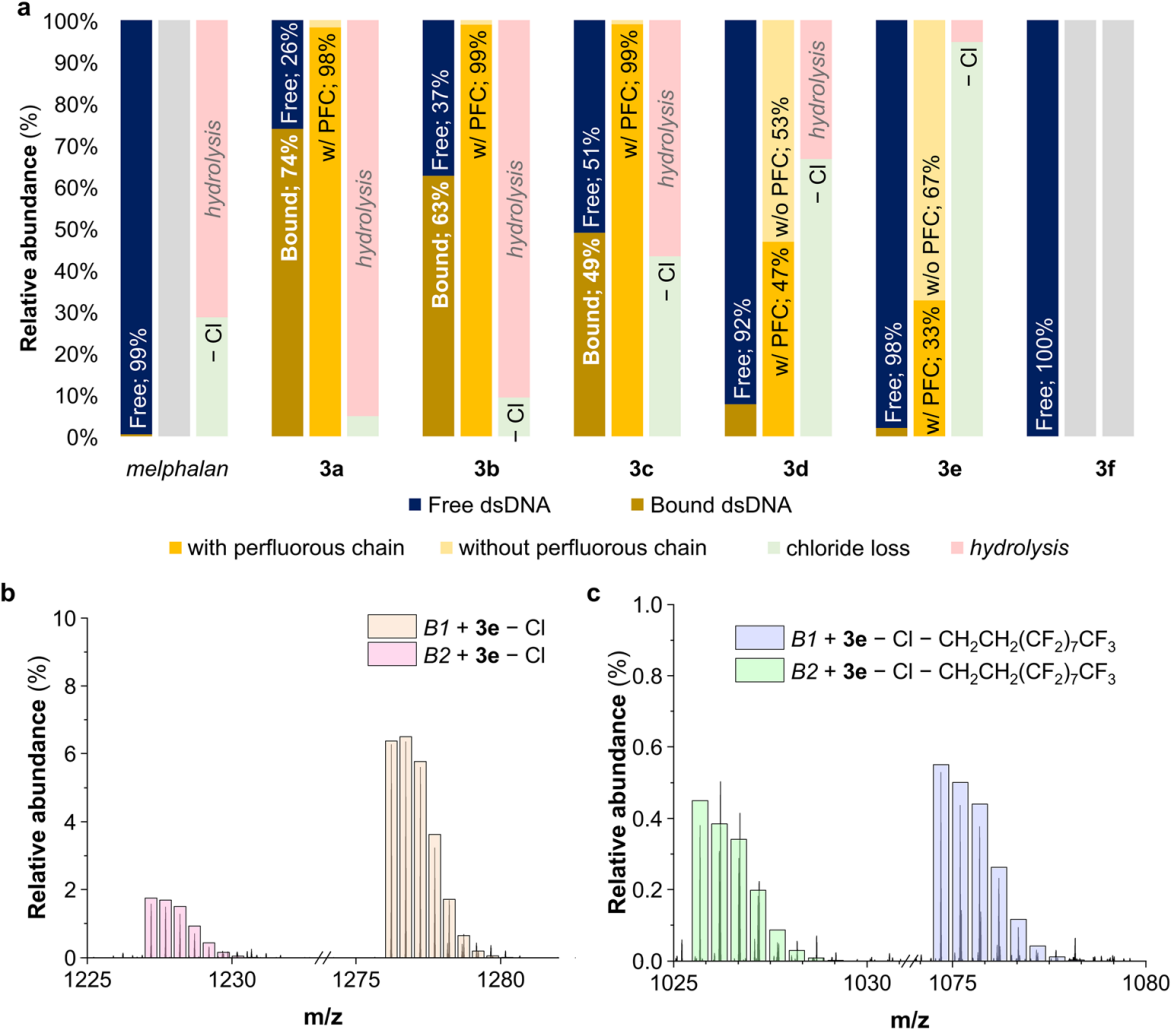
Summary of the identified ions (indicating the relative abundance of free and bound oligonucleotide, the extent of ester cleavage and the type of adducts of the bound oligonucleotide ions) by ESI-MS, a. ESI-MS spectra of the adducts of 3e with the dsDNA oligonucleotide involving the loss of chloride, b, and both the loss of chloride and the hydrolysis of the ester and subsequent loss of the perfluorous chain, c.

Melphalan is a DNA alkylating agent, and it is envisaged that derivatives 3a–3f operate *via* the same mechanism, although an additional hydrolysis of the ester linker is also expected. Hence, a DNA binding study was performed using a model double stranded oligonucleotide (dsDNA), *i.e.* 5′-AGGCAG-3′ (B1) and the complimentary strand 3′-TCCGTC-5′ (B2). Melphalan and derivatives 3a–3f were incubated with the oligonucleotide sequence at 37 °C in a 1 : 5 ratio and were sampled after incubation for 48 hours. The resulting solutions were measured by electrospray ionization mass spectrometry (ESI-MS) and the analysis of the spectra^45^ is provided in the ESI† and summarised in Fig. 2a.

Melphalan formed adducts with both strands of the oligonucleotides involving the loss of a chloride (B1/B2 + melphalan − Cl, *m*/*z* = 1075.22 and *m*/*z* = 1026.21, respectively, see Table S3 and Fig. S7–S9†), coherent with melphalan's DNA alkylation mechanism.^46^ Related adducts between the oligonucleotides and the melphalan derivatives were observed for 3a–3e, whereas no adducts were observed for 3f (*n* = 9), which correlates with the lack of cytotoxicity of this compound. Furthermore, the relative amount of melphalan derivative-bound dsDNA follows the same trend as the toxicity of the compounds. For 3a–3e, adducts involving the loss of the chloride were observed (B1/B2 + 3a–3e − Cl, see Fig. 2b and S10–S25†), indicating that the perfluorous chain melphalan derivatives have a similar mechanism of action to that of the parent drug. Additionally, significant adducts involving the alkylation of DNA and the hydrolysis of the second ethyl chloride chain were observed for 3a–3c (B1/B2 + 3a–3c − 2Cl + OH, see Fig. S10–S21†).^34^ The relative amount of these adducts decreases with the chain length, which could be a consequence of the higher hydrophobicity of the compounds and hint at a higher resistance to hydrolysis. The extent of ester cleavage appears to be more significant in 3d and 3e, the compounds with the higher hyperthermia-induced cytotoxicity enhancement. No crosslinked DNA adducts, known to be relevant hallmarks in the melphalan mechanism of action, were detected, probably due to the difficulty to observe them upon ionization.^47^ The ESI-MS spectra of 3c–3e incubated with the dsDNA also revealed the formation of adducts with oligonucleotides where the ester link is hydrolysed and the perfluorinated chain is released under the experimental conditions (B1/B2 + 3c–3e − Cl − CH_2_CH_2_(CF_2_)_*n*_CF_3_, see Fig. 2c and S26†), resulting in the same adducts observed for melphalan.

## Conclusions

We described the synthesis and enhanced cytotoxic behaviour of perfluorous chain-modified melphalan derivatives 3a–3f that have been developed for the treatment of retinoblastoma, potentially in combination with thermotherapy. Two highly promising drug candidates, 3d and 3e, were identified, displaying a 10- and 5-fold increase in cytotoxicity respectively compared to melphalan on the retinoblastoma cell line (Y79) at 37 °C and additional showing favourable properties following mild hyperthermia (42 °C). Compounds 3d and 3e also display excellent selectivity towards retinoblastoma cells relative to healthy retina cells. Based on binding studies to a dsDNA oligonucleotide, 3a–3d form the same type of adducts as melphalan both before and after the hydrolysis of the ester linkage, confirming DNA alkylation as the mechanism of action.

## Conflicts of interest

There are no conflicts to declare.

## Supplementary Material

MD-015-D4MD00211C-s001

MD-015-D4MD00211C-s002
