# Supplementary material for: New melphalan derivatives for the treatment of retinoblastoma in combination with thermotherapy
Source: RSC Med Chem. 2024 May 21;15(7):2300–4. doi: 10.1039/d4md00211c (PMC11253858; doi:10.1039/d4md00211c)
Supplement: MD-015-D4MD00211C-s001 [file MD-015-D4MD00211C-s001.pdf]

Electronic Supplementary Material (ESI) for RSC Medicinal Chemistry.
This journal is © The Royal Society of Chemistry 2024

# Supplementary information

## New melphalan derivatives for the treatment of retinoblastoma in combination with thermotherapy

Soumaila Zebret[a], Mouna Hadiji[a], Jan Romano-deGea[a], Aurélien Bornet[a], Daniel Ortiz[a], Farzaneh Fadaei-Tirani[a], Christina Stathopoulos[b], Patrycja Nowak-Sliwinska[c,d], Francis L. Munier[b,*], Paul J. Dyson[a,*]

[a] *Institute of Chemical Sciences and Engineering (ISIC), École Polytechnique Fédérale de Lausanne (EPFL), 1015 Lausanne, Switzerland*

[b] *Jules-Gonin Eye Hospital, Fondation Asile des Aveugles, University of Lausanne, 1004 Lausanne, Switzerland*

[c] *School of Pharmaceutical Sciences, University of Geneva, 1211 Geneva, Switzerland*

[d] *Institute of Pharmaceutical Sciences of Western Switzerland, University of Geneva, 1211 Geneva, Switzerland*

**e-mail:* francis.munier@fa2.ch or paul.dyson@epfl.ch

I. General Information ........ S2
II. Preparation of the perfluorinated Melphalan derivatives ........ S3
1. Synthesis of the Boc-protected Melphalan (***1***) ........ S3
2. General procedure A for the Steglich esterification of ***1*** (***2a***-***2f***) ........ S4
3. General Procedure B for the deprotection of ***2a-2f*** (***3a***-***3f***) ........ S11
III. X-Ray crystallography ........ S18
IV. Cytotoxicity studies ........ S21
1. Cell culture ........ S21
2. Presto Blue cell viability assay in Y79 after 24 h ........ S21
3. Presto Blue cell viability assay in Y79 and RPE1 cells at 72 h ........ S21
V. DNA binding ESI-MS study ........ S24
VI. NMR spectra of compounds ***1***, ***2a***-***2f***, ***3a***-***3f*** ........ S37
VII. Supplementary references ........ S56

# I. General Information

All the solvents and starting materials were purchased from various commercial sources and used without further purification unless otherwise stated. Melphalan was purchased from Carbosynth, UK, or Acros Organics, CH. The perfluorinated chains were obtained from either FluoroChem, UK, or ABCR, Germany.

NMR spectral data were obtained using various deuterated solvents. $^{1}$H, $^{13}$C and $^{19}$F-NMR spectra were recorded on Bruker 400 MHz spectrometer. Chemical shifts are in ppm relative to residual solvent signal. Data for $^{1}$H, $^{13}$C and $^{19}$F-NMR are reported as follows : chemical shift, multiplicity (s=singlet, d=doublet, dd= doublet of doublet, t=triplet, m=multiplet), integration, coupling constant (Hz) and atoms assignment. All raw FID files were processed, and the spectra analyzed, using the program MestReNOVA from Mestrelab Research S. L. Mass spectrometry spectra (MS) were acquired by the Mass spectrometry and Elemental Analysis platform at EPFL, using either a Thermo Orbitrap Elite instrument with an LTQ-Orbitrap analyser or a Waters XEVO G2-S QTOF instrument with a QTOF analyser.

Flash chromatography purifications were performed on a CombiFlash EZ Prep apparatus and the column chromatography with SilicaFlash P60, 40-63 µm (230-400 mesh) from Silicycle, CANADA using ACS grade solvent. All the synthetized compounds were monospot by TLC which were performed on pre-coated sheets ALUGRAM Xtra SIL G/UV$_{254}$ (Layer: 0.20 mm silica gel 60). All yields refer to chromatographically and spectroscopically ($^{1}$H, $^{13}$C and $^{19}$F NMR) pure material.

**Caution!** Melphalan is well known as highly hazardous and should be handled carefully in accordance with the safety data sheet provided by the commercial sources. The same precautions should be applied to its derivatives.

## II. Preparation of the perfluorinated Melphalan derivatives

### 1. Synthesis of the Boc-protected Melphalan (*1*)

**N-tert-Butoxycarbonyl-4-[bis(2-chloroethyl)amino]-L-phenylalanine (*1*)**

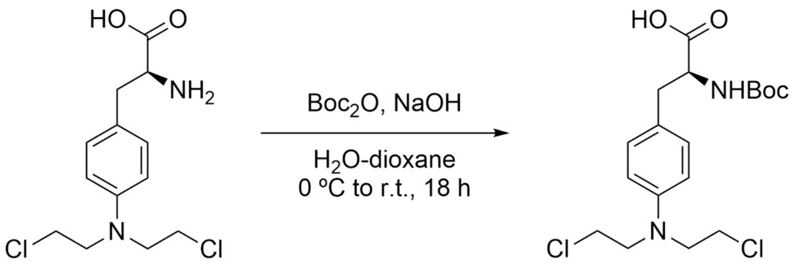


The intermediate **1** was prepared according to procedures found into existing literature[1] (70%).

**IR**: 3317, 1751, 1624, 769 $cm^{-1}$.

**$^1$H-NMR** (DMSO-$d_6$, 400 MHz): δ (ppm) = 12.52 (s, 1H, COO<u>H</u>), 7.08 (d, *J* = 8.5 Hz, 2H, arom. C<u>H</u>–C–N), 7.01 (d, *J* = 8.3 Hz, 1H, N<u>H</u>), 6.66 (d, *J* = 8.7 Hz, 2H, arom. $CH_2$–C–C<u>H</u>), 3.9 (m, 1H, C<u>H</u>CO), 3.70 (s, 8H, N(C<u>H</u>$_2$–C<u>H</u>$_2$)$_2$), 2.88 (dd, *J* = 13.9, 4.6 Hz, 1H, PhC<u>H</u>$_b$), 2.70 (dd, *J* = 13.8, 10.0 Hz, 1H, PhC<u>H</u>$_a$), 1.35 (s, 9H, C(C<u>H</u>$_3$)$_3$).

**$^{13}$C{$^1$H}-NMR** (101 MHz, DMSO-$d_6$) δ (ppm) = 174.23 (<u>C</u>OOH), 155.94 (<u>C</u>OO$^t$Bu), 145.30 (N–<u>C</u>=CH), 130.58 ($CH_2$–C=<u>C</u>H), 126.51 ($CH_2$–<u>C</u>=CH), 112.13 (<u>C</u>H–C–N), 78.47 (<u>C</u>($CH_3$)$_3$), 56.01 ($CH_2$–<u>C</u>H–NH), 52.62 (<u>C</u>H$_2$–Cl), 41.60 (N–<u>C</u>H$_2$–$CH_2$), 35.92 (Ph–<u>C</u>H$_2$), 28.64 (C(<u>C</u>H$_3$)$_3$).

**ESI MS**: *m/z* found 405.73 $[M+H]^+$, calcd 405.13; found 427.08 $[M+Na]^+$, calcd 427.12.

## 2. General procedure A for the Steglich esterification of *1* (*2a-2f*)

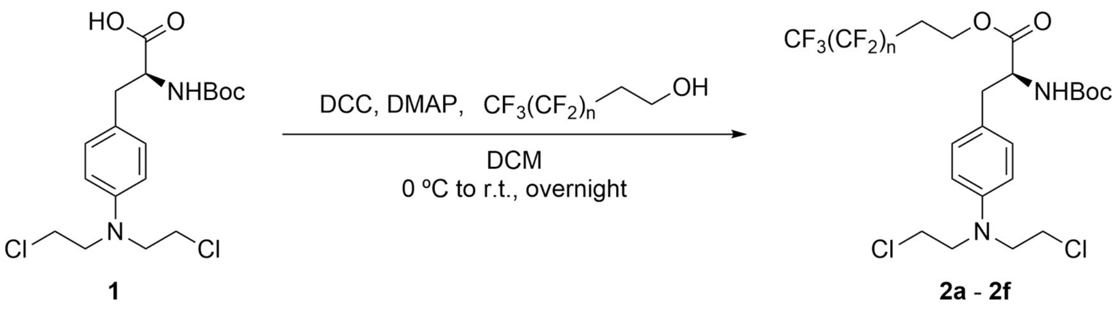


A flask was charged with the starting perfluorinated alcohol (0.99 mmol, 1 eq), N-tert-Butoxycarbonyl-4-[bis(2-chloroethyl)amino]-L-phenylalanine **1** (1 eq, 0.99 mmol), and 4-dimethylaminopyridine (DMAP: 0.1 mmol, 0.1 eq) in $CH_2Cl_2$ and cooled down with an ice bath. N,N'-dicyclohexylcarbodiimide (DCC: 1.1 mmol, 1.1 eq) was added and the mixture was stirred at room temperature overnight. The solution was washed with a saturated solution of $NaHCO_3$ (3 × $V_{DCM}$). The organic phase was dried over $Na_2SO_4$ and filtrated. The solvent was removed under reduced pressure and the residue was purified by column chromatography (elution: ethyl acetate/pentane, 0 - 30%) to afford the desired ester.

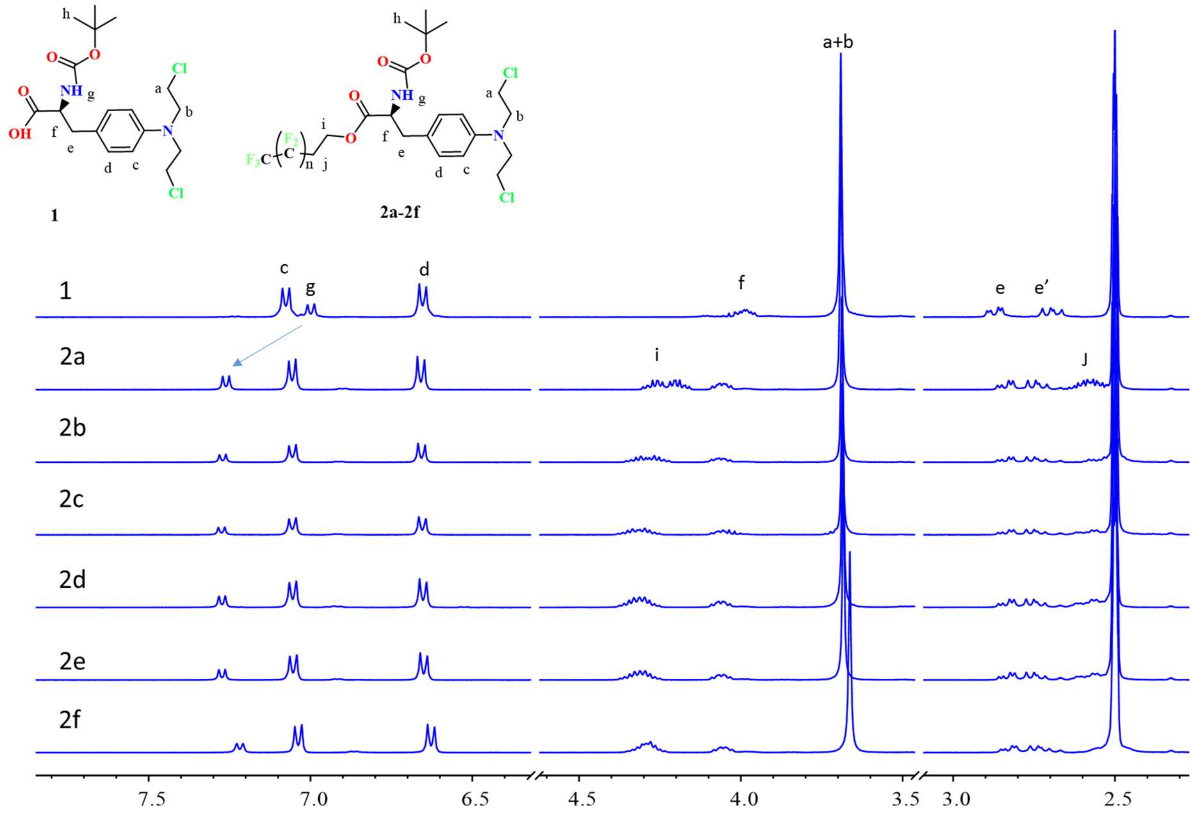


**Figure S1.** Comparison of the $^1$H NMR spectra of **1** and **2a-2f**.

**3,3,3-trifluoropropyl (*S*)-3-(4-(bis(2-chloroethyl)amino)phenyl)-2-((tert-butoxycarbonyl)amino)propanoate (*2a*)**

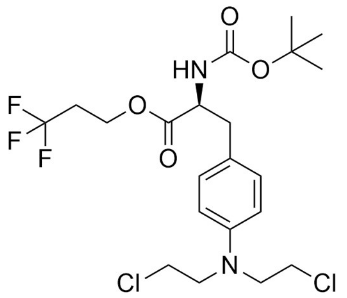

Prepared according to the General Procedure A with 3,3,3-trifluoro-1-propanol (238 mg, 184 μL) to afford **2c** as an oil (460 mg, 44%).

**$^1$H-NMR** (DMSO-$d_6$, 400 MHz): δ (ppm) = 7.26 (d, J = 7.8 Hz, 1H, N<u>H</u>), 7.05 (d, J = 8.6 Hz, 2H, arom. $CH_2$–C–C<u>H</u>), 6.66 (d, J = 8.7 Hz, 2H, arom. C<u>H</u>–C–N), 4.23 (m, 2H, $CH_2$–C<u>H</u>$_2$–O–CO), 4.06 (m, 1H, $CH_2$–C<u>H</u>–CO), 3.69 (s, 8H, N($CH_2$–$CH_2$)$_2$), 2.88 – 2.69 (m, 2H, Ph–$CH_2$), 2.67 – 2.54 (m, 2H, C<u>H</u>$_2$–$CH_2$–O–CO), 1.33 (s, 9H, C(C<u>H</u>$_3$)$_3$).
**$^{13}$C{$^1$H}-NMR** (101 MHz, DMSO-$d_6$) δ (ppm) = 171.97 (<u>C</u>OOCH$_2$), 155.37 (<u>C</u>OO$^t$Bu), 144.95 (N–<u>C</u>=CH), 130.06 ($CH_2$–C=<u>C</u>H), 127.79 (<u>C</u>F$_3$), 125.40 ($CH_2$–<u>C</u>=CH), 111.69 (<u>C</u>H–C–N), 78.27 (<u>C</u>($CH_3$)$_3$), 57.32 ($CH_2$–<u>C</u>H–CO), 55.60 ($CH_2$–<u>C</u>H–NH), 52.11 (<u>C</u>H$_2$–Cl), 41.08 (N–<u>C</u>H$_2$–$CH_2$), 35.26 (Ph–<u>C</u>H$_2$), 32.21 (q, $CF_3$–<u>C</u>H$_2$), 28.10 (C(<u>C</u>H$_3$)$_3$).
**$^{19}$F{$^1$H}-NMR** (376 MHz, DMSO-$d_6$): δ (ppm) = -63.47 (s, C<u>F</u>$_3$).
**ESI MS**: *m/z* found 501.33 [M+H]$^+$, calcd 501.15.

**3,3,4,4,4-pentafluorobutyl (*S*)-3-(4-(bis(2-chloroethyl)amino)phenyl)-2-((tert-butoxycarbonyl)amino)propanoate (*2b*)**

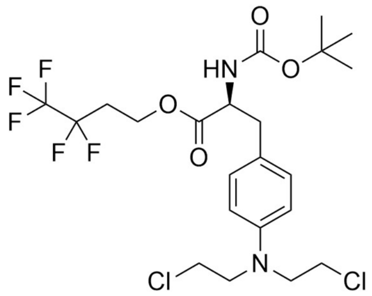


Prepared according to the General Procedure A with 3,3,4,4,4-pentafluoro-1-butanol (327 mg, 238 µL) to afford **2c** as an oil (319 mg, 29%).

**$^{1}$H-NMR** (DMSO-*d₆*, 400 MHz): δ (ppm) = 7.27 (d, J = 7.8 Hz, 1H, N<u>H</u>), 7.06 (d, J = 8.7 Hz, 2H, arom. $CH_2$–C–C<u>H</u>), 6.66 (d, J = 8.7 Hz, 2H, arom. C<u>H</u>–C–N), 4.29 (m, 2H, $CH_2$–C<u>H</u>$_2$–O–CO), 4.06 (m, 1H, $CH_2$–C<u>H</u>–CO), 3.69 (s, 8H, N($CH_2$–$CH_2$)$_2$), 2.89 – 2.69 (m, 2H, Ph–$CH_2$), 2.61 – 2.56 (m, 2H, C<u>H</u>$_2$–$CH_2$–O–CO), 1.33 (s, 9H, C(C<u>H</u>$_3$)$_3$).

**$^{13}$C{$^{1}$H}-NMR** (101 MHz, DMSO-*d₆*) δ (ppm) = 171.98 (<u>C</u>OO$CH_2$), 155.36 (<u>C</u>OO$^t$Bu), 144.96 (N–<u>C</u>=CH), 130.05 ($CH_2$–C=<u>C</u>H), 128.28 (<u>C</u>$F_3$), 125.38 ($CH_2$–<u>C</u>=CH), 111.67 (<u>C</u>H–C–N), 78.27 (<u>C</u>($CH_3$)$_3$), 56.45 ($CH_2$–<u>C</u>H–CO), 55.61 ($CH_2$–<u>C</u>H–NH), 52.10 (<u>C</u>$H_2$–Cl), 41.07 (N–<u>C</u>$H_2$–$CH_2$), 35.25 (Ph–<u>C</u>$H_2$), 29.14 (t, $CF_2$–<u>C</u>$H_2$), 28.09 (C(<u>C</u>$H_3$)$_3$).

**$^{19}$F{$^{1}$H}-NMR** (400 MHz, DMSO-*d₆*): δ (ppm) = -84.92 (s, C<u>F</u>$_3$), -116.36 (s, C<u>F</u>$_2$).

**ESI MS**: *m/z* found 551.33 [M+H]$^+$, calcd 551.15; found 573.25 [M+Na]$^+$, calcd 573.13.

**3,3,4,4,5,5,6,6,6-nonafluorohexyl (*S*)-3-(4-(bis(2-chloroethyl)amino)phenyl)-2-((tert-butoxycarbonyl)amino)propanoate (*2c*)**

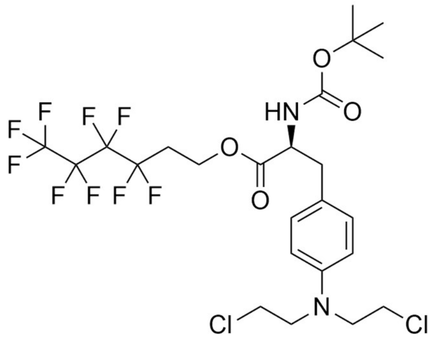

Prepared according to the General Procedure A with 1,1,2,2-tetrahydroperfluorohexan-1-ol (589 mg, 377 µL) to afford **2c** as an oil (416 mg, 32%).

**$^1$H-NMR** (DMSO-$d_6$, 400 MHz): δ (ppm) = 7.27 (d, J = 7.7 Hz, 1H, N<u>H</u>), 7.06 (d, J = 8.2 Hz, 2H, arom. $CH_2$–C–C<u>H</u>), 6.65 (d, J = 8.4 Hz, 2H, arom. C<u>H</u>–C–N), 4.31 (m, 2H, $CH_2$–C<u>H</u>$_2$–O–CO), 4.12 – 4.00 (m, 1H, $CH_2$–C<u>H</u>–CO), 3.69 (s, 8H, N($CH_2$–$CH_2$)$_2$), 2.84 (dd, *J* = 13.8, 5.5 Hz, 1H, Ph–<u>CH</u>$_a$), 2.74 (dd, *J* = 13.8, 9.6 Hz, 1H, Ph–C<u>H</u>$_b$), 2.59 (m, 2H, C<u>H</u>$_2$–$CH_2$–O–CO), 1.33 (s, 9H, C(C<u>H</u>$_3$)$_3$).

**$^{13}$C{$^1$H}-NMR** (101 MHz, DMSO-$d_6$): δ (ppm) = 171.99 (<u>C</u>OOCH$_2$), 155.35 (<u>C</u>OO$^t$Bu), 144.95 (N–<u>C</u>=CH), 130.05 ($CH_2$–C=<u>C</u>H), 130.11 and 125.23 (-<u>C</u>F$_2$<u>C</u>F$_3$), 125.37 ($CH_2$–<u>C</u>=CH), 111.65 (<u>C</u>H–C–N), 78.26 (<u>C</u>($CH_3$)$_3$), 56.38 ($CH_2$–<u>C</u>H–CO), 55.61 ($CH_2$–<u>C</u>H–NH), 52.10 (<u>C</u>H$_2$–Cl), 41.05 (N–<u>C</u>H$_2$–$CH_2$), 35.25 (Ph–<u>C</u>H$_2$), 29.32 (t, $CF_3$–<u>C</u>H$_2$), 28.06 (C(<u>C</u>H$_3$)$_3$).

**$^{19}$F{$^1$H}-NMR** (376 MHz, DMSO-$d_6$): δ (ppm) = -80.51 (m, C<u>F</u>$_3$), -113.00 (m, $CF_3$-$CF_2$-C<u>F</u>$_2$), 124.09 (m, $CF_3$-C<u>F</u>$_2$), -125.68 (m, $CH_2$-C<u>F</u>$_2$).

**ESI MS**: *m/z* found 651.33 [M+H]$^+$, calcd 651.14; found 673.25 [M+Na]$^+$, calcd 673.13.

**3,3,4,4,5,5,6,6,7,7,8,8,8-tridecafluorooctyl (*S*)-3-(4-(bis(2-chloroethyl)amino)phenyl)-2-((tert-butoxycarbonyl)amino)propanoate (*2d*)**

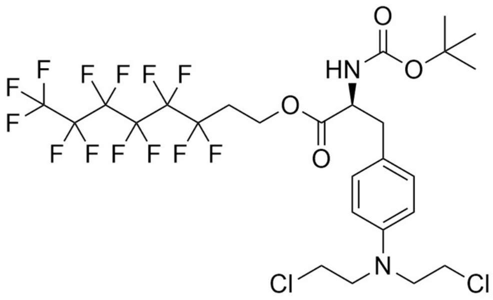


Prepared according to the General Procedure A with 1,1,2,2-tetrahydroperfluorooctanol (370.5 mg, 224 µL) to afford **2d** as a white solid (305 mg, 40%).

**$^1$H-NMR** (DMSO-*d*$_6$, 400 MHz): δ (ppm) = 7.28 (d, J = 7.8 Hz, 1H, $N\underline{H}$), 7.05 (d, J = 8.3 Hz, 2H, $CH_2–C–C\underline{H}$), 6.65 (d, J = 8.6 Hz, 2H, $C\underline{H}–C–N$), 4.31 (m, 2H, $CH_2–C\underline{H_2}–O–CO$), 4.06 (m, 1H, $CH_2–C\underline{H}–CO$), 3.69 (s, 8H, $N(CH_2–CH_2)_2$), 2.84 (dd, 1H, J = 13.9, 5.5 Hz, $Ph–C\underline{H_a}$) 2.75 (dd, J = 13.8, 9.5 Hz, 1H, $Ph–C\underline{H_b}$), 2.58 (m, 2H, $C\underline{H_2}–CH_2–O–CO$), 1.33 (s, 9H, $C(C\underline{H_3})_3$).
**$^{13}$C{$^1$H}-NMR** (101 MHz, DMSO-*d*$_6$) δ (ppm) = 171.99 ($\underline{C}OOCH_2$), 155.83 ($\underline{C}OO^tBu$), 145.40 ($N–\underline{C}=CH$), 130.53 ($CH_2–C=\underline{C}H$), 125.82 ($CH_2–\underline{C}=CH$), 118.23 ($CH_2–\underline{C}F_2$), 117.12 ($\underline{C}F_3$), 112.01 ($\underline{C}H–C–N$), 111.02 ($CH_2–CF_2–\underline{C}F_2$), 110.77 ($CF_3–CF_2–\underline{C}F_2$), 110.22 ($CF_3–\underline{C}F_2$), 108.48 ($CF_3–CF_2–CF_2–\underline{C}F_2$), 78.74 ($\underline{C}(CH_3)_3$), 56.88 ($CH_2–\underline{C}H–CO$), 56.10 ($CH_2–\underline{C}H–NH_3$) 52.55 ($N–\underline{C}H_2–CH_2$), 41.50 ($\underline{C}H_2–Cl$), 35.70 ($Ph–\underline{C}H_2$), 29.89 ($CF_2–\underline{C}H_2$), 28.52 ($C(\underline{C}H_3)_3$).
**$^{19}$F{$^1$H}-NMR** (376 MHz, DMSO-*d*$_6$): δ (ppm) = -80.24 (t, J = 10.Hz, 3F, $C\underline{F_3}$), -112.79 (m, 2F, $CH_2–C\underline{F_2}$), -121.74 (m, 2F, $CH_2–CF_2–C\underline{F_2}$), -122.68 (s, 2F, $CF_3–C\underline{F_2}$), -123.21 (m, 2F, $CF_3–CF_2–C\underline{F_2}$), -125.79 (m, 2F, $CF_3–CF_2–CF_2–C\underline{F_2}$).
**ESI MS**: *m/z* found 751.25 [M+H]$^+$, calcd 751.14; found 773.08 [M+Na]$^+$, calcd 773.12.

**3,3,4,4,5,5,6,6,7,7,8,8,9,9,10,10,10-heptadecafluorodecyl (*S*)-3-(4-(bis(2-chloroethyl)amino)phenyl)-2-((tert-butoxycarbonyl)amino)propanoate (*2e*)**

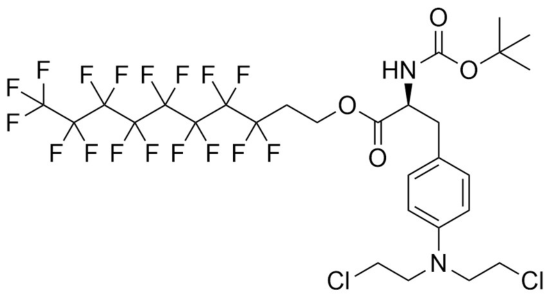

Prepared according to the General Procedure A with 1,1,2,2-tetrahydroperfluorodecanol (440.7 mg) to afford **2e** as a white solid (477 mg, 59 %).

**$^1$H-NMR** (DMSO-$d_6$, 400 MHz): δ (ppm) = 7.28 (d, J = 7.8 Hz, 1H, NH), 7.06 (d, J = 8.7 Hz, 2H, arom. $CH_2–C–C\underline{H}$), 6.66 (d, J = 8.7 Hz, 2H, arom. $C\underline{H}–C–N$), 4.32 (m, 2H, $CH_2–C\underline{H}_2–O–CO$), 4.07 (dt, J = 4.0, 2.7 Hz, 1H, $CH_2–C\underline{H}–NH_3$), 3.69 (br. s, 8H, $N(C\underline{H}_2–C\underline{H}_2)_2$), 2.67-2.88 (m, 2H, $PhC\underline{H}_2$), 2.58 (m, 2H, $C\underline{H}_2–CH_2–O–CO$), 1.34 (s, 9H, $C(C\underline{H}_3)_3$).
**$^{13}$C{$^1$H}-NMR** (101 MHz, DMSO-$d_6$) δ (ppm) = 172.47 ($\underline{C}OOCH_2$), 155.82, ($\underline{C}OO^tBu$), 145.43 ($N–\underline{C}=CH$), 130.53 ($CH_2–C=\underline{C}H$), 125.85 ($CH_2–\underline{C}=CH$), 118.35 ($CH_2–\underline{C}F_2$), 116.99 ($\underline{C}F_3$), 112.11 ($\underline{C}H–C–N$), -110.65 ($CH_2–CF_2–\underline{C}F_2–\underline{C}F_2$), 111.10 ($CF_3–\underline{C}F_2$), 110.82 ($CF_3–CF_2–\underline{C}F_2$), 108.27 ($CF_3–CF_2–CF_2–\underline{C}F_2$), 78.73 ($\underline{C}(CH_3)_3$), 56.88 ($CH_2–\underline{C}H–CO$), 56.10 ($CH_2–\underline{C}H–NH_3$) 52.58 ($N–\underline{C}H_2–CH_2$), 41.50 ($\underline{C}H_2–Cl$), 35.73 ($Ph–\underline{C}H_2$), 29.91 ($CF_2–\underline{C}H_2$), 28.52 ($C(\underline{C}H_3)_3$).
**$^{19}$F{$^1$H}-NMR** (376 MHz, DMSO-$d_6$): δ (ppm) = -80.23 (t, J = 11.3 Hz, 3F, $C\underline{F}_3$), -112.80 (t, J=15.7 Hz, 2F, $CH_2–C\underline{F}_2$), -121.53 (m, 2F, $CF_3–C\underline{F}_2$), -121.76 (br m, 4F, $CH_2–CF_2–C\underline{F}_2–C\underline{F}_2$), -122.54 (s, 2F, $CF_3–CF_2–C\underline{F}_2$), -123.21 (m, 2F, $CF_3–CF_2–C\underline{F}_2$), -125.79 (m, 2F, $CF_3–CF_2–CF_2–C\underline{F}_2$).
**ESI MS**: *m/z* found 851.25 $[M+H]^+$, calcd 851.13; found 873.08 $[M+Na]^+$, calcd 873.11.

**3,3,4,4,5,5,6,6,7,7,8,8,9,9,10,10,11,11,12,12,12-henicosafluorododecyl (*S*)-3-(4-(bis(2-chloroethyl)amino)phenyl)-2-((tert-butoxycarbonyl)amino)propanoate (*2f*)**

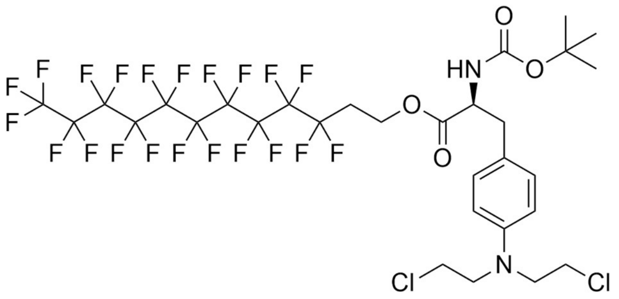


Prepared according to the General Procedure A with 1,1,2,2-Tetrahydroperfluorododecanol (531.4 mg) to afford **2f** as a white solid (475 mg, 53%).

**$^1$H-NMR** (DMSO-$d_6$, 400 MHz): δ (ppm) = 7.23 (d, J = 7.8 Hz, 1H, NH), 7.05 (d, J = 8.3 Hz, 2H, arom. $CH_2$–C–C$\underline{H}$), 6.64 (d, J = 8.4 Hz, 2H, arom. C$\underline{H}$–C–N), 4.30 (m, 2H, $CH_2$–C$\underline{H}_2$–O–CO), 4.06 ( m, 1H, $CH_2$–C$\underline{H}$–$NH_3$), 3.67 (br s, 8H, N(C$\underline{H}_2$–C$\underline{H}_2$)$_2$), 2.67-2.86 (m, 2H, Ph–C$\underline{H}_2$) ), 2.56 (m, 2H, C$\underline{H}_2$–$CH_2$–O–CO), 1.31 (s, 9H, C(C$\underline{H}_3$)$_3$).

**$^{13}$C{$^1$H}-NMR** (101 MHz, DMSO-$d_6$) δ (ppm) = 172.46 ($\underline{C}OOCH_2$), 155.82, ($\underline{C}OO^tBu$), 145.43 (N–$\underline{C}$=CH), 130.53 ($CH_2$–C=$\underline{C}$H), 125.85 ($CH_2$–$\underline{C}$=CH), 118.30 ($CH_2$–$\underline{C}F_2$), 116.99 ($\underline{C}F_3$), 112.10 ($\underline{C}$H–C–N), 110.83 ($CH_2$–$CF_2$–$\underline{C}F_2$), 110.74 ($CH_2$–$CF_2$–$\underline{C}F_2$–$\underline{C}F_2$–$\underline{C}F_2$–$\underline{C}F_2$), 110.62 ($CF_3$–$\underline{C}F_2$), 110.52 ($CF_3$–$CF_2$–$\underline{C}F_2$), 108.25 ($CF_3$–$CF_2$–$CF_2$–$\underline{C}F_2$), 78.73 ($\underline{C}(CH_3)_3$), 56.87 ($CH_2$–$\underline{C}$H–CO), 56.10 ($CH_2$–$\underline{C}$H–$NH_3$) 52.57 (N–$\underline{C}H_2$–$CH_2$), 41.49 ($\underline{C}H_2$–Cl), 35.74 (Ph–$\underline{C}H_2$), 29.92 ($CF_2$–$\underline{C}H_2$), 28.51 (C($\underline{C}H_3$)$_3$).

**$^{19}$F{$^1$H}-NMR** (376 MHz, DMSO-$d_6$): δ (ppm) = -80.41 (s, 3F, C$\underline{F}_3$), -112.89 (s, 2F, $CH_2$–C$\underline{F}_2$), -121.63 (br m, 8F, $CH_2$–$CF_2$–C$\underline{F}_2$–C$\underline{F}_2$–C$\underline{F}_2$–C$\underline{F}_2$), -121.80 (s, 2F, $CF_3$–C$\underline{F}_2$), -122.60 (s, 2F, $CF_3$–$CF_2$–C$\underline{F}_2$), -123.28 (s, 2F, $CF_3$–$CF_2$–C$\underline{F}_2$), -125.94 (s, 2F, $CF_3$–$CF_2$–$CF_2$–C$\underline{F}_2$).

**ESI MS**: *m/z* found 951.25 $[M+H]^+$, calcd 951.12; found 973.08 $[M+Na]^+$, calcd 973.11.

## 3. General Procedure B for the deprotection of *2a-2f* (*3a-3f*)

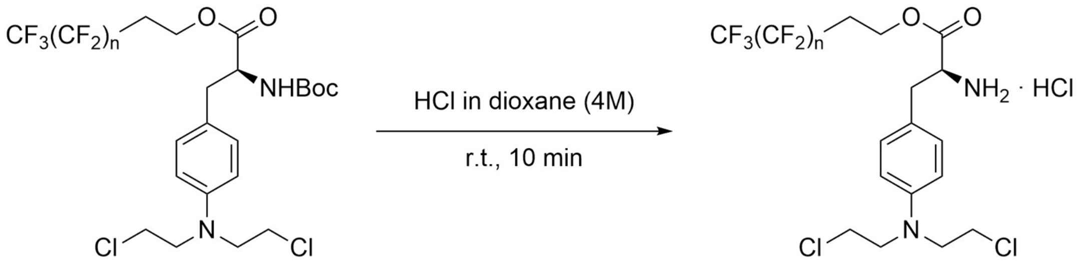


A solution of hydrochloric acid in dioxane (4M) was added to **2a-2f** (0.4 mmol) and stirred at room temperature for 10 min. Excess HCl and the solvent was evaporated under reduce pressure. The residue was then washed several times with ether and dried under vacuum to obtain **3a-3f**.

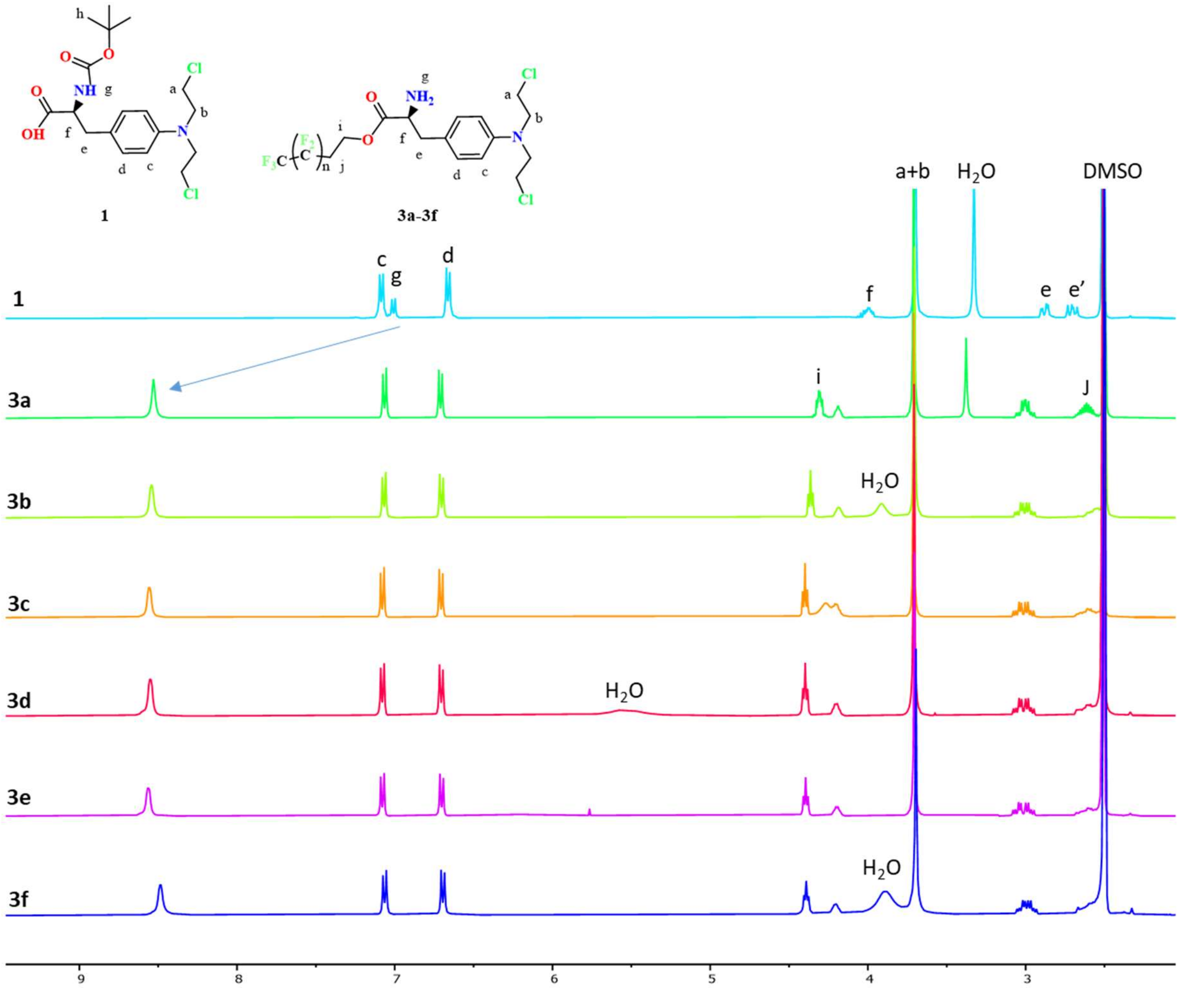


**Figure S2.** Comparison of the $^1$H NMR spectra of **1** and **3a-3f**.

**3,3,3-trifluoropropyl (*S*)-2-amino-3-(4-(bis(2-chloroethyl)amino)phenyl)propanoate hydrochloride (*3a*)**

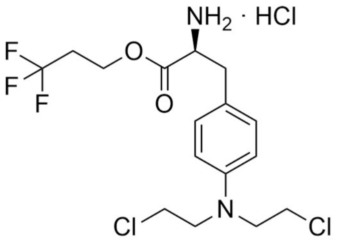


Prepared according to the General Procedure B with **2a** to afford **3a** as white solid (259 mg, 85%)

**$^1$H-NMR** (DMSO-$d_6$, 400 MHz): δ (ppm) = 8.53 (s, 3H, $NH_3$), 7.07 (d, J = 8.7 Hz, 2H, arom $CH_2$–C–C<u>H</u>), 6.71 (d, J = 8.8 Hz, 2H, arom. C<u>H</u>–C–N), 4.38 – 4.23 (m, 2H, $CH_2$–C<u>H</u>$_2$–O–CO), 4.19 (t, J = 6.1 Hz, 1H, $CH_2$–C<u>H</u>–$NH_3$), 3.71 (br s, 8H, N(C<u>H</u>$_2$–C<u>H</u>$_2$)$_2$), 3.00 (m, 2H, Ph–C<u>H</u>$_2$), 2.62 (m, 2H, C<u>H</u>$_2$–$CH_2$–O–CO).

**$^{13}$C{$^1$H}-NMR** (101 MHz, DMSO-$d_6$) δ (ppm) = 168.88 (<u>C</u>OO$CH_2$), 145.60, (N–<u>C</u>=CH), 130.46 (<u>C</u>H, arom $CH_2$–C=<u>C</u>H), 126.29 (q, *J* = 276.9 Hz, <u>C</u>$F_3$), 122.00 ($CH_2$–<u>C</u>=CH), 111.98 (<u>C</u>H, arom. <u>C</u>H–C–N), 58.45 ($CH_2$–<u>C</u>H–CO), 53.29 ($CH_2$–<u>C</u>H–$NH_3$) 52.03 (N–<u>C</u>H$_2$–$CH_2$), 41.07 (<u>C</u>H$_2$–Cl), 34.83 (Ph–<u>C</u>H$_2$), 32.01 (q, J = 28.3 Hz, $CF_2$–<u>C</u>H$_2$).

**$^{19}$F{$^1$H}-NMR** (400 MHz, DMSO-$d_6$): δ (ppm) = -63.52 (s, 3F, C<u>F</u>$_3$).

**ESI MS**: *m/z* found 401.17 [M−Cl]$^+$, calcd 401.10; found 423.25[M−HCl+Na]$^+$, calcd 423.08.

**3,3,4,4,4-pentafluorobutyl (*S*)-2-amino-3-(4-(bis(2-chloroethyl)amino)phenyl)propanoate hydrochloride (*3b*)**

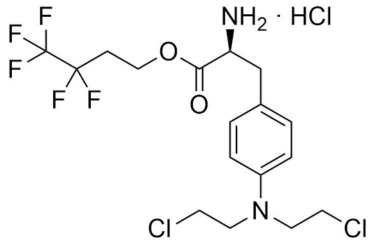


Prepared according to the General Procedure B with **2b** to afford **3b** as white solid (174 mg, 79%)

**$^{1}$H-NMR** (DMSO-$d_6$, 400 MHz): δ (ppm) = 8.57 – 8.51 (m, 2H, N$\underline{H}_3$), 7.07 (d, *J* = 8.4 Hz, 2H, arom. $CH_2$–C–C$\underline{H}$), 6.70 (d, *J* = 8.5 Hz, 2H, arom. C$\underline{H}$–C–N), 4.36 (t, *J* = 6.0 Hz, 2H, $CH_2$–C$\underline{H}_2$–O–CO), 4.22 – 4.14 (m, 1H, $CH_2$–C$\underline{H}$–$NH_3$), 3.71 (s, 8H, N(C$\underline{H}_2$–C$\underline{H}_2$)$_2$), 3.01 (qd, *J* = 14.2, 6.6 Hz, 2H, Ph–C$\underline{H}_2$), 2.68 – 2.53 (m, 2H, C$\underline{H}_2$–$CH_2$–O–CO).
**$^{13}$C{$^{1}$H}-NMR** (101 MHz, DMSO-$d_6$) δ (ppm) = 168.91 ($\underline{C}$OO$CH_2$), 145.96 (N–$\underline{C}$=CH), 130.45 ($CH_2$–C=$\underline{C}$H), 116.13 (q, *J* = 196.8 Hz, $\underline{C}F_3$), 122.04 ($CH_2$–$\underline{C}$=CH), 111.93 ($\underline{C}$H–C–N), 57.55 ($CH_2$–$\underline{C}$H–CO), 53.31 ($CH_2$–$\underline{C}$H–NH), 52.02 ($\underline{C}H_2$–Cl), 41.04 (N–$\underline{C}H_2$–$CH_2$), 34.84 (Ph–$\underline{C}H_2$), 28.89 (t, *J* = 20.9 Hz, $CF_2$–$\underline{C}H_2$
**$^{19}$F{$^{1}$H}-NMR** (400 MHz, DMSO-$d_6$): δ (ppm) = -84.94 (s, C$\underline{F}_3$), -116.50 (s, C$\underline{F}_2$).
**ESI MS**: *m/z* found 451.17 [M−Cl]$^+$, calcd 451.10; found 473.17 [M−HCl+Na]$^+$, calcd 473.08.

**3,3,4,4,5,5,6,6,6-nonafluorohexyl (*S*)-2-amino-3-(4-(bis(2-chloroethyl)amino)phenyl)propanoate hydrochloride (*3c*)**

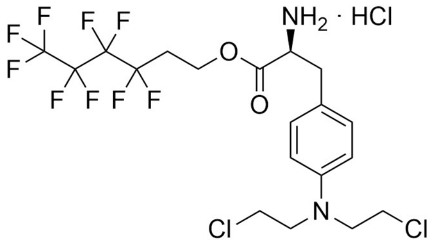

Prepared according to the General Procedure B with **2c** to afford **3c** as white solid (189 mg, 88%).

**$^1$H-NMR** (DMSO-*d*$_6$, 400 MHz): δ (ppm) = 8.56 (d, *J* = 5.2 Hz, 3H, $N\underline{H}_3$), 7.08 (d, J = 8.7 Hz, 2H, arom. $CH_2$–C–C$\underline{H}$), 6.71 (d, J = 8.7 Hz, 2H, arom. C$\underline{H}$–C–N), 4.40 (t, *J* = 6.0 Hz, 2H, $CH_2$–C$\underline{H}_2$–O–CO), 4.20 (q, *J* = 6.2, 5.7 Hz, 1H, $CH_2$–C$\underline{H}$–CO), 3.71 (s, 8H, N(C$\underline{H}_2$–C$\underline{H}_2$)$_2$, 3.01 (qd, *J* = 14.2, 6.6 Hz, 2H, Ph–C$\underline{H}_2$), 2.71 – 2.52 (m, 2H, C$\underline{H}_2$–$CH_2$–O–CO).
**$^{13}$C{$^1$H}-NMR** (101 MHz, DMSO-*d*$_6$): δ (ppm) = 168.92 ($\underline{C}OOCH_2$), 145.60 (N–$\underline{C}$=CH), 130.46 ($CH_2$–C=$\underline{C}$H), 122.08 ($CH_2$–$\underline{C}$=CH), 111.89 ($\underline{C}$H–C–N), 57.49 ($CH_2$–$\underline{C}$H–CO), 53.30 ($CH_2$–$\underline{C}$H–NH), 52.02 ($\underline{C}H_2$–Cl), 41.00 (N–$\underline{C}H_2$–$CH_2$), 34.84(Ph–$\underline{C}H_2$), 29.11 (t, *J* = 20.6 Hz, $CF_3$–$\underline{C}H_2$).
**$^{19}$F{$^1$H}-NMR** (376 MHz, DMSO-*d*$_6$): δ (ppm) = -80.48 (m, C$\underline{F}_3$), -113.c00 (m, $CF_3$-$CF_2$-C$\underline{F}_2$), -124.09 (m, $CF_3$-C$\underline{F}_2$), -125.68 (m, $CH_2$-C$\underline{F}_2$).
**ESI MS**: *m/z* found 551.09 [M−Cl]$^+$, calcd 551.09.

**3,3,4,4,5,5,6,6,7,7,8,8,8-tridecafluorooctyl (*S*)-2-amino-3-(4-(bis(2-chloroethyl)amino)phenyl)propanoate hydrochloride (*3d*)**

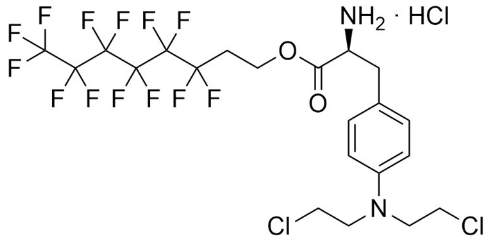

Prepared according to the General Procedure B with **2d** to afford **3d** as white solid (190 mg, 69%).

**$^{1}$H-NMR** (DMSO-*d6*, 400 MHz): δ (ppm) = 8.55 (m, 3H, $NH_3$), 7.08 (d, J = 8.7 Hz, 2H, arom. $CH_2$–C–C<u>H</u>), 6.71 (d, J = 8.8 Hz, 2H, arom. C<u>H</u>–C–N), 4.40 (t, J = 6.0 Hz, 2H, $CH_2$–C<u>H</u>$_2$–O–CO), 4.20 (m, 1H, $CH_2$–C<u>H</u>–$NH_3$), 3.71 (br s, 8H, N(C<u>H</u>$_2$–C<u>H</u>$_2$)$_2$), 3.01 (m, 2H, Ph–C<u>H</u>$_2$), 2.53 (m, 2H, C<u>H</u>$_2$–$CH_2$–O–CO).

**$^{13}$C{$^{1}$H}-NMR** (101 MHz, DMSO-*d6*) δ (ppm) = 169.39 (<u>C</u>OOCH$_2$), 146.06, (N–<u>C</u>=CH), 130.93 (<u>C</u>H, arom. $CH_2$–C=<u>C</u>H), 122.55 ($CH_2$–<u>C</u>=CH), 112.34 (<u>C</u>H, arom. <u>C</u>H–C–N), 118.11 ($CH_2$–<u>C</u>F$_2$), 117.09 (<u>C</u>F$_3$), 111,05 ($CH_2$–$CF_2$–<u>C</u>F$_2$), 110.79 ($CF_3$–$CF_2$–<u>C</u>F$_2$), 110.20 ($CF_3$–<u>C</u>F$_2$), 108.37 ($CF_3$–$CF_2$–$CF_2$–<u>C</u>F$_2$), 57.98 ($CH_2$–<u>C</u>H–CO), 53.78 ($CH_2$–<u>C</u>H–$NH_3$) 52.48 (N–<u>C</u>H$_2$–$CH_2$), 41.45 (<u>C</u>H$_2$–Cl), 35.32 (Ph–<u>C</u>H$_2$), 29.68 (t , *J* = 19.1 Hz, $CF_2$–<u>C</u>H$_2$).

**$^{19}$F{$^{1}$H}-NMR** (376 MHz, DMSO-*d6*): δ (ppm) = -80.21 (t, 3F, C<u>F</u>$_3$), -112.80 (m, 2F, $CH_2$–C<u>F</u>$_2$), -121.69 (m, 2F, $CH_2$–$CF_2$–C<u>F</u>$_2$), -122.62 (m, 2F, $CF_3$–C<u>F</u>$_2$), -123.17 (m, 2F, $CF_3$–$CF_2$–C<u>F</u>$_2$), -125.74 (m, 2F, $CF_3$–$CF_2$–$CF_2$–C<u>F</u>$_2$).

**ESI MS**: *m/z* found 651.08 [M−Cl]$^+$, calcd 651.08.

**3,3,4,4,5,5,6,6,7,7,8,8,9,9,10,10,10-heptadecafluorodecyl (*S*)-2-amino-3-(4-(bis(2-chloroethyl)amino)phenyl)propanoate hydrochloride (*3e*)**

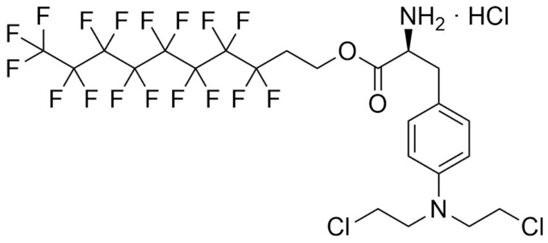


Prepared according to the General Procedure B with **2e** to afford **3e** as white solid (211 mg, 67%).

**$^1$H-NMR** (DMSO-$d_6$, 400 MHz): δ (ppm) = 8.57 (s, 3H, $NH_3$), 7.08 (d, J = 8.8 Hz, 2H, arom. $CH_2$–C–C$\underline{H}$), 6.70 (d, J = 8.3 Hz, 2H, arom. C$\underline{H}$–C–N), 4.39 (t, J = 6.1 Hz, 2H, $CH_2$–C$\underline{H}_2$–O–CO), 4.20 (m, 1H, $CH_2$–C$\underline{H}$–$NH_3$), 3.72 (s, 8H, N(C$\underline{H}_2$–C$\underline{H}_2$)$_2$), 3.01 (m, 2H, Ph–C$\underline{H}_2$) ), 2.60 (m, 2H, C$\underline{H}_2$–$CH_2$–O–CO).
**$^{13}$C{$^1$H}-NMR** (101 MHz, DMSO-$d_6$) δ (ppm) = 169.39 ($\underline{C}$OO–$CH_2$), 146.05, (N–$\underline{C}$=CH), 130.92 ($\underline{C}$H, arom. $CH_2$–C=$\underline{C}$H), 122.57 ($CH_2$–$\underline{C}$=CH), 112.33 ($\underline{C}$H, arom. $\underline{C}$H–C–N ), 118.24 ($CH_2$–$\underline{C}F_2$), 117.01 ($\underline{C}F_3$), 110.80 ($CF_3$–$CF_2$–$\underline{C}F_2$), 110.66 ($CH_2$–$CF_2$–$\underline{C}F_2$–$\underline{C}F_2$), 111.10 ($CF_3$–$CF_2$–$CF_2$–$CF_2$–$\underline{C}F_2$), 110.14 ($CF_3$–$\underline{C}F_2$), 108.29 ($CF_3$–$CF_2$–$CF_2$–$\underline{C}F_2$), 57.98 ($CH_2$–$\underline{C}$H–CO), 53.78($CH_2$–$\underline{C}$H–$NH_3$) 52.49 (N–$\underline{C}H_2$–$CH_2$), 41.43 ($\underline{C}H_2$–Cl), 35.32 (Ph–$\underline{C}H_2$), 29.68 ($CF_2$–$\underline{C}H_2$).
**$^{19}$F{$^1$H}-NMR** (376 MHz, DMSO-$d_6$): δ (ppm) = -80.18 (t, J = 11.3 Hz, 3F, C$\underline{F}_3$), -112.79 (t, J=15.7 Hz, 2F, $CH_2$–C$\underline{F}_2$), -121.44 (m, 2F, $CF_3$–$CF_2$–$CF_2$–$CF_2$–C$\underline{F}_2$),), -121.67 (br m, 4F, $CH_2$–$CF_2$–C$\underline{F}_2$–C$\underline{F}_2$), -122.44 (m, 2F, $CF_3$–C$\underline{F}_2$), -123.21 (m, 2F, $CF_3$–$CF_2$–C$\underline{F}_2$), -125.79 (m, 2F, $CF_3$–$CF_2$–$CF_2$–C$\underline{F}_2$).
**ESI MS**: *m/z* found 751.08 [M−Cl]$^+$, calcd 751.08.

**3,3,4,4,5,5,6,6,7,7,8,8,9,9,10,10,11,11,12,12,12-henicosafluorododecyl (*S*)-2-amino-3-(4-(bis(2-chloroethyl)amino)phenyl)propanoate hydrochloride *(3f)***

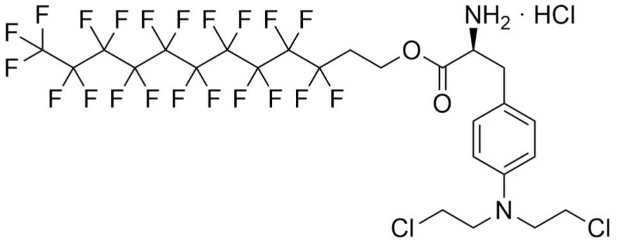

Prepared according to the General Procedure B with **2f** to give **3f** as white solid (150 mg, 42%).

**$^{1}$H-NMR** (DMSO, 400 MHz): δ (ppm) = 8.49 (s, 3H, $NH_3$), 7.07 (d, J = 8.8 Hz, 2H, arom $CH_2$–C–C$\underline{H}$), 6.70 (d, J = 8.7 Hz, 2H, arom. C$\underline{H}$–C–N), 4.40 (t, J = 6.6 Hz, 2H, $CH_2$–C$\underline{H}_2$–O–CO), 4.21 (m, 1H, $CH_2$–C$\underline{H}$–$NH_3$), 3.71 (s, 8H, N(C$\underline{H}_2$–C$\underline{H}_2$)$_2$), 3.00 (m, 2H, Ph–C$\underline{H}_2$) ), 2.61 (m, 2H, C$\underline{H}_2$–$CH_2$–O–CO).
**$^{13}$C{$^{1}$H}-NMR** (101 MHz, DMSO) δ (ppm) = 169.41 ($\underline{C}$OO–$CH_2$), 146.07, (N–$\underline{C}$=CH), 130.92 ($\underline{C}$H, arom $CH_2$–C=$\underline{C}$H), 122.50 ($CH_2$–$\underline{C}$=CH), 112.33 ($\underline{C}$H, arom $\underline{C}$H–C–N ), 118.29 ($CH_2$–$\underline{C}F_2$), 116.99 ($\underline{C}F_3$), 111.79 ($CF_3$–$CF_2$–$CF_2$–$CF_2$–$\underline{C}F_2$), 110.76 ($CH_2$–$CF_2$–$\underline{C}F_2$–$\underline{C}F_2$–$\underline{C}F_2$–$\underline{C}F_2$), 110.11 ($CF_3$–$\underline{C}F_2$), 110.60 ($CF_3$–$CF_2$–$\underline{C}F_2$), 108.27 ($CF_3$–$CF_2$–$CF_2$–$\underline{C}F_2$), 58.01 ($CH_2$–$\underline{C}$H–CO), 53.77 ($CH_2$–$\underline{C}$H–$NH_3$) 52.47 (N–$\underline{C}H_2$–$CH_2$), 41.42 ($\underline{C}H_2$–Cl), 35.34 (Ph–$\underline{C}H_2$), 29.61 ($CF_2$–$\underline{C}H_2$).
**$^{19}$F{$^{1}$H}-NMR** (376 MHz, DMSO-d6): δ (ppm) = -80.19 (t, 3H, C$\underline{F}_3$), -112.81 (m, 2F, $CH_2$–C$\underline{F}_2$), -121.45 (m, 8F, $CH_2$–$CF_2$–C$\underline{F}_2$–C$\underline{F}_2$–C$\underline{F}_2$–C$\underline{F}_2$) -121.64 (m, 2F, $CF_3$–$CF_2$–$CF_2$–$CF_2$–C$\underline{F}_2$), -122.46 (m, 2F, $CF_3$–C$\underline{F}_2$), -123.15 (m, 2F, $CF_3$–$CF_2$–C$\underline{F}_2$), -125.72 (m, 2F, $CF_3$–$CF_2$–$CF_2$–C$\underline{F}_2$).
**ESI MS**: *m/z* found 851.08 [M−Cl]$^+$, calcd 851.07.

## III. X-Ray crystallography

Bragg-intensities of **2d**-**2f** and **3e** were collected at low temperature using Cu*Kα* radiation on a Rigaku SuperNova dual system diffractometer with an Atlas CCD detector. The datasets were reduced and corrected for absorption, with the help of a set of faces enclosing the crystal as snugly as possible, with the latest available version of *CrysAlis^Pro^*.[2] The solutions and refinements of the structures were performed by the latest available version of *ShelXT*[3] and *ShelXL*.[4] All non-hydrogen atoms were refined anisotropically using full-matrix least-squares based on $|F|^2$. The hydrogen atoms were placed at calculated positions by means of the "riding" model in which each H-atom was assigned a fixed isotropic displacement parameter with a value equal to 1.2 $U_{eq}$ of its parent C-atom (1.5 $U_{eq}$ for the methyl groups). Crystallographic and refinement data are summarized in Table S1. Crystallographic data have been deposited with the Cambridge Crystallographic Data Centre and correspond to the following codes: **2d** (2335488), **2e** (2335489), **2f** (2335490), and **3e** (2335491). These data can be obtained free of charge via www.ccdc.cam.ac.uk/data_request/cif, or by emailing data_request@ccdc.cam.ac.uk, or by contacting The Cambridge Crystallographic Data Centre, 12 Union Road, Cambridge CB2 1EZ, UK; fax: +44 1223 336033.

**Table S1.** X-Ray crystallography data of the single crystals of **2d**-**2f** and **3e**.

| **Compound** | **2d** | **2e** | **2f** | **3e** |
|---|---|---|---|---|
| Formula | $C_{26}H_{29}Cl_2F_{13}N_2O_4$ | $C_{28}H_{29}Cl_2F_{17}N_2O_4$ | $C_{30}H_{29}Cl_2F_{21}N_2O_4$ | $C_{25}H_{25}Cl_3F_{17}N_3O_2$ |
| $D_{calc.}$/g cm$^{-3}$ | 1.557 | 1.638 | 1.706 | 1.684 |
| $\mu$/mm$^{-1}$ | 2.855 | 2.901 | 2.938 | 3.741 |
| Formula Weight | 751.41 | 851.43 | 951.45 | 828.83 |
| Colour | colourless | colourless | colourless | colourless |
| Shape | plate | plate | plate | needle |
| Size/mm$^3$ | 0.68×0.10×0.03 | 0.50×0.13×0.03 | 0.39×0.13×0.04 | 0.72×0.07×0.03 |
| $T$/K | 140.01(10) | 140.00(10) | 140.00(10) | 99.99(10) |
| Crystal System | orthorhombic | orthorhombic | orthorhombic | monoclinic |
| Flack Parameter | -0.003(11) | -0.01(2) | 0.27(8) | -0.01(8) |
| Hooft Parameter | 0.006(6) | -0.052(14) | -0.30(2) | -0.192(19) |
| Space Group | $Pca2_1$ | $Pca2_1$ | $Pca2_1$ | $I2$ |
| $a$/Å | 10.97900(20) | 11.0351(3) | 10.9654(12) | 22.4357(14) |
| $b$/Å | 30.0354(5) | 32.0681(9) | 34.582(6) | 5.2436(3) |
| $c$/Å | 9.7195(2) | 9.7587(2) | 9.7715(13) | 27.9895(18) |
| $\alpha$/° | 90 | 90 | 90 | 90 |
| $\beta$/° | 90 | 90 | 90 | 96.882(6) |
| $\gamma$/° | 90 | 90 | 90 | 90 |
| V/Å$^3$ | 3205.10(11) | 3453.38(16) | 3705.4(9) | 3269.1(3) |
| $Z$ | 4 | 4 | 4 | 4 |
| $Z'$ | 1 | 1 | 1 | 1 |
| Wavelength/Å | 1.54184 | 1.54184 | 1.54184 | 1.54184 |
| Radiation type | CuK-$\alpha$ | CuK-$\alpha$ | Cu$K$-$\alpha$ | CuK-$\alpha$ |
| $\theta_{min}$/° | 4.288 | 4.136 | 3.835 | 4.946 |
| $\theta_{max}$/° | 75.376 | 75.428 | 76.510 | 65.070 |
| Measured Refl. | 57908 | 23896 | 19475 | 5297 |
| Independent Refl. | 6448 | 5468 | 19475 | 5297 |
| Reflections with I > 2σ(I) | 5851 | 5000 | 8863 | 3614 |
| $R_{int}$ | 0.0538 | 0.0463 | n/a | n/a |
| Parameters | 431 | 485 | 566 | 454 |
| Restraints | 1 | 4 | 913 | 562 |
| Largest Peak/e Å$^{-3}$ | 0.313 | 0.543 | 1.447 | 1.630 |
| Deepest Hole/e Å$^{-3}$ | -0.250 | -0.279 | -0.823 | -1.471 |
| GooF | 1.026 | 1.040 | 1.212 | 1.830 |
| $wR_2$ (all data) | 0.0961 | 0.1062 | 0.4328 | 0.4649 |
| $wR_2$ | 0.0912 | 0.1015 | 0.3789 | 0.4322 |
| $R_1$ (all data) | 0.0426 | 0.0443 | 0.2309 | 0.2102 |
| $R_1$ | 0.0370 | 0.0391 | 0.1547 | 0.1769 |
| **CCDC number** | **2335488** | **2335489** | **2335490** | **2335491** |

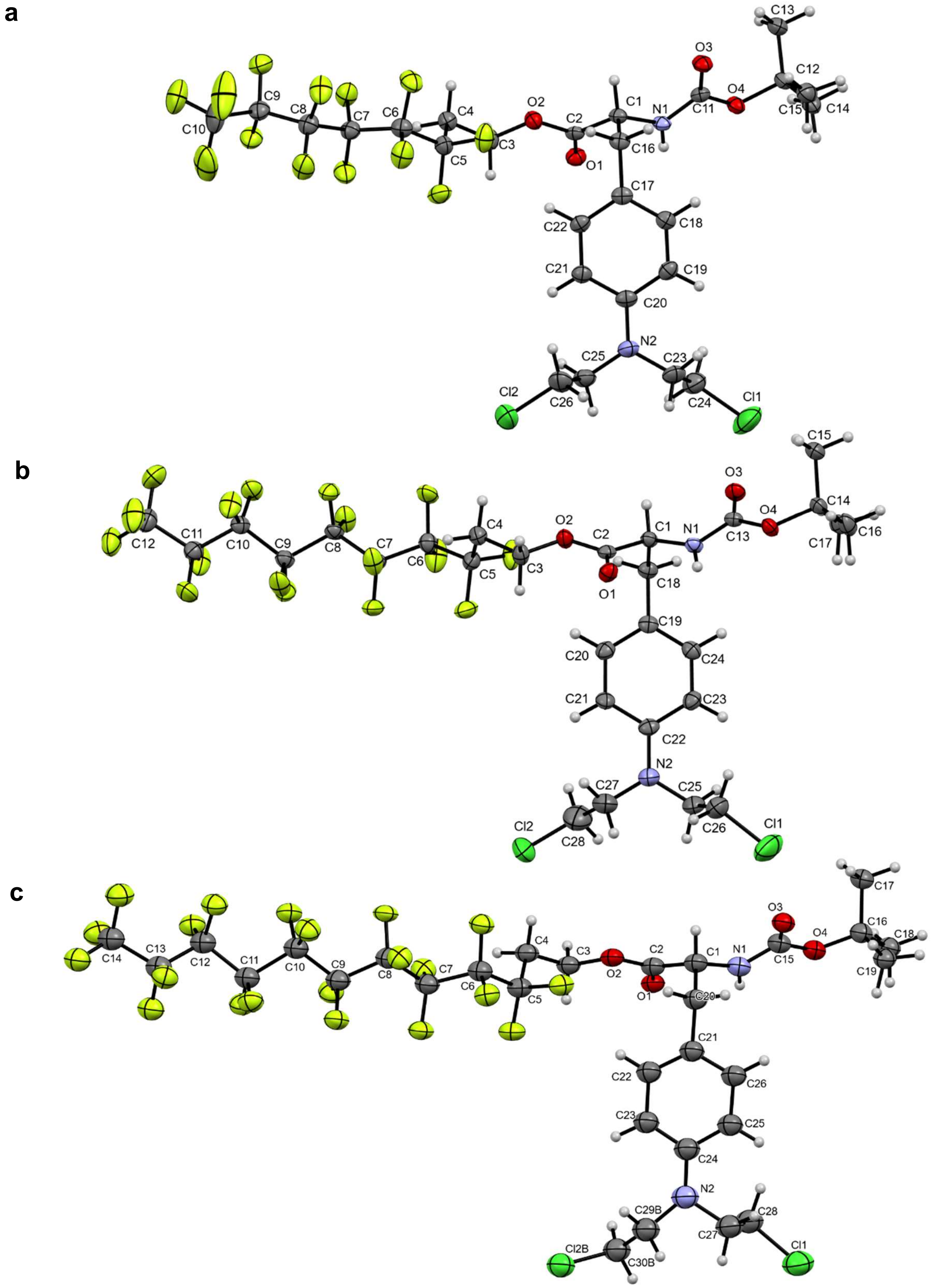


**Figure S3.** X-Ray single crystal structures of **a**, **2d**; **b**, **2e**; and **c**, **2f**. Selected bond lengths (Å) for **2d**: Cl1–C24, 1.791(4); Cl2–C26, 1.785(4); N2–C20, 1.387(4); N1–C1, 1.448(4); O1–C2, 1.206(4); O2–C2, 1.348(4); C–$F_{avg}$, 1.338(4). **2e**: Cl1–C26, 1.789(4); Cl2–C28, 1.815(6); N2–C22, 1.388(5); N1–C1, 1.444(4); O1–C2, 1.204(4); O2–C2, 1.349(4); C–$F_{avg}$, 1.339(5). **2f**: Cl1–C28, 1.80(3); Cl2–C30, 1.80(2); N2–C24, 1.36(5); N1–C1, 1.39(3); O1–C2, 1.26(4); O2–C2, 1.31(3); C–$F_{avg}$, 1.33(4).

## IV. Cytotoxicity studies

### 1. Cell culture

The human retina (RPE1) and retinoblastoma (Y79) cell lines were purchased from ATCC, Switzerland. RPMI 1640, DMEM high glucose Glutamax and DMEM GlutaMAX media were obtained from Life Technologies, and fetal bovine serum (FBS) was obtained from Sigma. Cell culture was performed in RPMI 1640 with 20% FBS and DMEM high glucose Glutamax with 10% FBS for Y79 and RPE1, respectively. Cell passage was performed twice per week using the appropriate medium and the resulting flaks were maintained in a bioincubator (Thermo Scientific, Switzerland) that controlled the humidity and fixed the CO2 rate to 5%. Cell viability tests were conducted in a flat bottomed 96 or 384 well plate using a compound stock solution prepared in dimethyl sulfoxide (DMSO) for all compounds except Cisplatin. The maximum concentration of DMSO in the wells was 0.1% DMSO.

### 2. Presto Blue cell viability assay in Y79 after 24 hours

The optimization of the starting concentration was carried out for each tested compound after 24 h of incubation. In order to conduct the tests at the mentioned time lapses, Y79's work cell solutions were prepared at $10^6$ cell/mL for the 24 h experiment. The same protocol as described below was used, however in this case, the experiment was conducted manually.

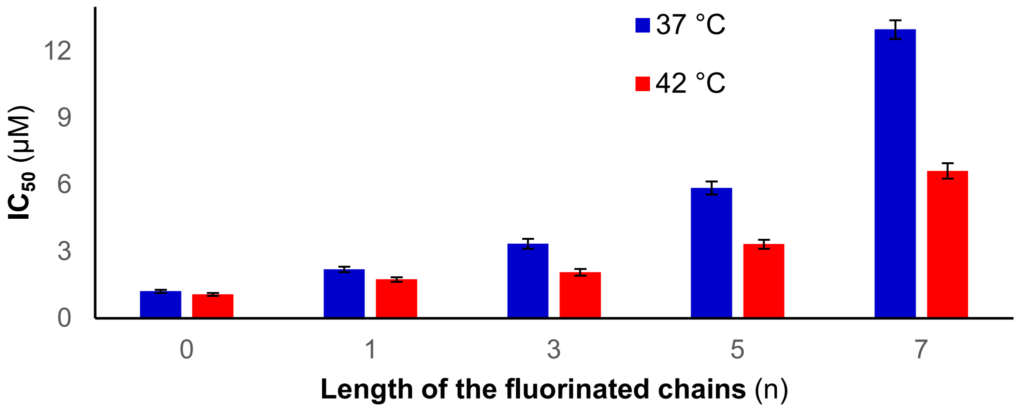


**Figure S4.** Comparison between $IC_{50}$ values of **3a-3f** at 37 °C and 42 °C in Y79 after 24 h of incubation.

### 3. Presto Blue cell viability assay in Y79 and RPE1 cells at 72 h

In order to evaluate the cytotoxicity of **3a-3f** on Y79 and RPE1 cell lines, cell viability tests were conducted using cell work solutions at $4x10^5$ and $7x10^5$ cell/mL respectively and compound stock solutions were prepared at 10 µM in DMSO. The robotic System (Echo 550

LIQUID HANDLER) charged the 384-cell plate with a decreasing volume of each compound's SS in order to have a concentration gradient that covers 100 to 0.7 µM. DMSO was added to each well so as to have the same final volume of compound's solution in each well. Cells were added in the plate using a disperser (Thermo Scientific Multidrop Combi, Switzerland).

For thermal assays, the plates were placed in a heat chamber at 42 °C for 1 h and then transferred to a bioincubator at 37 °C for 71 h where the control plates were already placed. After 72 h of a total incubation time, 10 mL of presto blue were added to each well and all the plates were incubated for 1 h at 37 °C. For presto blue cell viability assays, gambogic acid was used at 10 µM and represented the positive control, while DMSO at a final concentration of 0.1% presented the negative control. Fluorescence was measured using a plate reader (Tecan INFINITE F500 reader, Switzerland) at 560 nm for absorbance and 590 nm for fluorescence. The normalization of the fluorescence's intensities was conducted with GraphPad software using Gambogic acid and DMSO as positive and negative control, respectively.

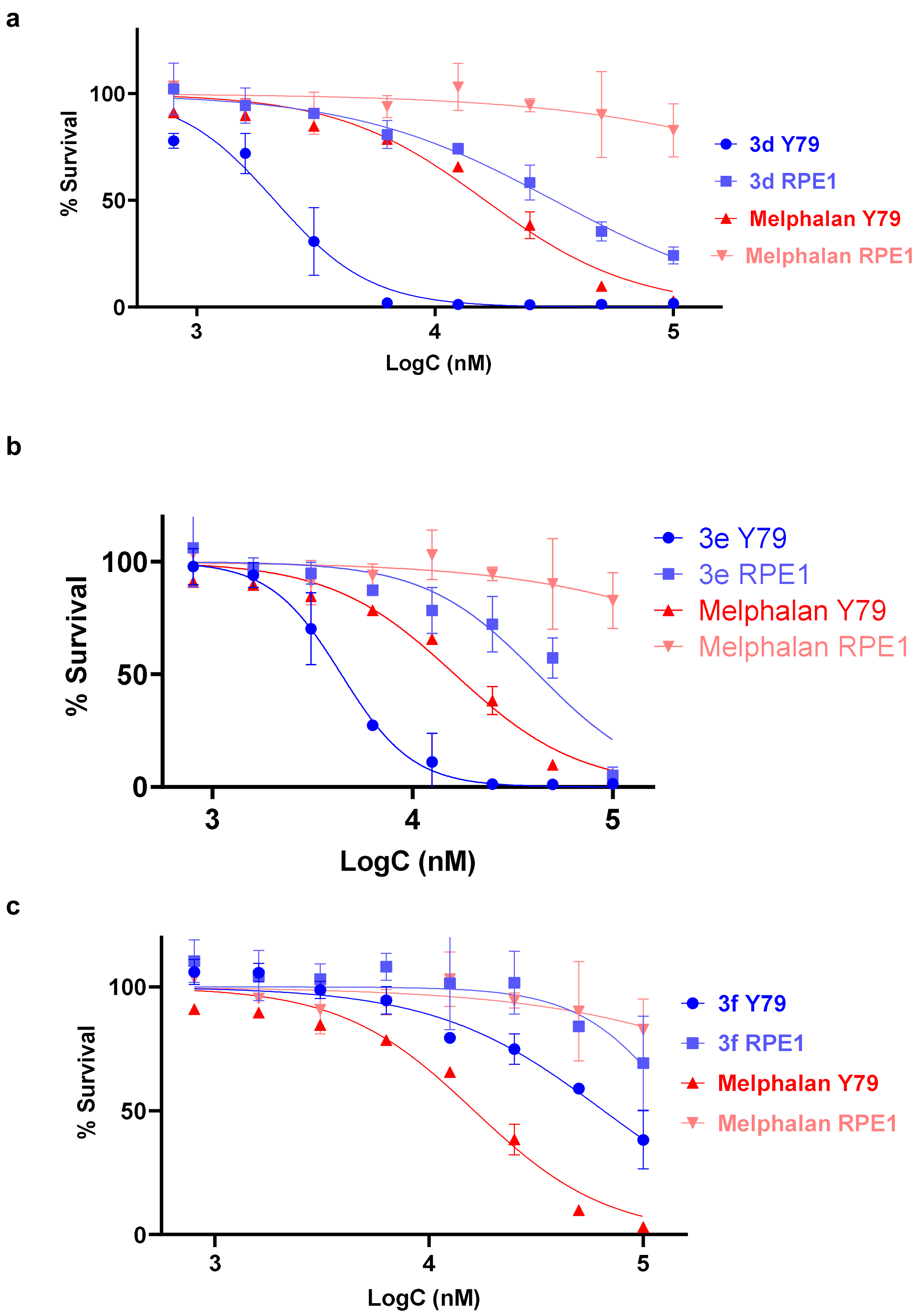


**Figure S5.** Dose response curves of **3d-3f** (Panel A-C) at 42 °C on Y79 and RPE1 cell lines after 72 h of incubation. The error bars indicate the standard deviation observed for three measurements of 1 concentration in 1 experiment (N = 1).

## V. DNA binding ESI-MS study

### Samples preparation and measurements' conditions for MS analysis

For the following experiments, samples were prepared in MilliQ water (EPFL) and the concentration of the dsDNA oligonucleotide sequence AGGCAG (Bioserch technology, USA) was fixed at $10^{-4}$ M.

After conducting the optimization of the conditions of the experiment, melphalan and **3a**-**3f** were incubated at 37 °C with the oligonucleotide sequence at a 1:5 ratio for 48 h in a Bioshaker (ThermoMixer C, Vaudaux Eppendorf). 400 µL of each mixture were prepared in an Eppendorf tube and 100 µL were sampled at 24 h, 48 h, 72 h and 1 week of incubation.

The resulting adducts were characterized by Mass spectrometry. The analyses were performed on an LTQ Orbitrap FTMS instrument (LTQ Orbitrap Elite FTMS, Thermo Scientific, Bremen, Germany) operated in the negative mode coupled to HESI-II probe in an Ion Max ion Source. The samples were injected within an infusion rate of 10 µL/min. The experimental condition for the ionization voltage was -1.2kV whereas the temperature of ion transfer capillary was 120 °C. FTMS spectra were obtained using the high mass range between 200-3000 *m/z* range in the reduced profile mode with a resolution set to 120K. In all spectra 10 microscan were acquired with a maximum injection time value of 1000 ms. The data analysis was carried out using cheminfo software and XCalibure for the monoIsotopic mass comparison.

**Table S2.** Adducts of melphalan, **3a-3e** with dsDNA after 48 h incubation at 37 °C.

| Adducts | Molecular formula | Theoretical m/z | Experimental m/z |
|---|---|---|---|
| [ *B1* + *melphalan* – Cl ]$^{2-}$ | $C_{72}H_{86}N_{30}O_{34}P_5ClNa_2$ | 1075.2053 | 1075.2227 |
| [ *B2* + *melphalan* − Cl ]$^{2-}$ | $C_{70}H_{88}N_{20}O_{38}P_5ClNa_2$ | 1026.1876 | 1026.2059 |
| [ *B1* + **3a** − Cl ]$^{2-}$ | $C_{75}H_{94}N_{30}O_{34}P_5ClF_3$ | 1102.7433 | 1102.7432 |
| [ *B2* + **3a** − Cl ]$^{2-}$ | $C_{73}H_{93}N_{20}O_{38}P_5ClF_3$ | 1052.2139 | 1052.2242 |
| [ *B1* + **3a** − 2Cl + OH ]$^{2-}$ | $C_{75}H_{92}N_{30}O_{35}P_5F_3$ | 1092.2485 | 1092.2595 |
| [ *B2* + **3a** − 2Cl + OH ]$^{2-}$ | $C_{73}H_{94}N_{20}O_{39}P_5F_3$ | 1043.2308 | 1043.2419 |
| [ *B1* + **3b** − Cl ]$^{2-}$ | $C_{76}H_{91}N_{30}O_{34}P_5ClF_5$ | 1126.2300 | 1226.2388 |
| [ *B2* + **3b** − Cl ]$^{2-}$ | $C_{74}H_{93}N_{20}O_{38}P_5ClF_5$ | 1077.2123 | 1077.2211 |
| [ *B1* + **3b** − 2Cl + OH ]$^{2-}$ | $C_{76}H_{92}N_{30}O_{35}P_5F_5$ | 1117.2469 | 1117.2577 |
| [ *B2* + **3b** − 2Cl + OH ]$^{2-}$ | $C_{74}H_{94}N_{20}O_{39}P_5F_5$ | 1068.2292 | 1068.2399 |
| [ *B1* + **3c** − Cl ]$^{2-}$ | $C_{78}H_{91}N_{30}O_{34}P_5ClF_9$ | 1176.2268 | 1176.2368 |
| [ *B2* + **3c** − Cl ]$^{2-}$ | $C_{76}H_{94}N_{20}O_{38}P_5ClF_9$ | 1127.2091 | 1127.2184 |
| [ *B1* + **3c** − 2Cl + OH ]$^{2-}$ | $C_{78}H_{92}N_{30}O_{39}P_5F_5$ | 1167.2437 | 1167.2546 |
| [ *B2* + **3c** − 2Cl + OH ]$^{2-}$ | $C_{76}H_{94}N_{20}O_{39}P_5F_9$ | 1118.2260 | 1118.2367 |
| [ *B1* + **3c** – Cl – $CH_2CH_2(CF_2)_3CF_3$ ]$^{2-}$ | $C_{72}H_{85}N_{30}O_{34}P_5ClNa_2$ | 1074.7014 | 1074.7055 |
| [ *B2* + **3c** − Cl – $CH_2CH_2(CF_2)_3CF_3$ ]$^{2-}$ | $C_{70}H_{92}N_{20}O_{38}P_5ClNa_2$ | 1025.6837 | 1025.6886 |
| [ *B1* + **3d** − Cl ]$^{2-}$ | $C_{80}H_{91}N_{30}O_{34}P_5ClF_{13}$ | 1226.2236 | 1226.2247 |
| [ *B2* + **3d** − Cl ]$^{2-}$ | $C_{78}H_{93}N_{20}O_{38}P_5ClF_{13}$ | 1177.2059 | 1177.2092 |
| [ *B1* + **3d** – Cl – $CH_2CH_2(CF_2)_5CF_3$ ]$^{2-}$ | $C_{72}H_{85}N_{30}O_{34}P_5ClNa_2$ | 1074.7014 | 1074.7086 |
| [ *B2* + **3d** − Cl – $CH_2CH_2(CF_2)_5CF_3$ ]$^{2-}$ | $C_{70}H_{92}N_{20}O_{38}P_5ClNa_2$ | 1025.6837 | 1025.6892 |
| [ *B1* + **3e** − Cl ]$^{2-}$ | $C_{82}H_{91}N_{30}O_{34}P_5ClF_{17}$ | 1276.2204 | 1276.2217 |
| [ *B2* + **3e** − Cl ]$^{2-}$ | $C_{80}H_{93}N_{20}O_{38}P_5ClF_{17}$ | 1227.2027 | 1227.2039 |
| [ *B1* + **3e** – Cl – $CH_2CH_2(CF_2)_7CF_3$ ]$^{2-}$ | $C_{72}H_{85}N_{30}O_{34}P_5ClNa_2$ | 1074.7014 | 1074.7059 |
| [ *B2* + **3e** − Cl – $CH_2CH_2(CF_2)_7CF_3$ ]$^{2-}$ | $C_{70}H_{92}N_{20}O_{38}P_5ClNa_2$ | 1025.6837 | 1025.6881 |

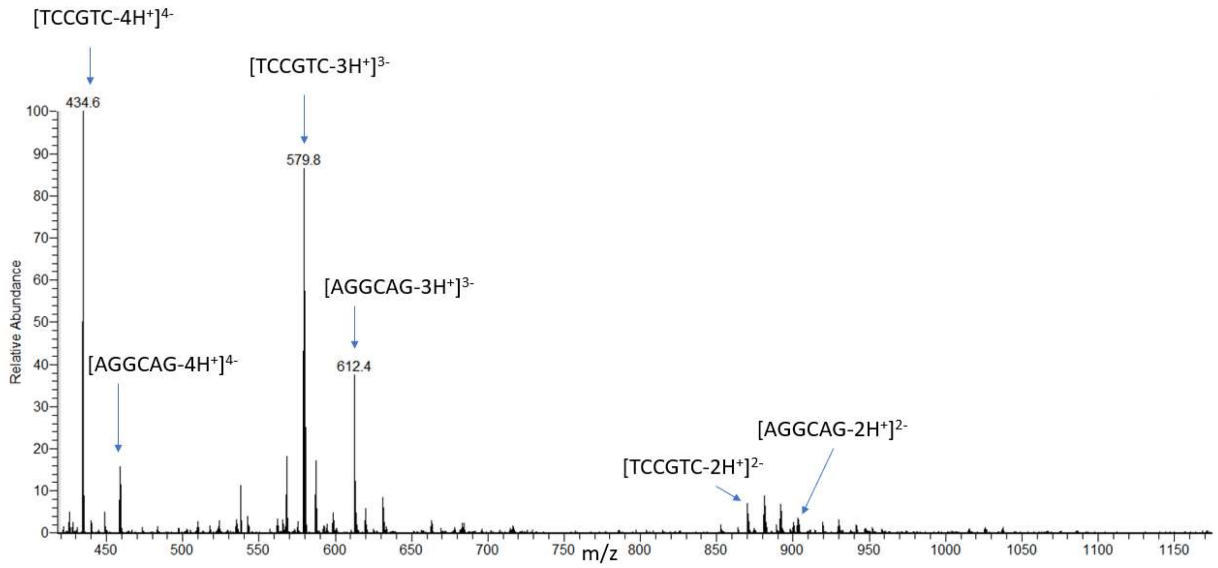


**Figure S6.** MS spectrum of the dsDNA oligonucleotide.

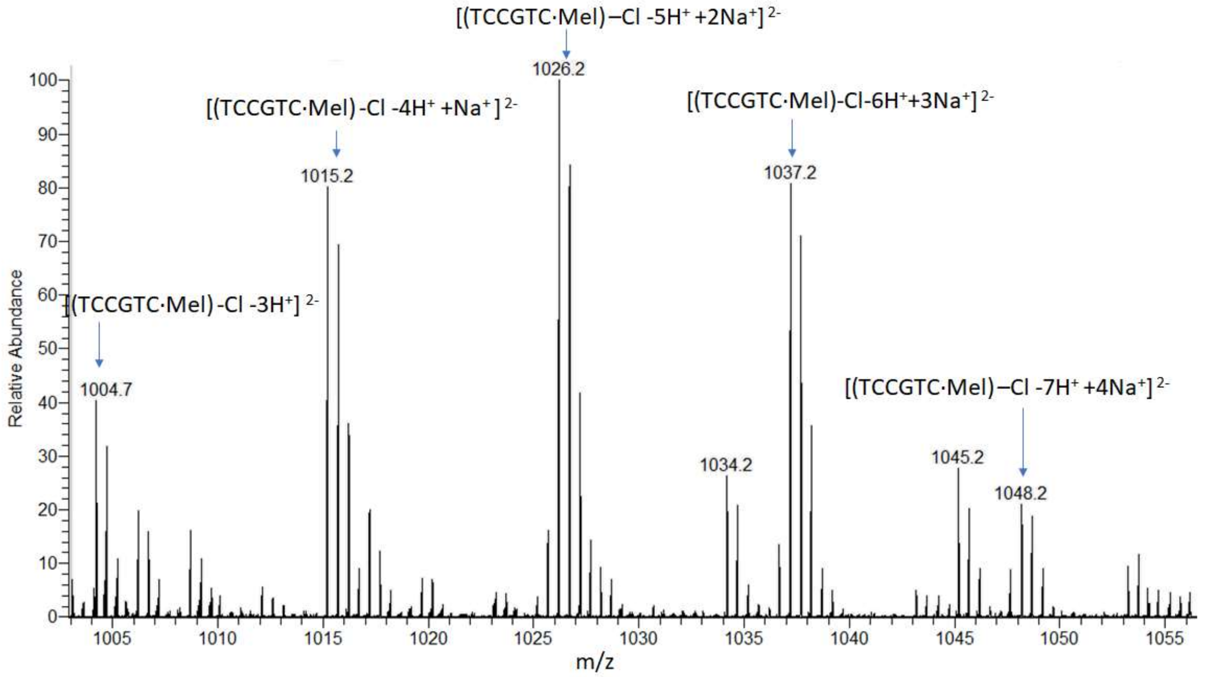


**Figure S7.** MS spectrum of the melphalan adducts with the TCCGTC sequence, after incubation for 48 h at 37 °C in a 1:5 ratio.

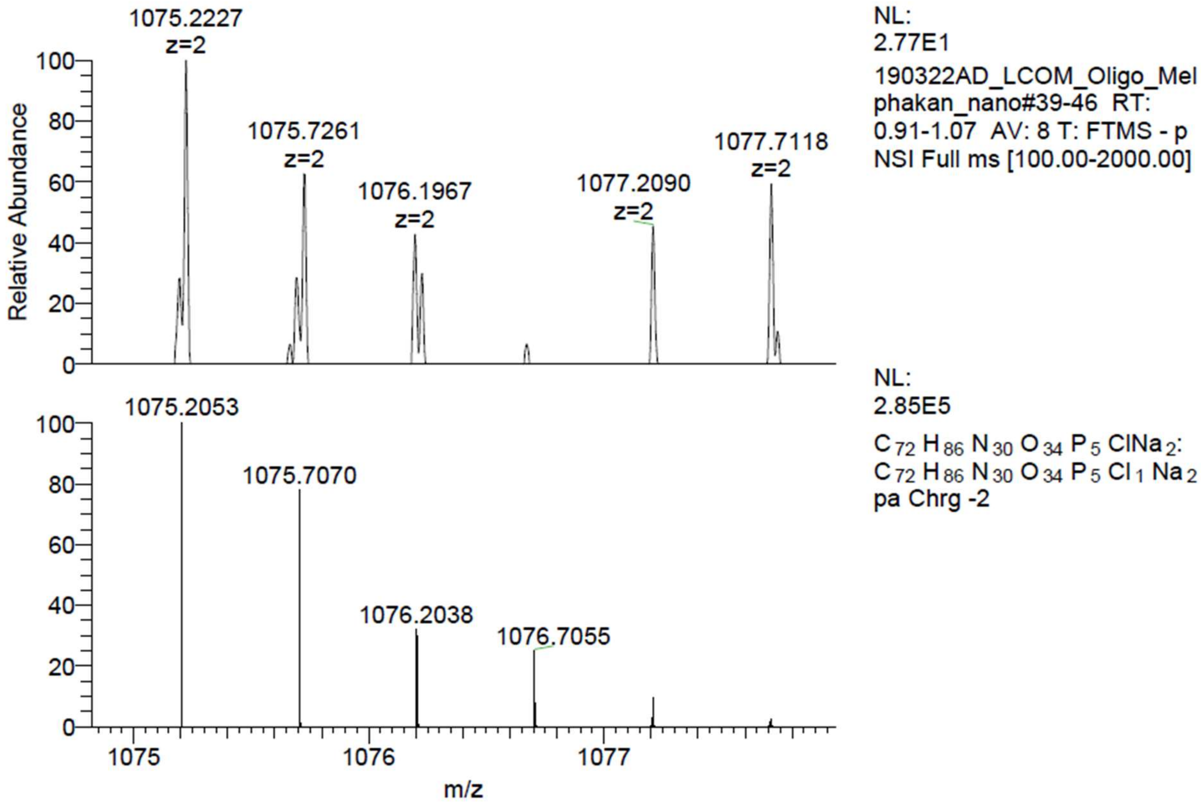


**Figure S8.** Isotopic profile of [*B1* + *melphalan* − Cl]$^{2-}$

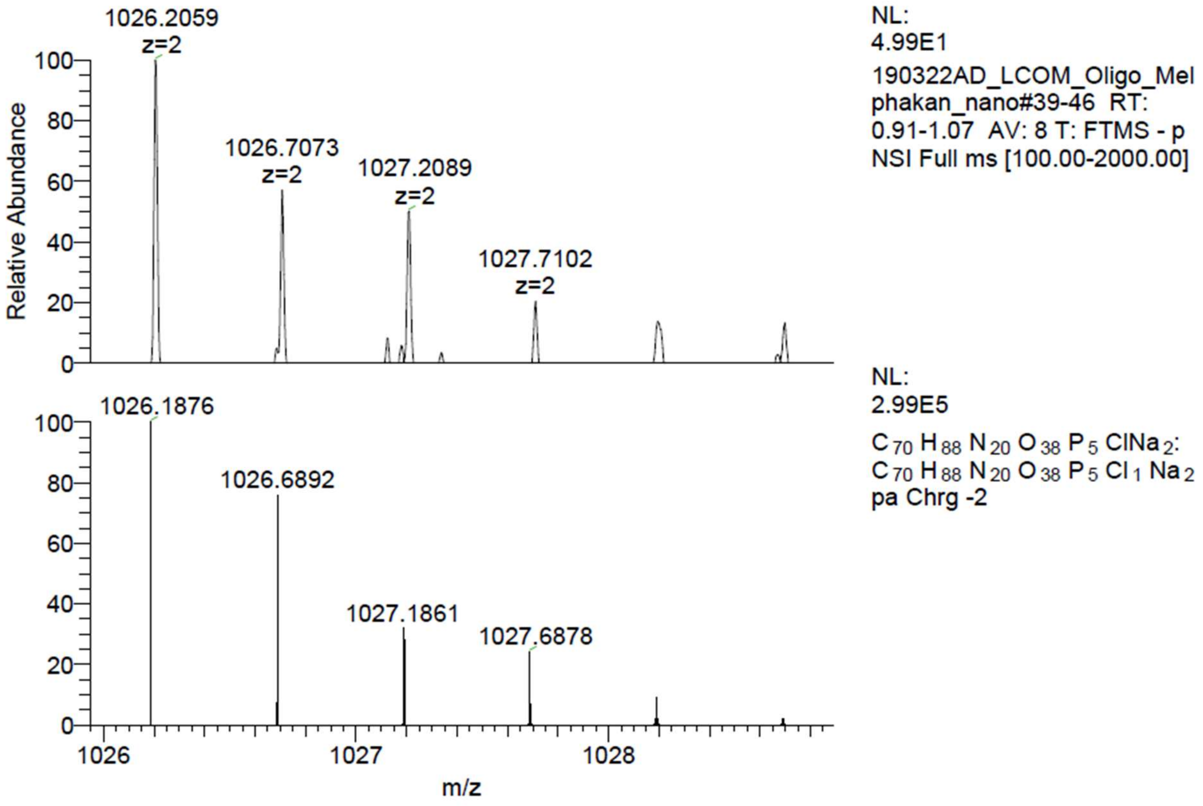


**Figure S9.** Isotopic profile of [*B2* + *melphalan* − Cl]$^{2-}$

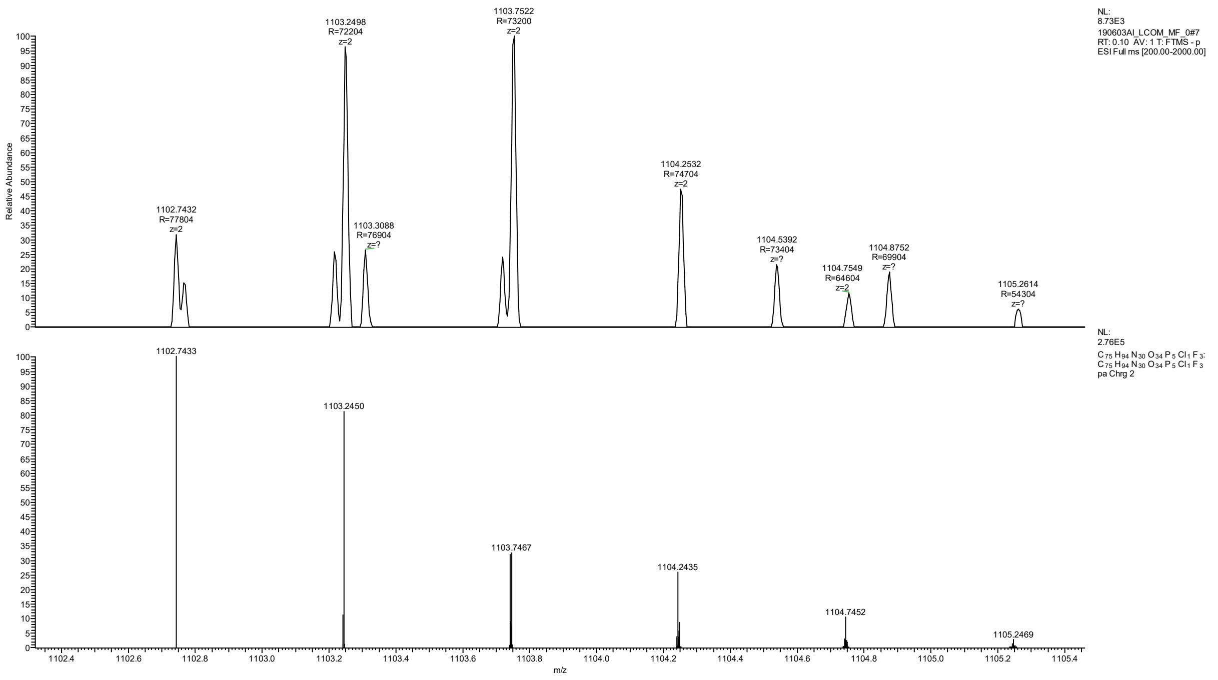


**Figure S10.** Isotopic profile of [*B1* + **3a** − Cl]$^{2-}$

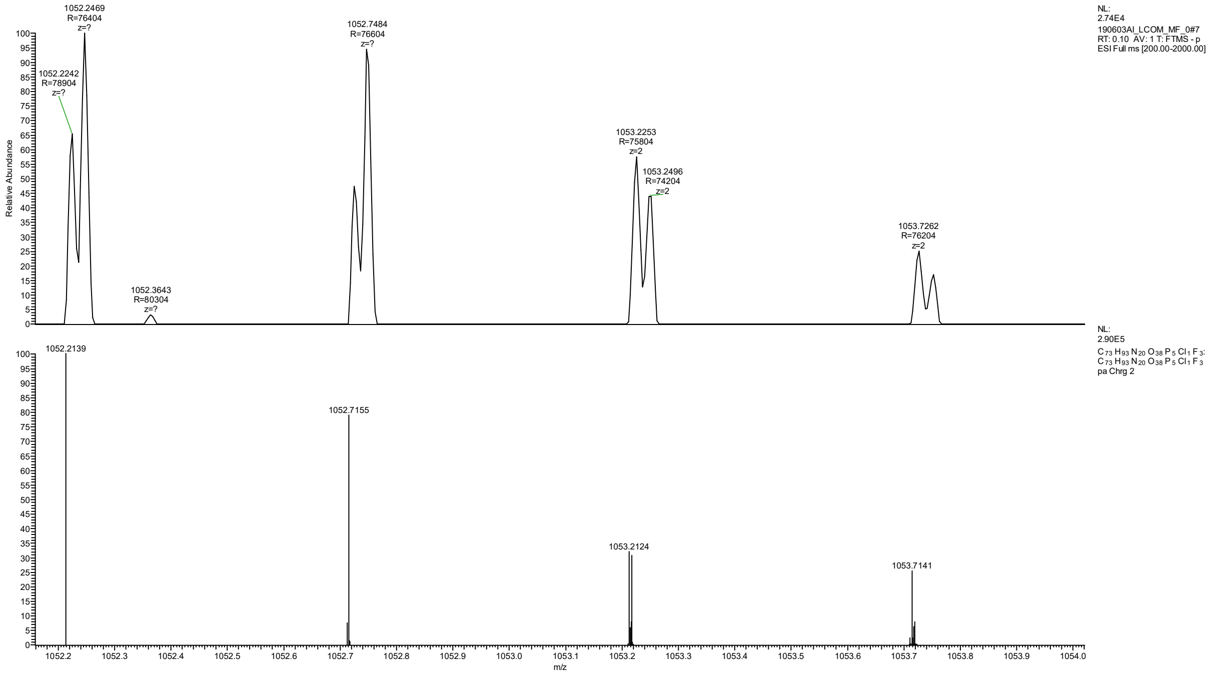


**Figure S11.** Isotopic profile of [*B2* + **3a** − Cl]$^{2-}$

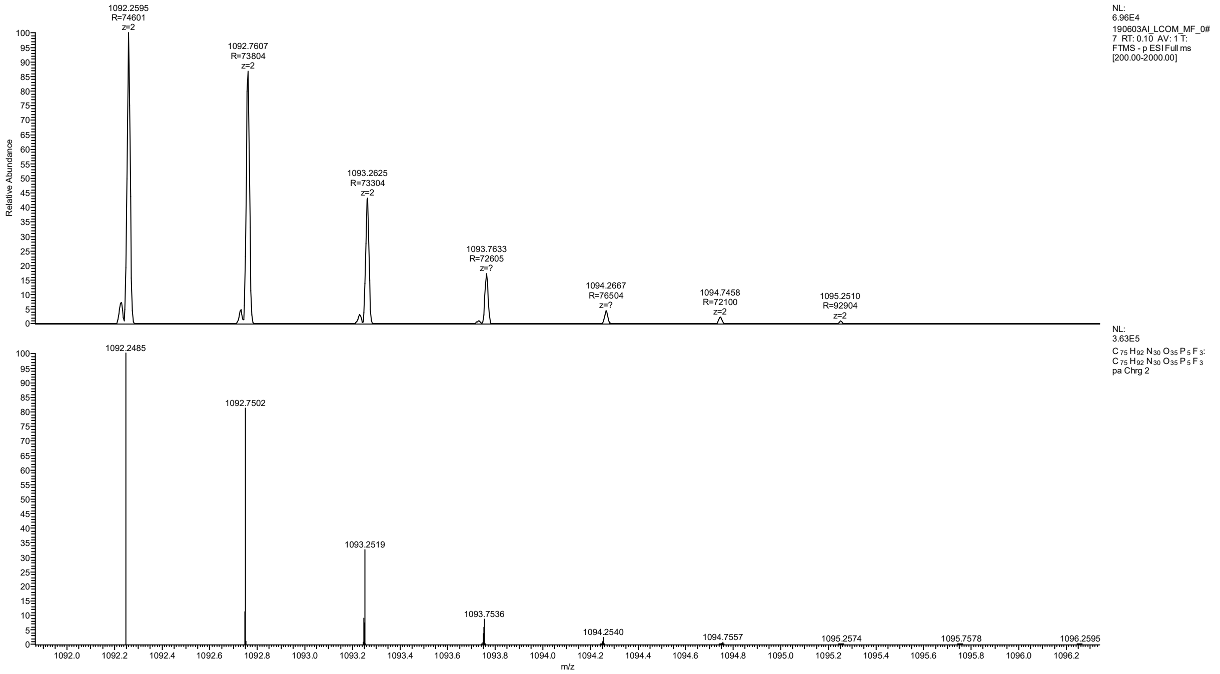


**Figure S12.** Isotopic profile of [*B1* + **3a** − 2Cl + OH]$^{2-}$

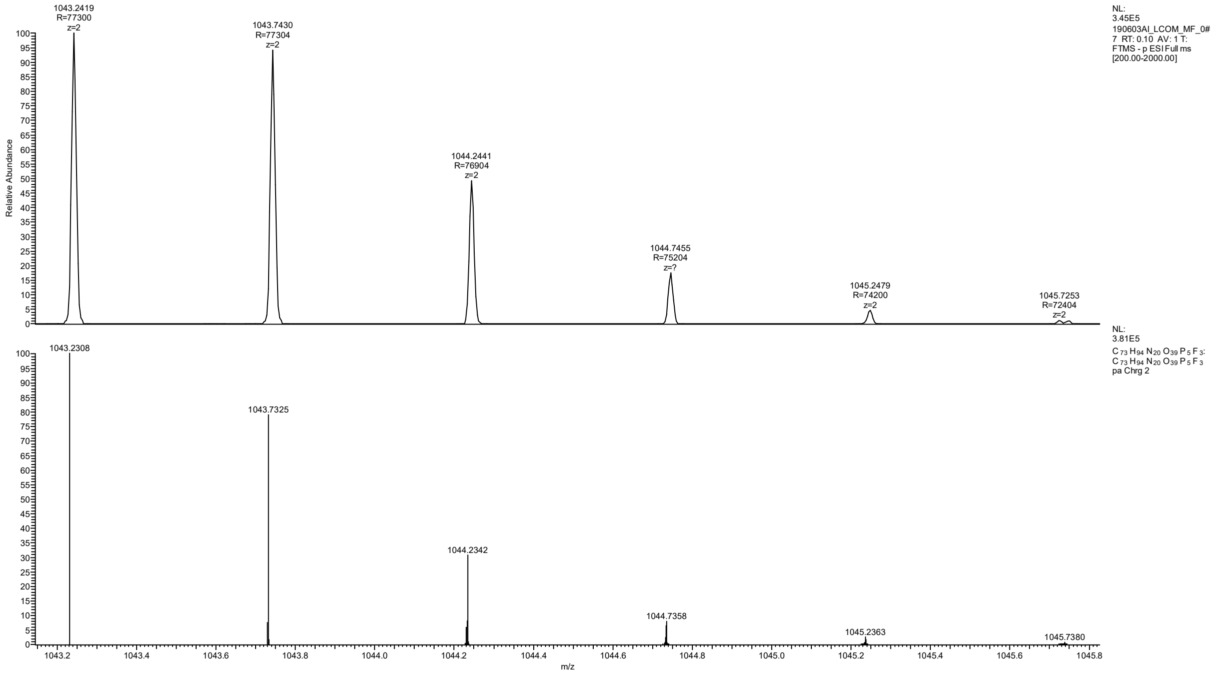


**Figure S13.** Isotopic profile of [*B2* + **3a** − 2Cl + OH]$^{2-}$

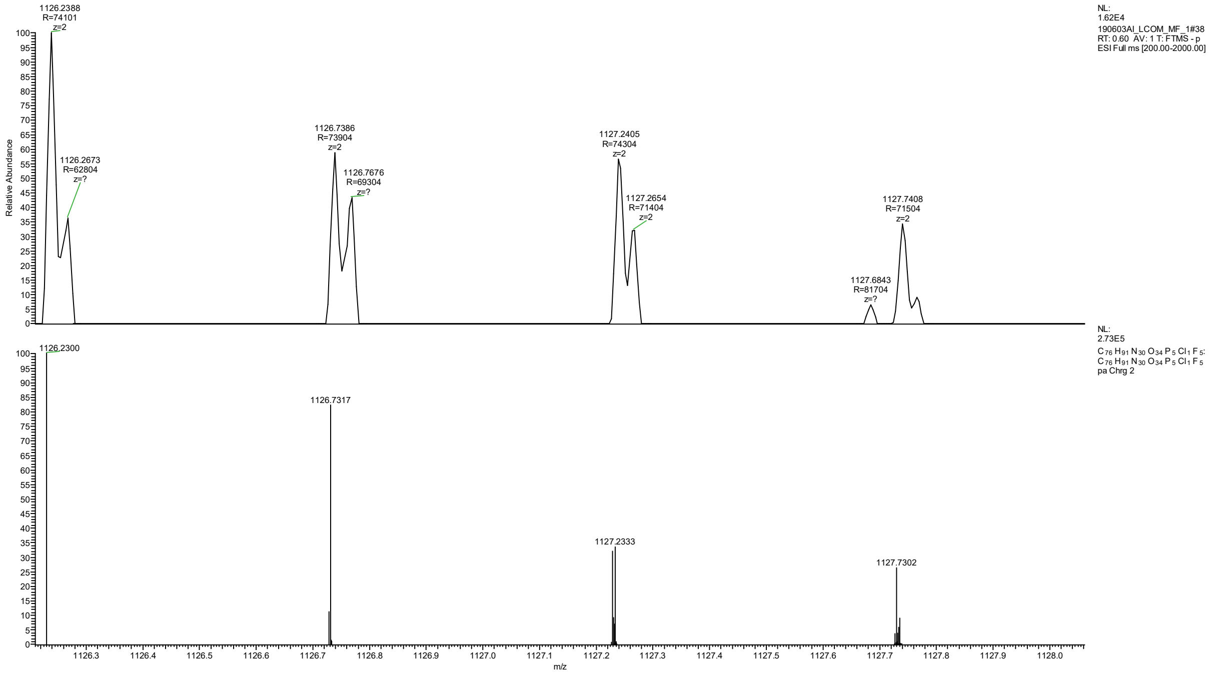


**Figure S14.** Isotopic profile of [*B1* + **3b** − Cl]$^{2-}$

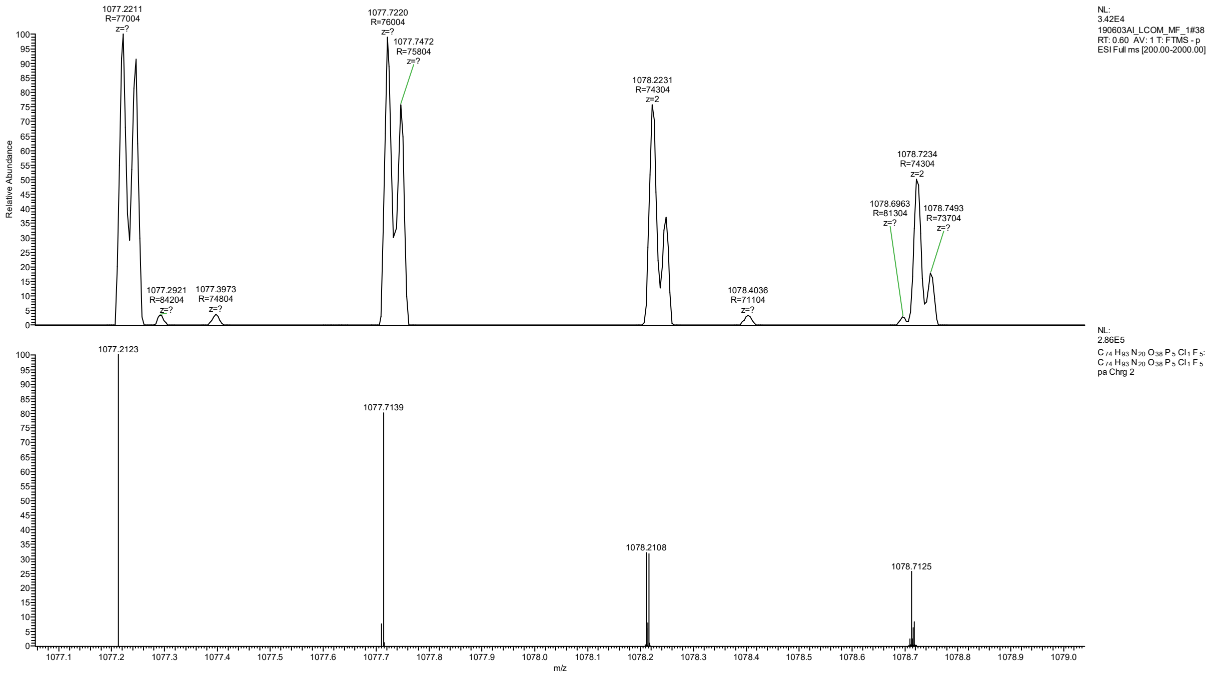


**Figure S15.** Isotopic profile of [*B2* + **3b** − Cl]$^{2-}$.

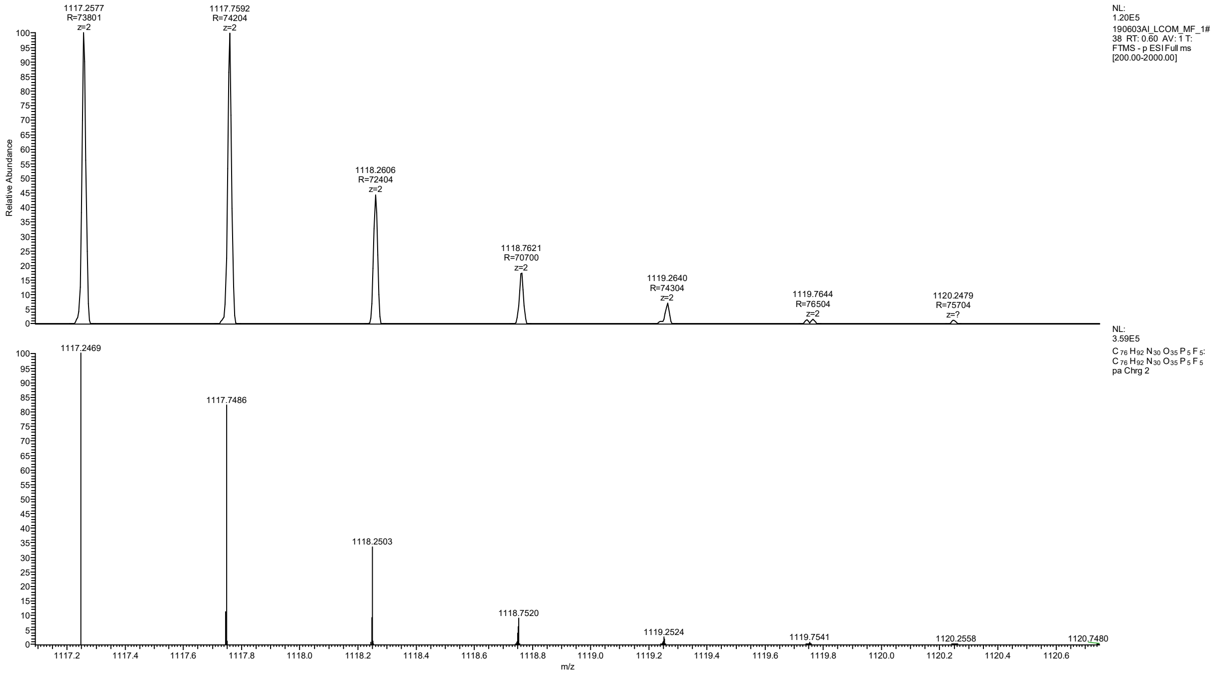


**Figure S16.** Isotopic profile of [*B1* + **3b** − 2Cl + OH]$^{2-}$

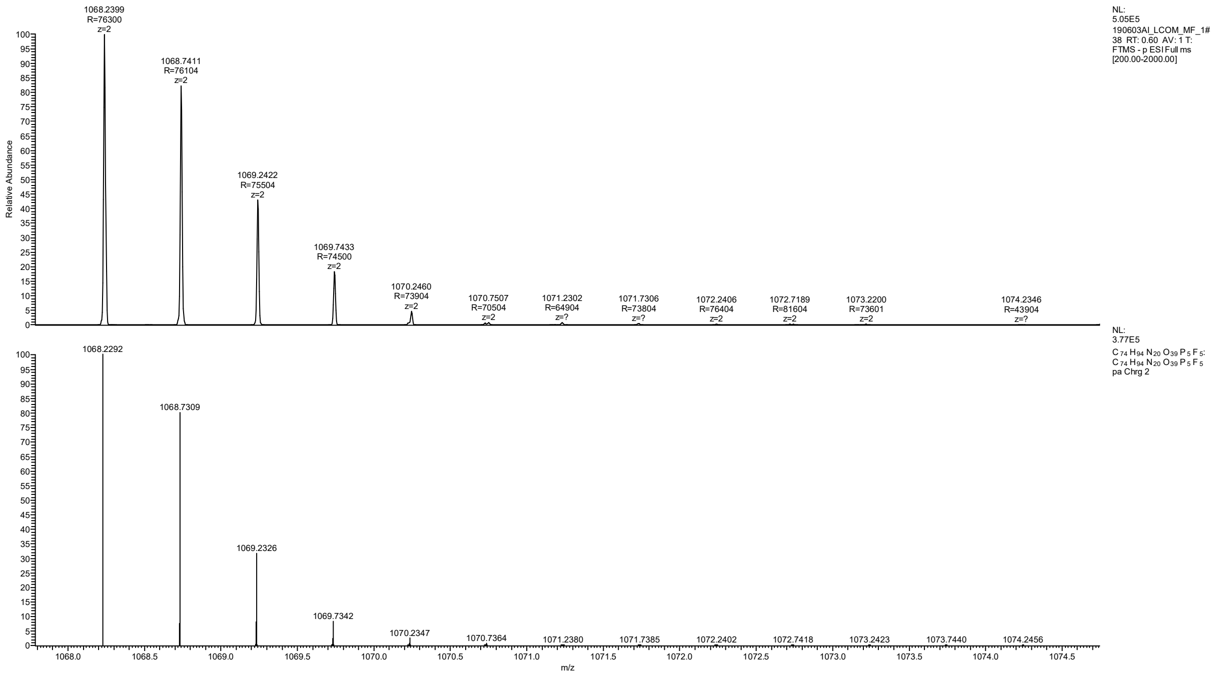


**Figure S17.** Isotopic profile of [*B2* + **3b** − 2Cl + OH]$^{2-}$

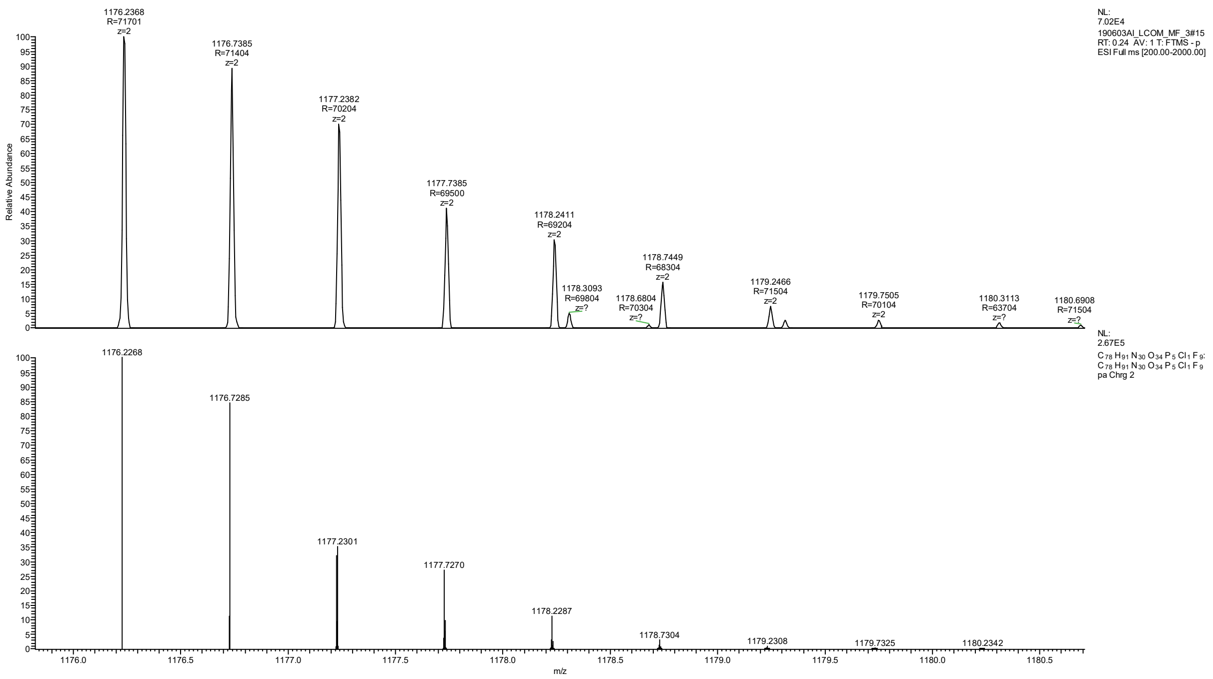

**Figure S18.** Isotopic profile of [*B1* + **3c** − Cl]$^{2-}$

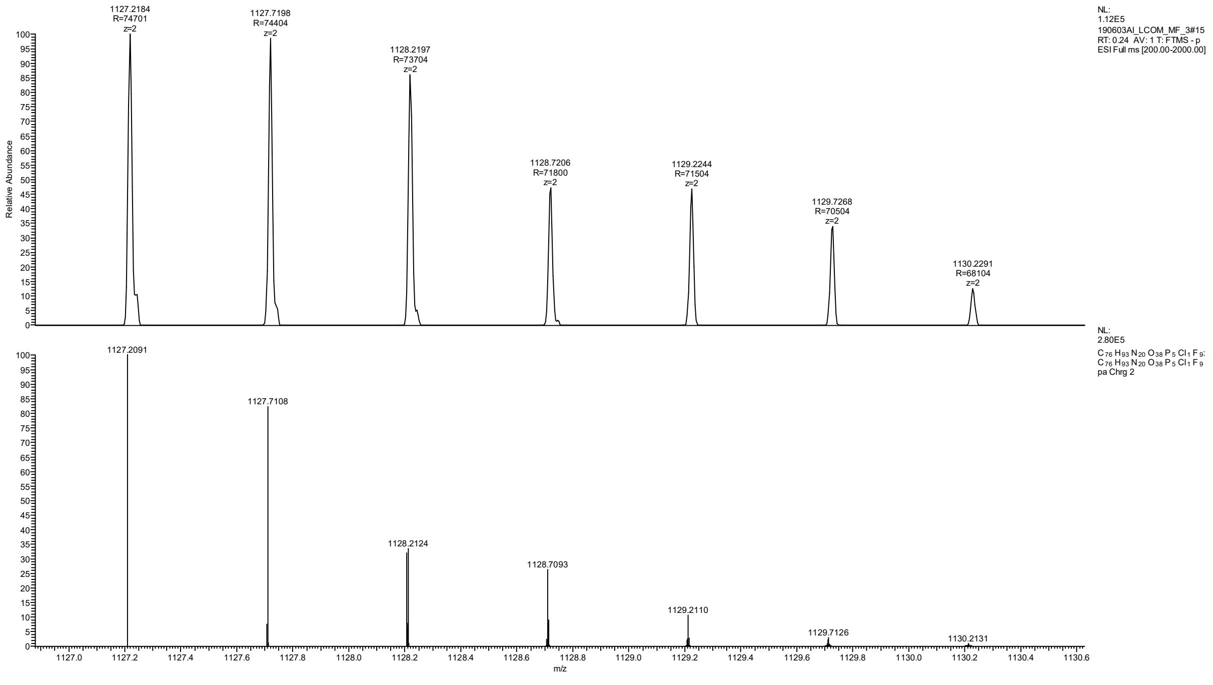

**Figure S19.** Isotopic profile of [*B2* + **3c** − Cl]$^{2-}$.

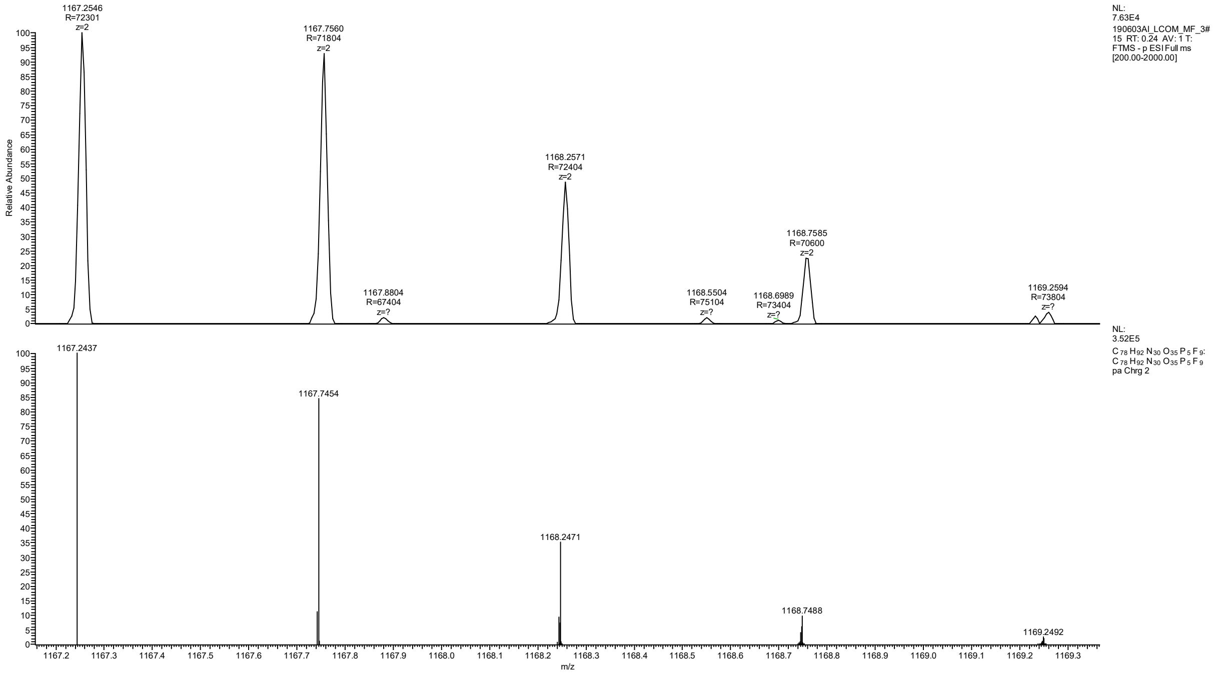


**Figure S20.** Isotopic profile of [*B1* + **3c** − 2Cl + OH]$^{2-}$

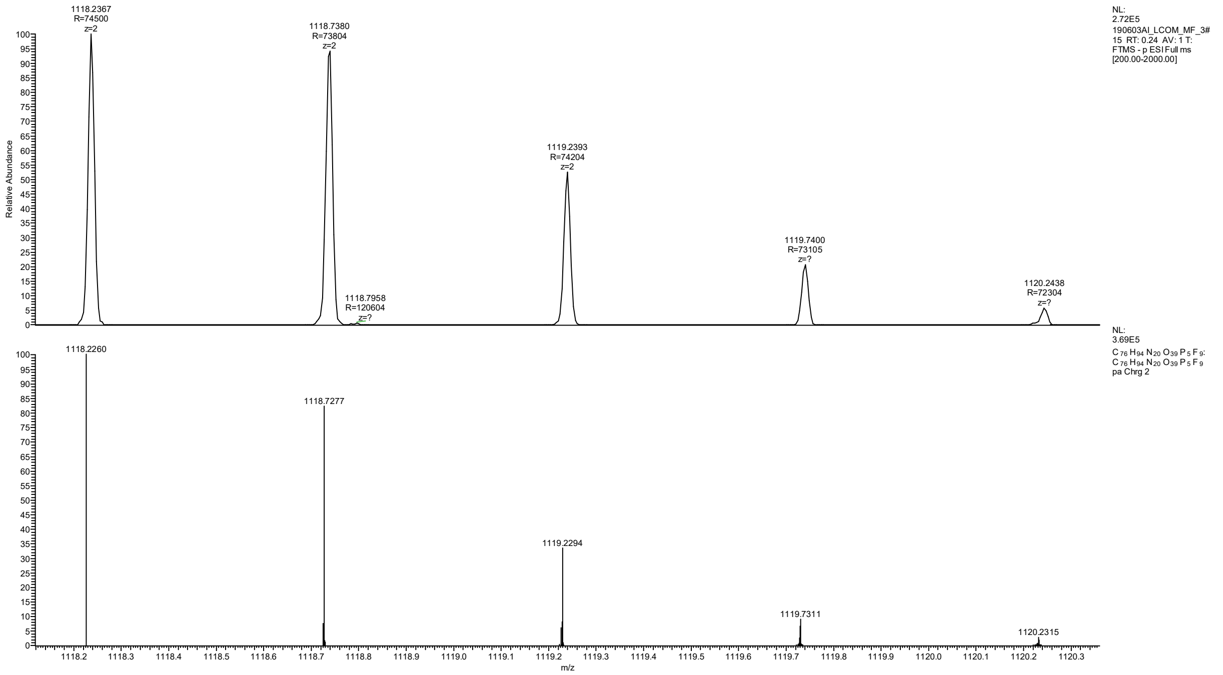


**Figure S21.** Isotopic profile of [*B2* + **3c** − 2Cl + OH]$^{2-}$

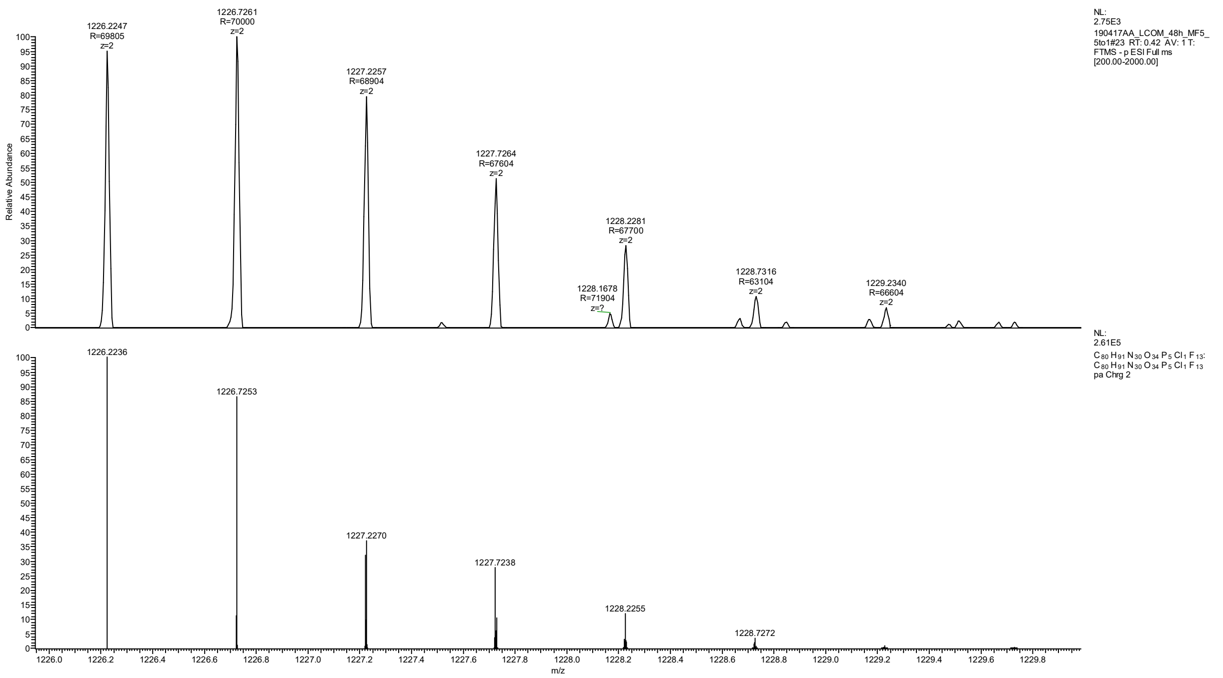


**Figure S22.** Isotopic profile of [*B1* + **3d** − Cl]$^{2-}$

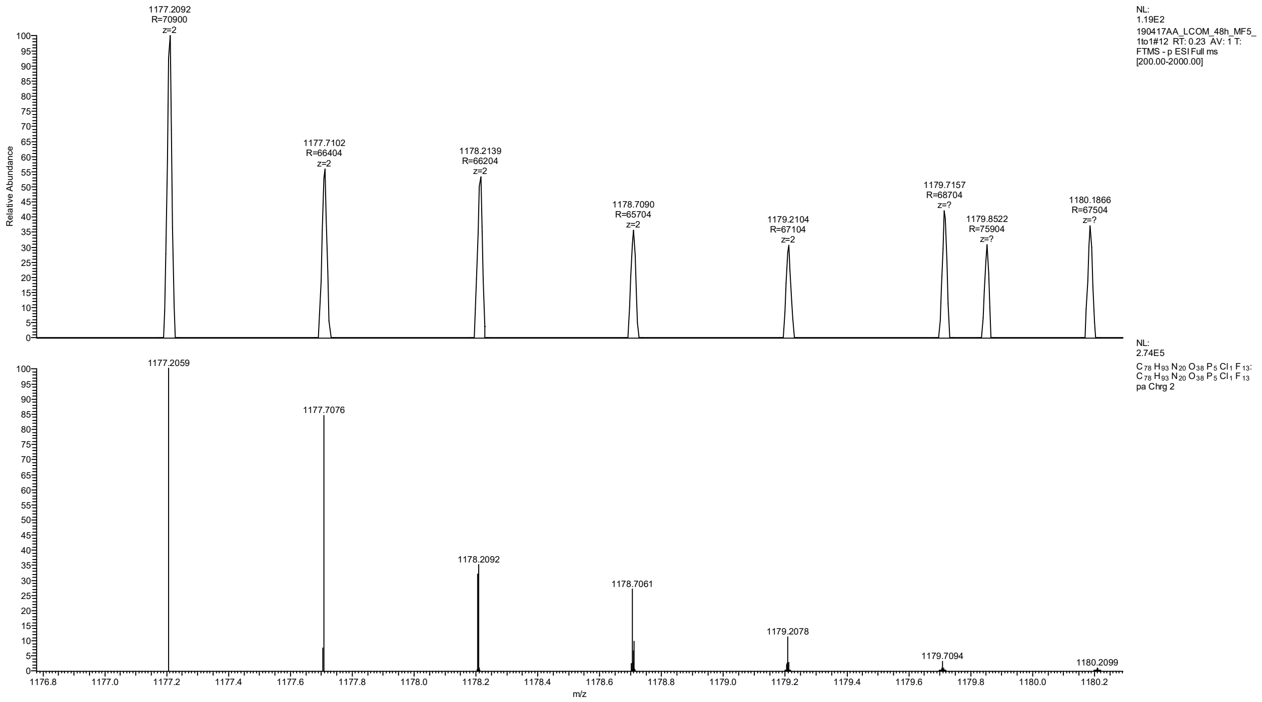


**Figure S23.** Isotopic profile of [*B2* + **3d** − Cl]$^{2-}$.

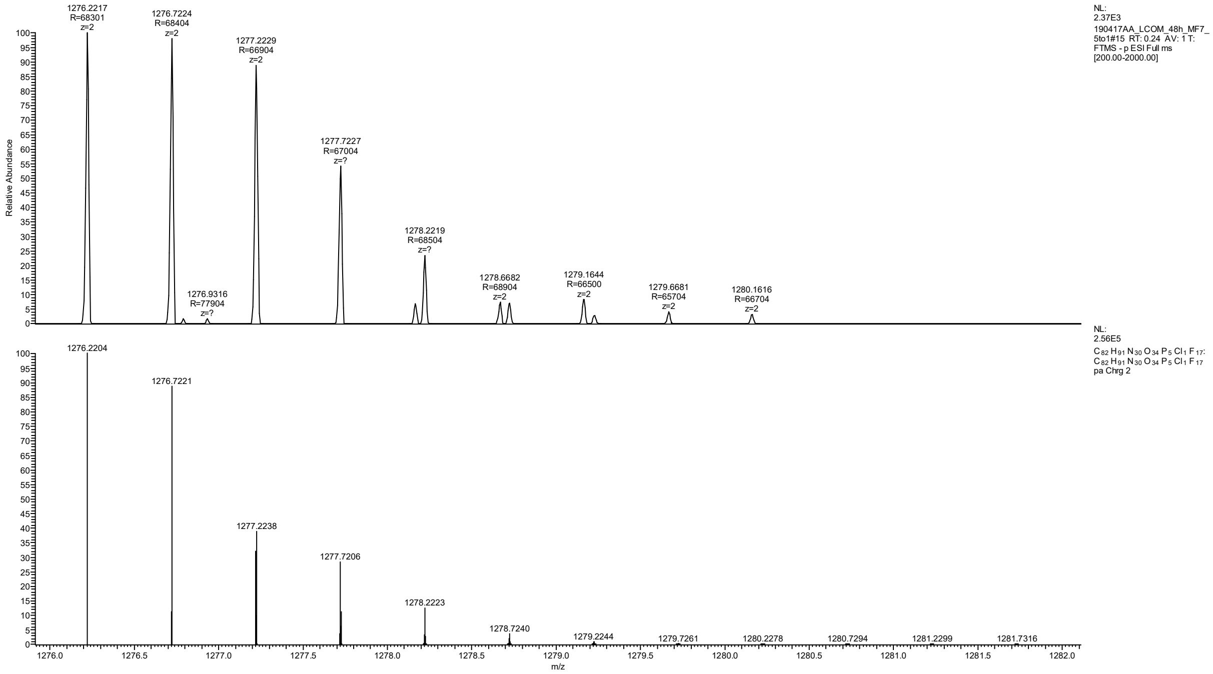


**Figure S24.** Isotopic profile of [*B1* + **3e** − Cl]$^{2-}$

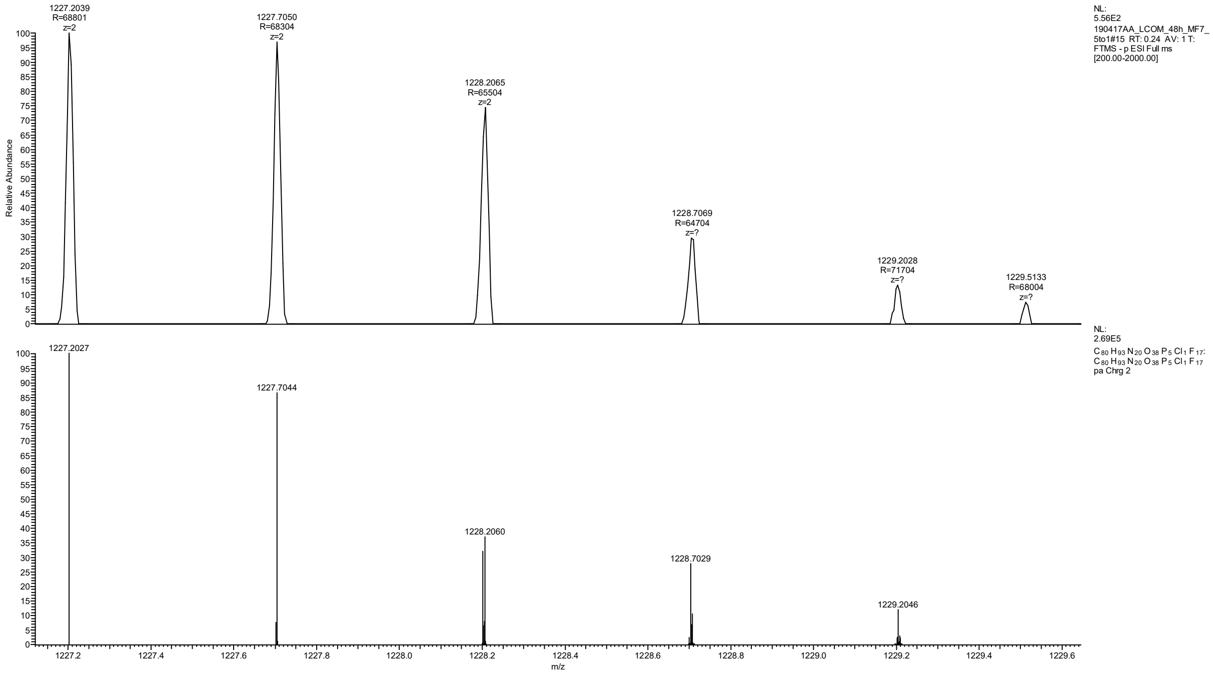


**Figure S25.** Isotopic profile of [*B2* + **3e** − Cl]$^{2-}$.

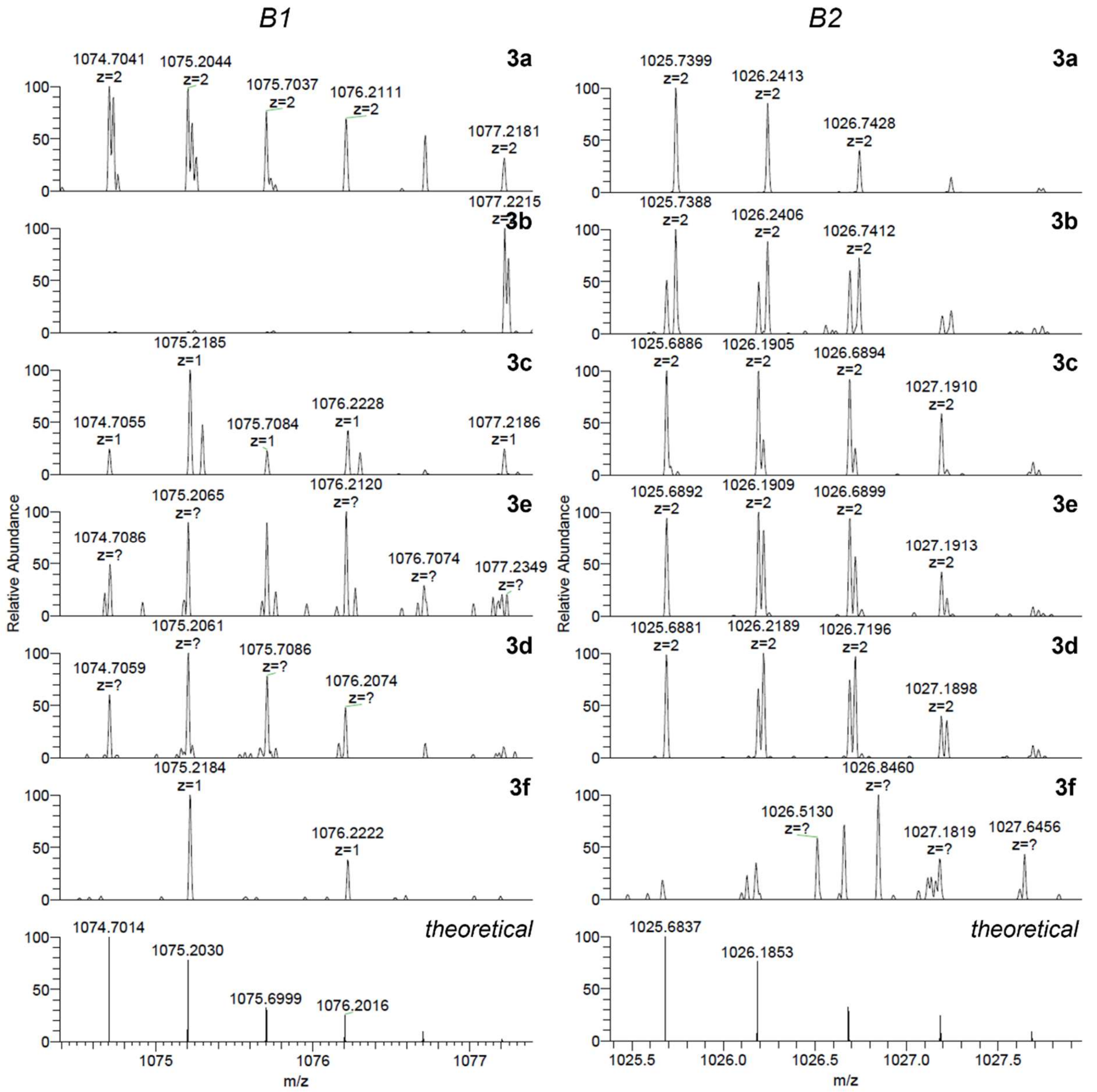


**Figure S26.** Comparison of the Isotopic profiles of the [*B1*/*B2* + **3a-3f** − Cl – $CH_2CH_2(CF_2)_nCF_3]^{2-}$ adducts.

# VI. NMR spectra of compounds *1*, *2a-2f*, *3a-3f*

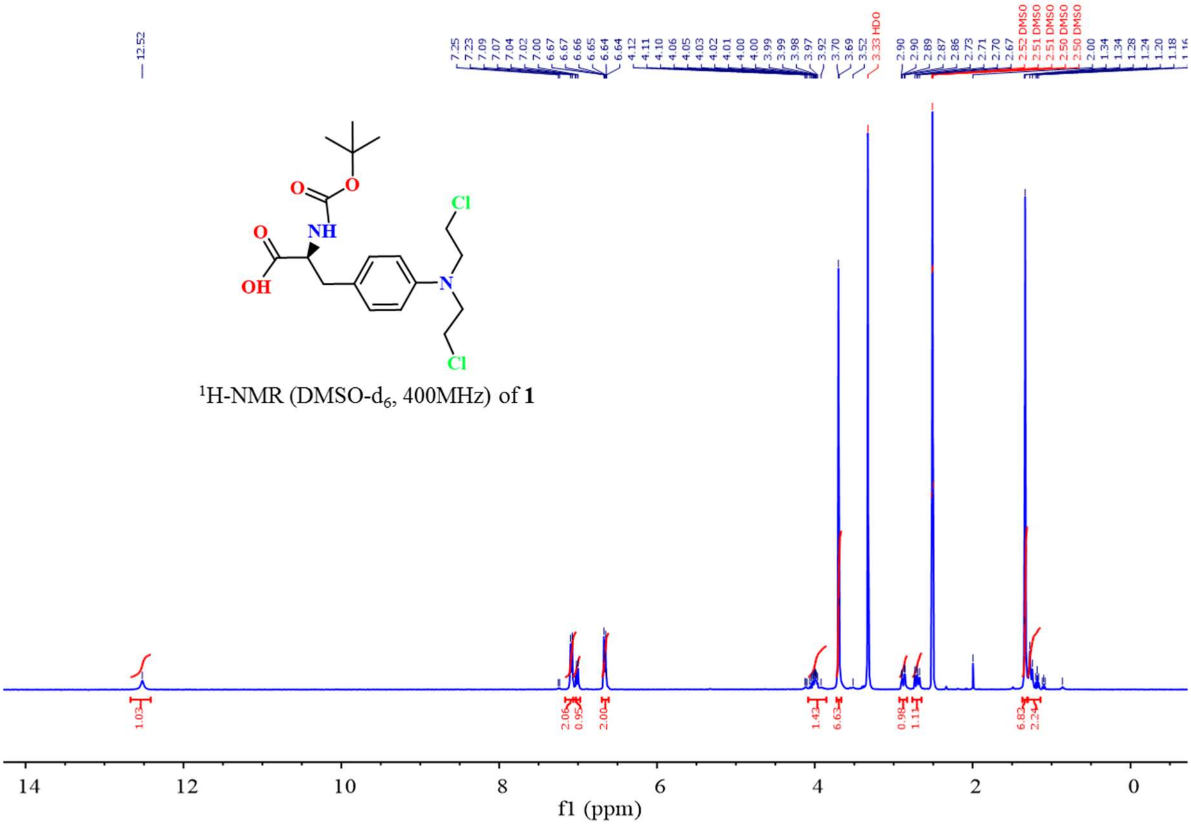


$^{1}$H-NMR (DMSO-$d_6$, 400MHz) of **1**

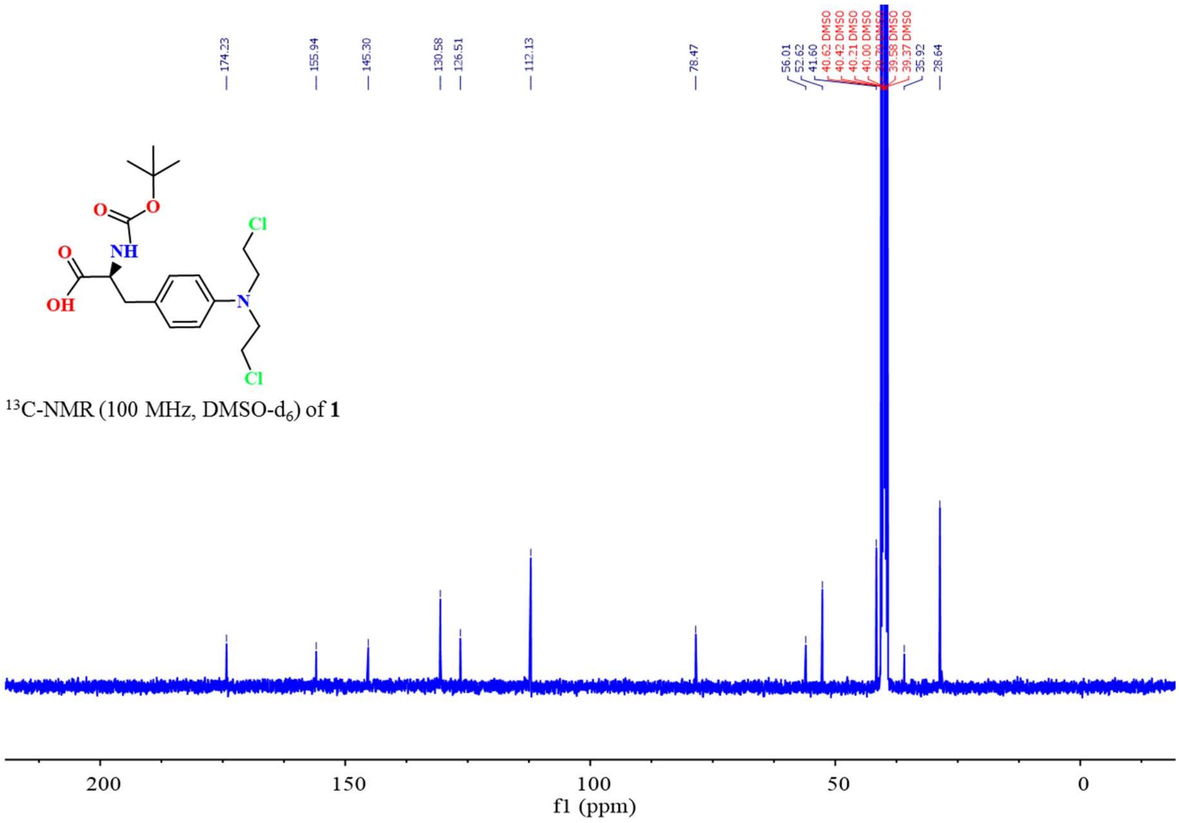


$^{13}$C-NMR (100 MHz, DMSO-$d_6$) of **1**

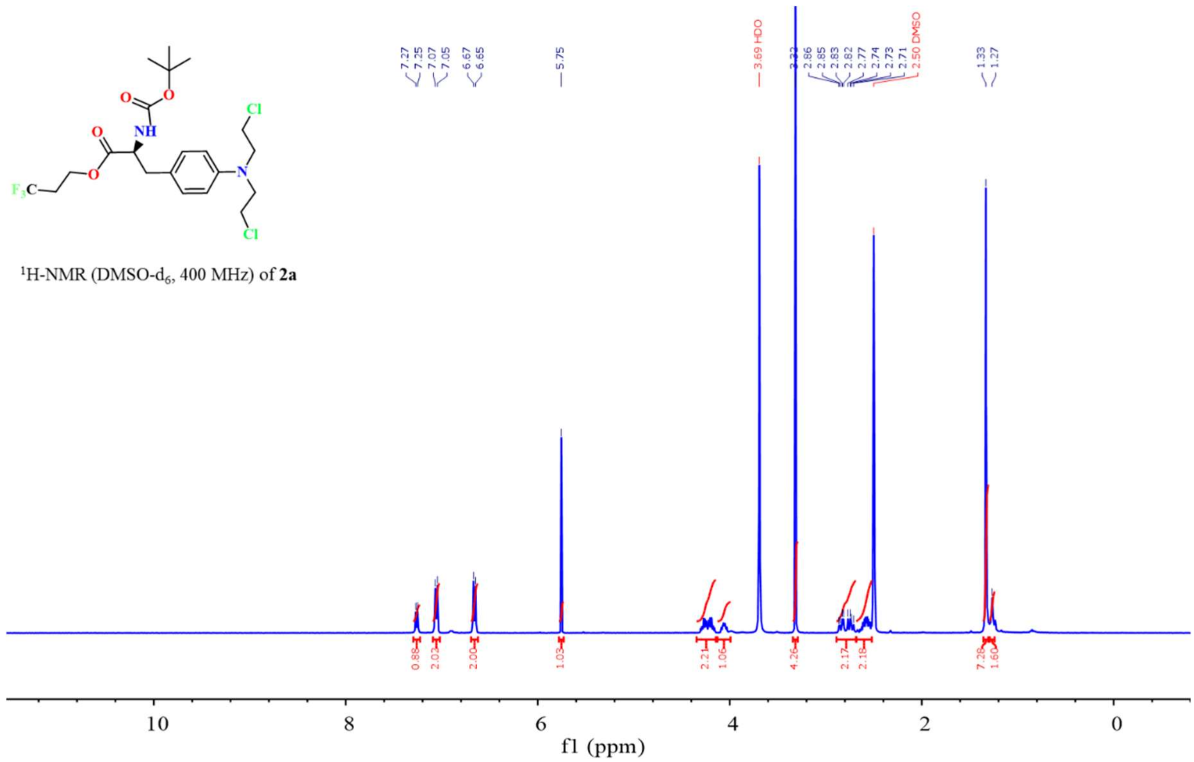


$^{1}$H-NMR (DMSO-$d_6$, 400 MHz) of **2a**

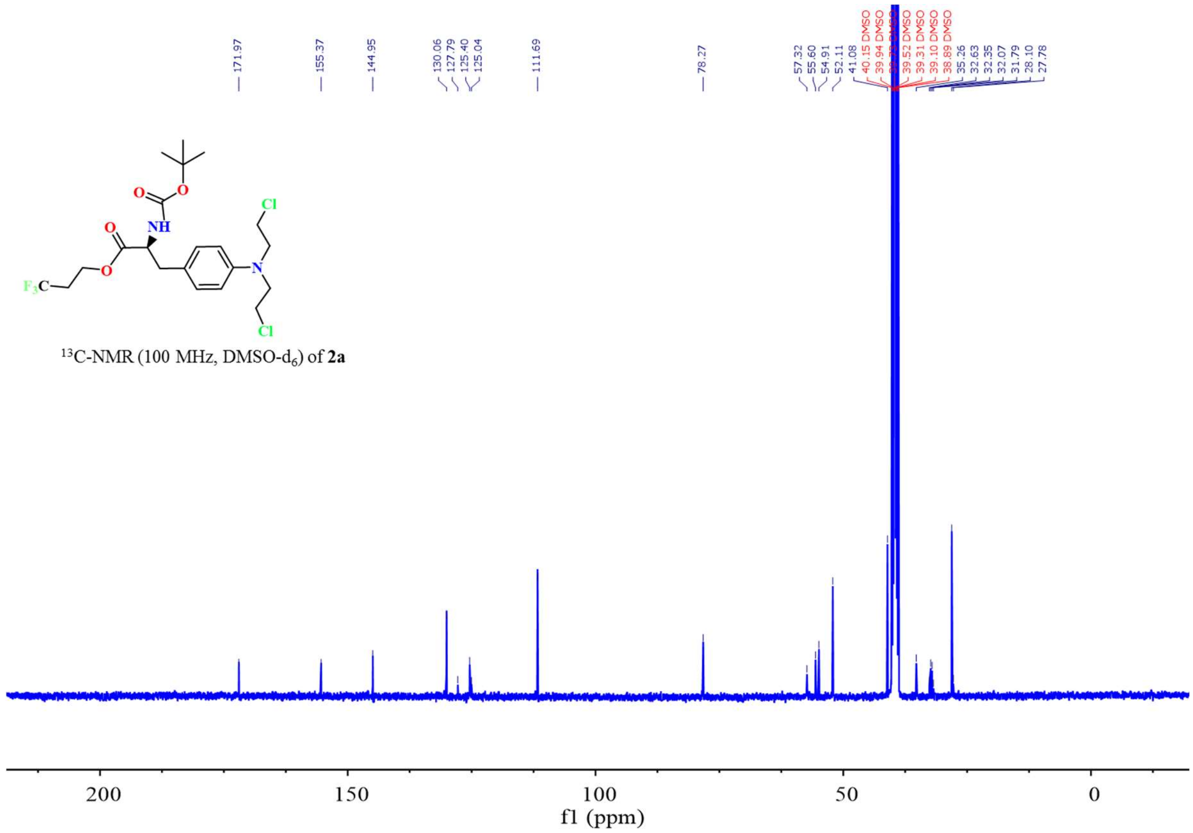


$^{13}$C-NMR (100 MHz, DMSO-$d_6$) of **2a**

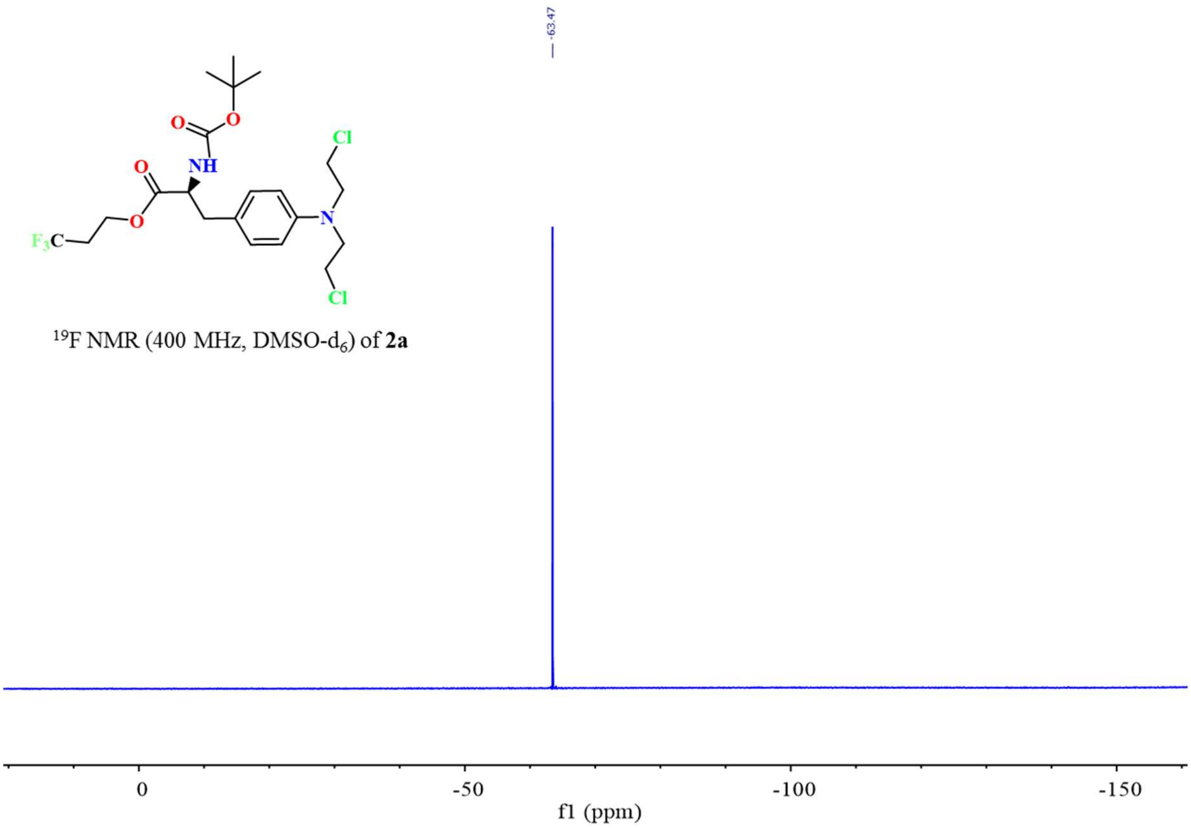

$^{19}$F NMR (400 MHz, DMSO-d$_6$) of **2a**

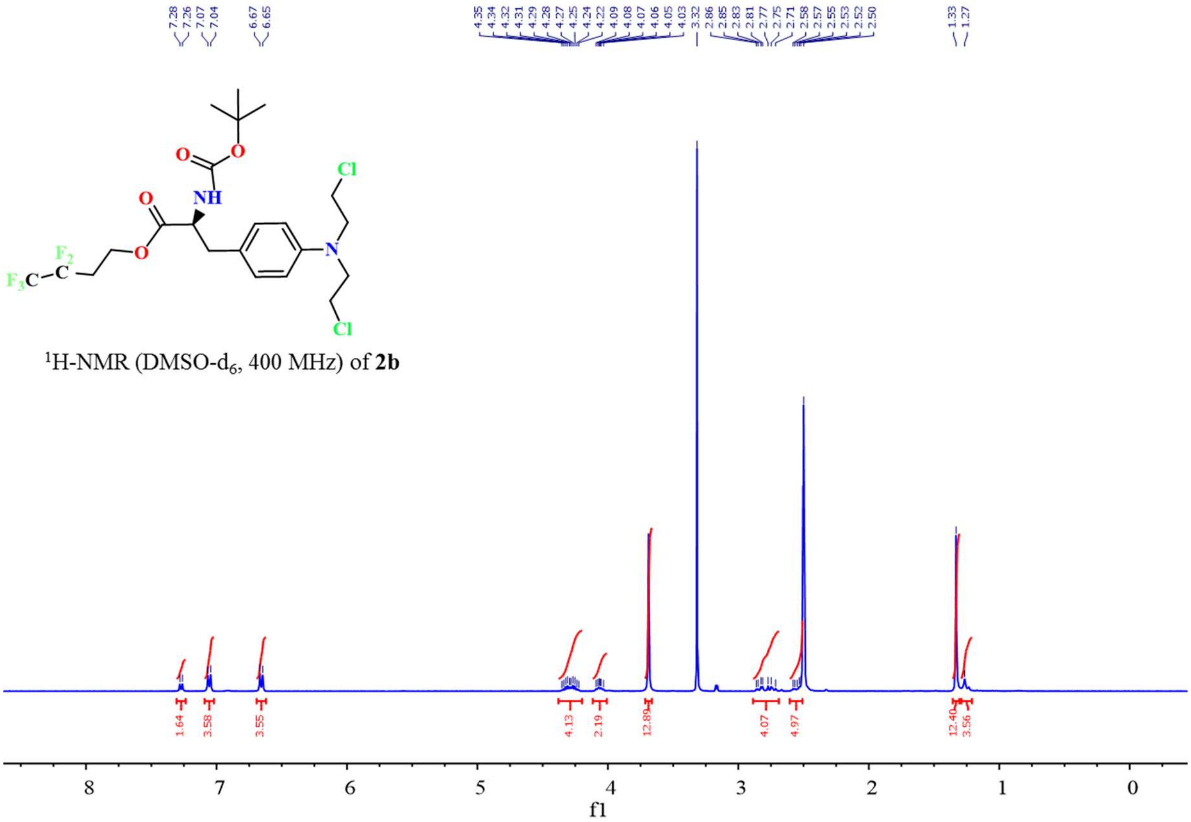

$^{1}$H-NMR (DMSO-d$_6$, 400 MHz) of **2b**

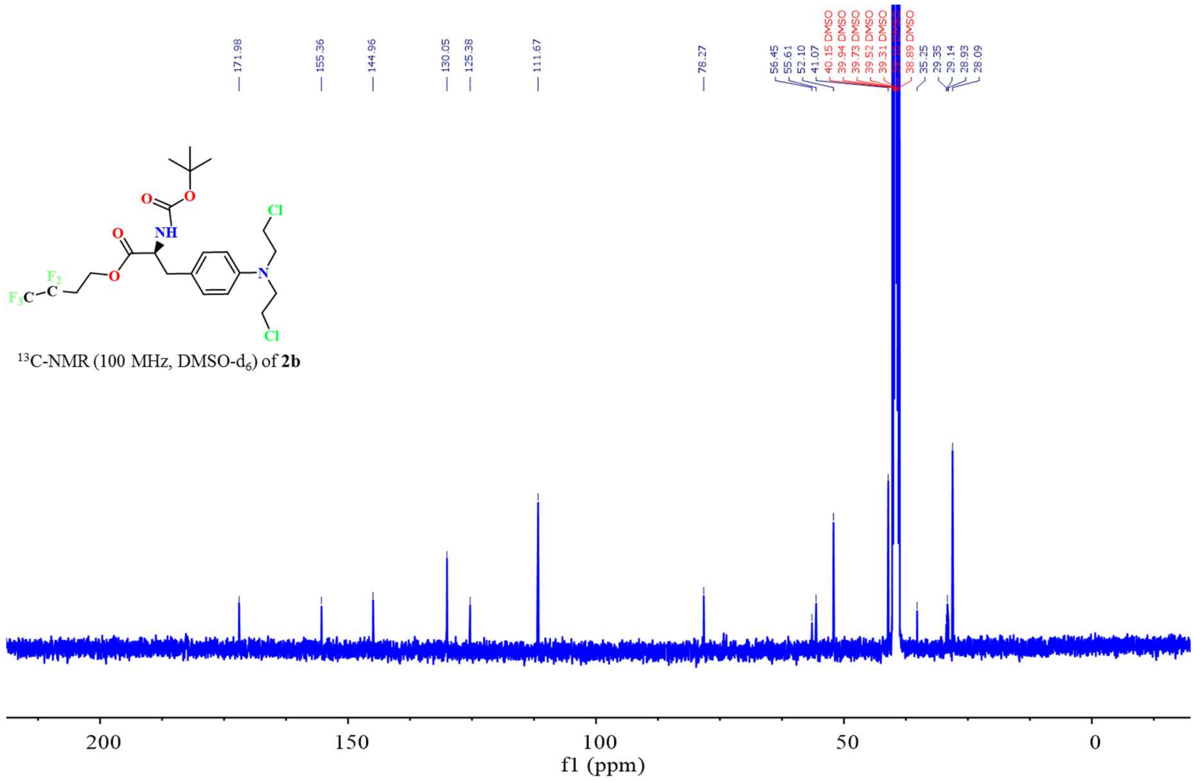

$^{13}C$-NMR (100 MHz, DMSO-$d_6$) of **2b**

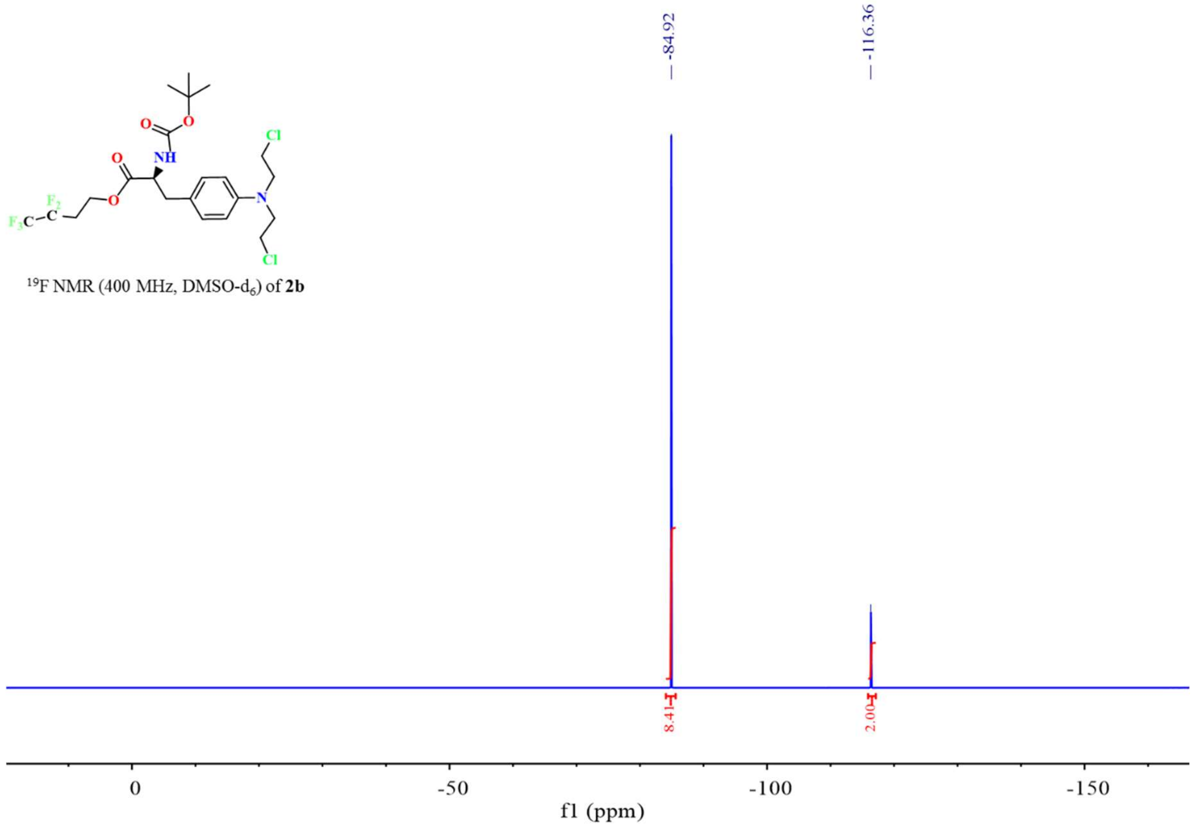

$^{19}F$ NMR (400 MHz, DMSO-$d_6$) of **2b**

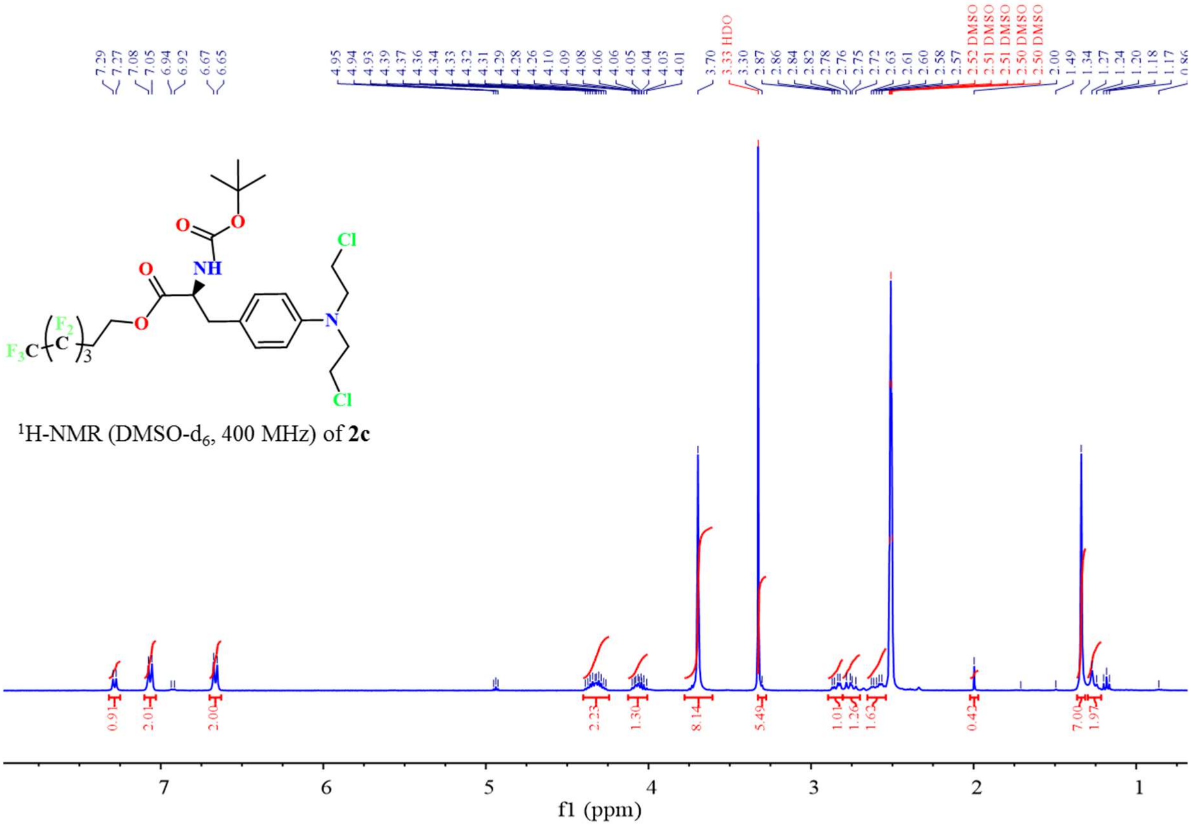


$^{1}$H-NMR (DMSO-$d_6$, 400 MHz) of **2c**

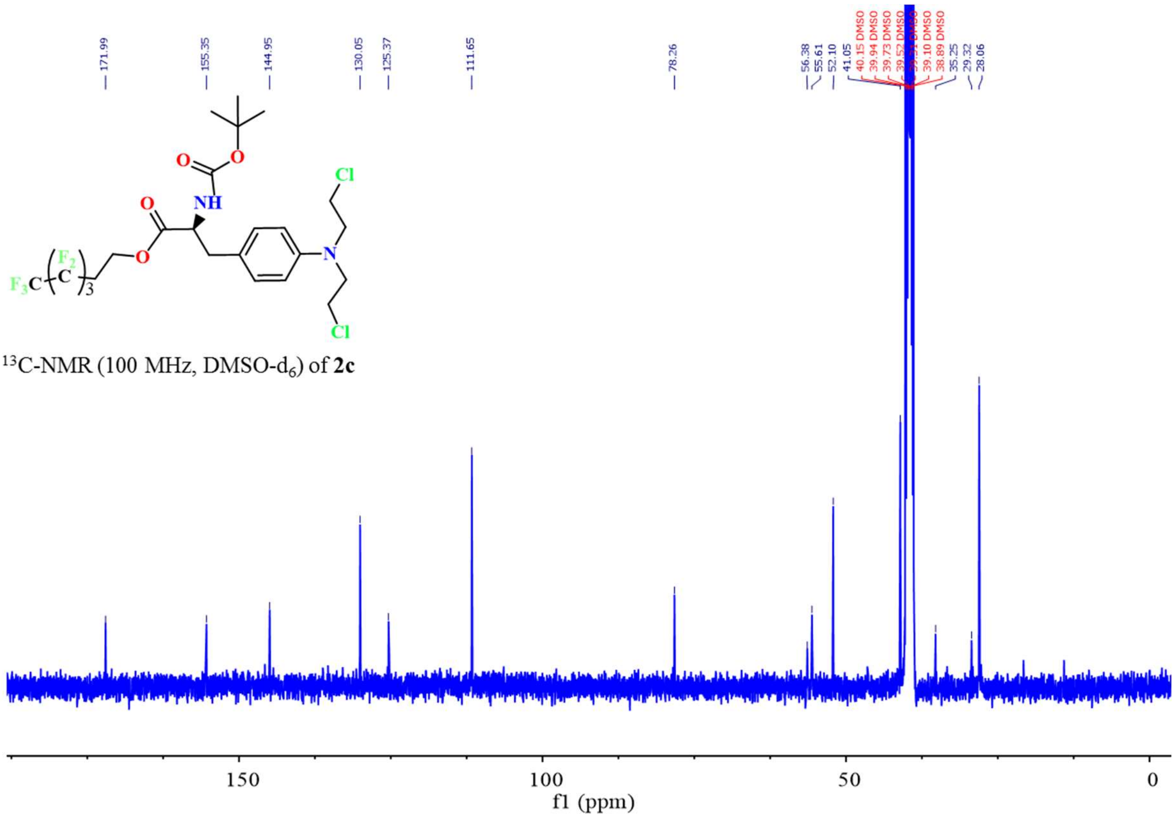


$^{13}$C-NMR (100 MHz, DMSO-$d_6$) of **2c**

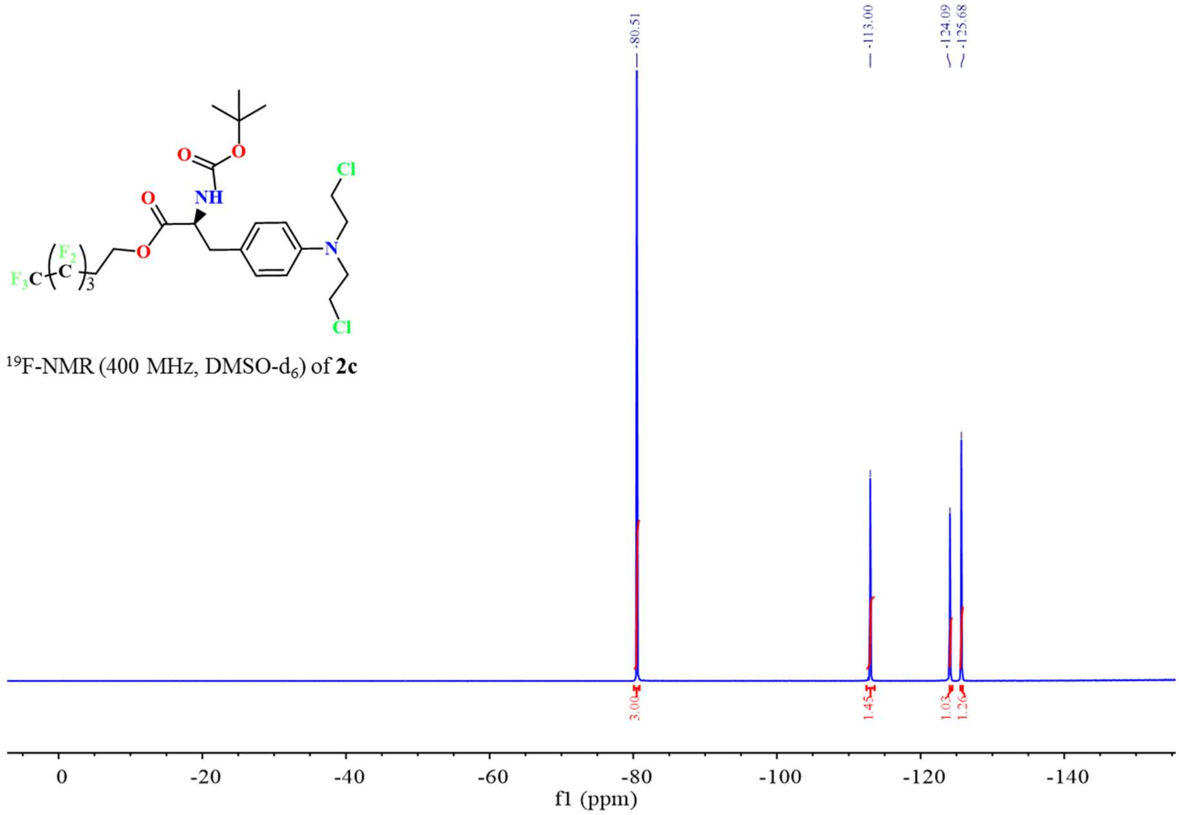


$^{19}$F-NMR (400 MHz, DMSO-d$_6$) of **2c**

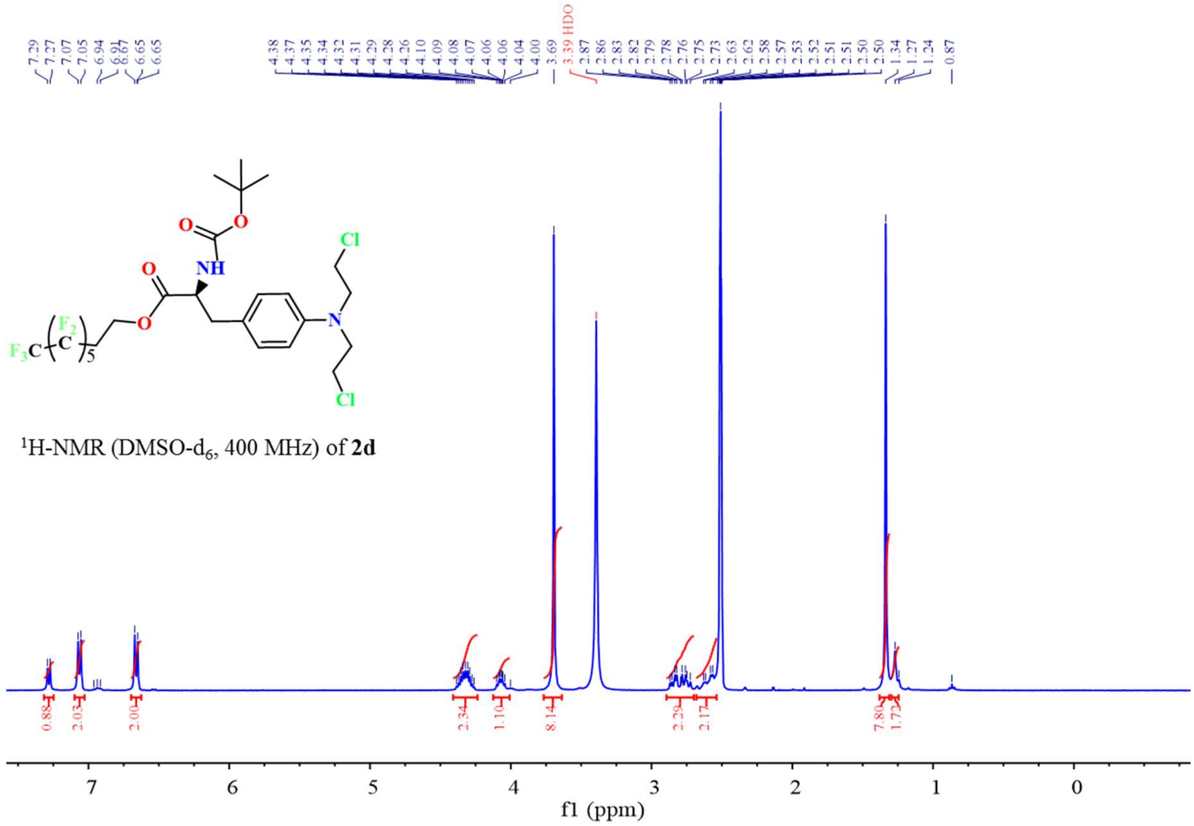


$^{1}$H-NMR (DMSO-d$_6$, 400 MHz) of **2d**

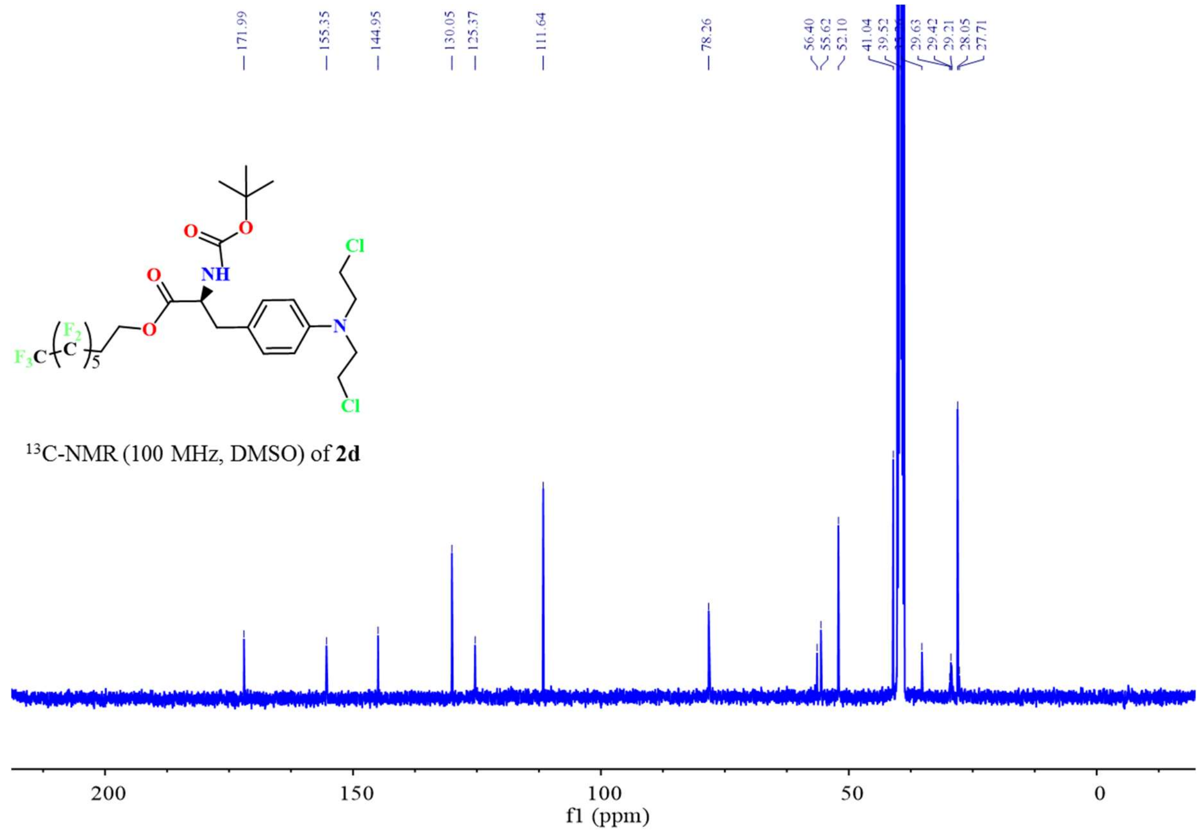


$^{13}$C-NMR (100 MHz, DMSO) of **2d**

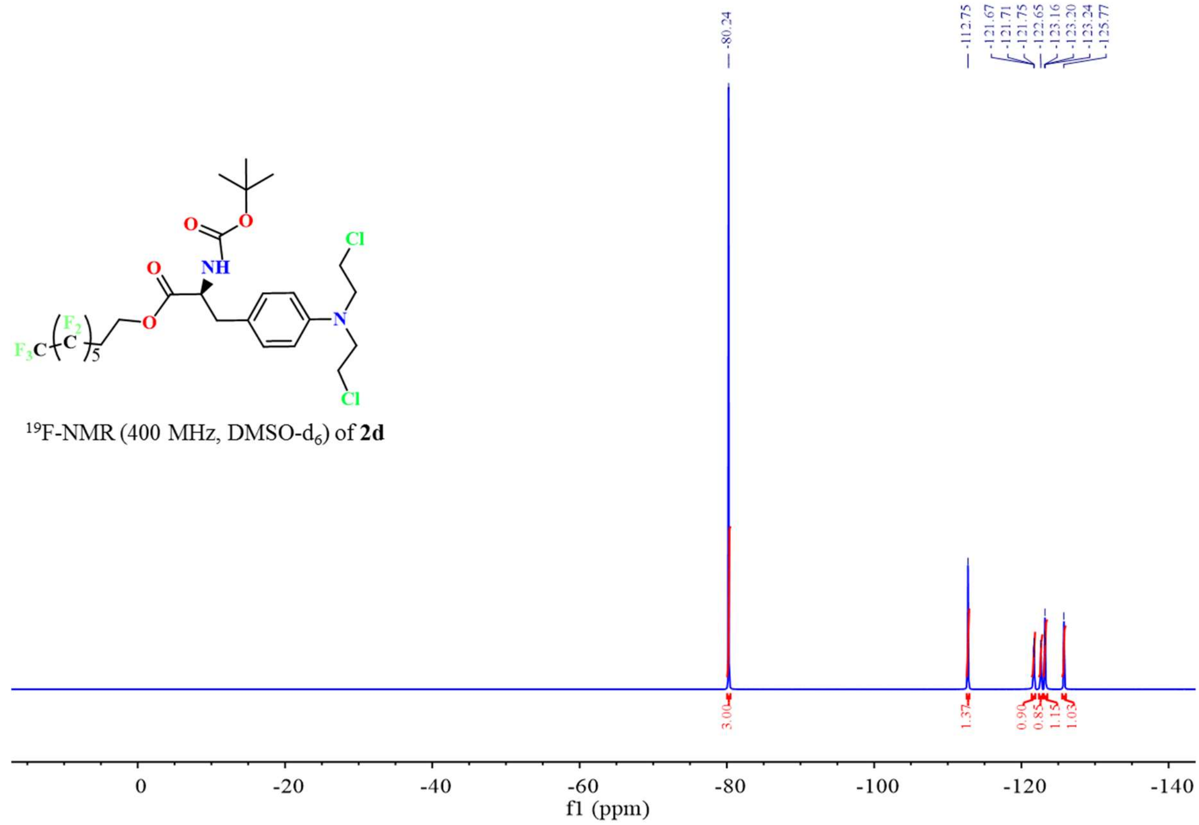


$^{19}$F-NMR (400 MHz, DMSO-$d_6$) of **2d**

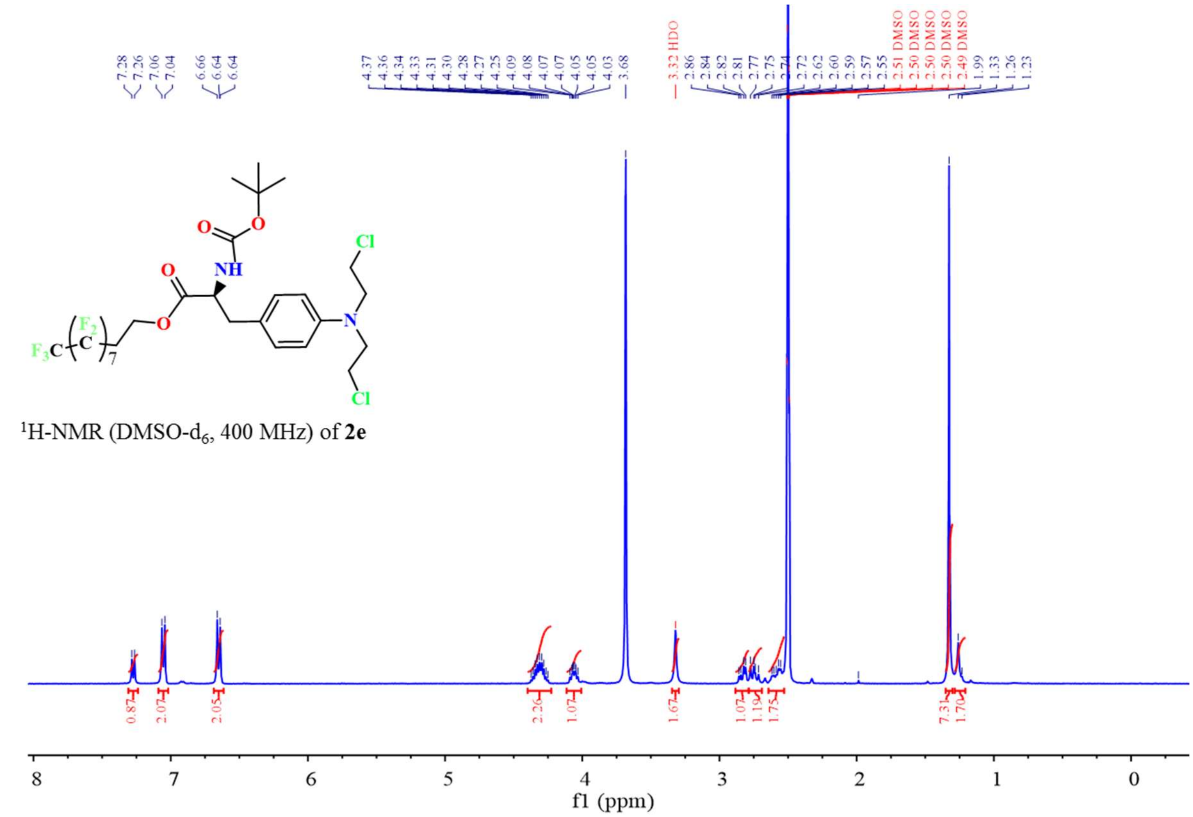


$^{1}$H-NMR (DMSO-$d_6$, 400 MHz) of **2e**

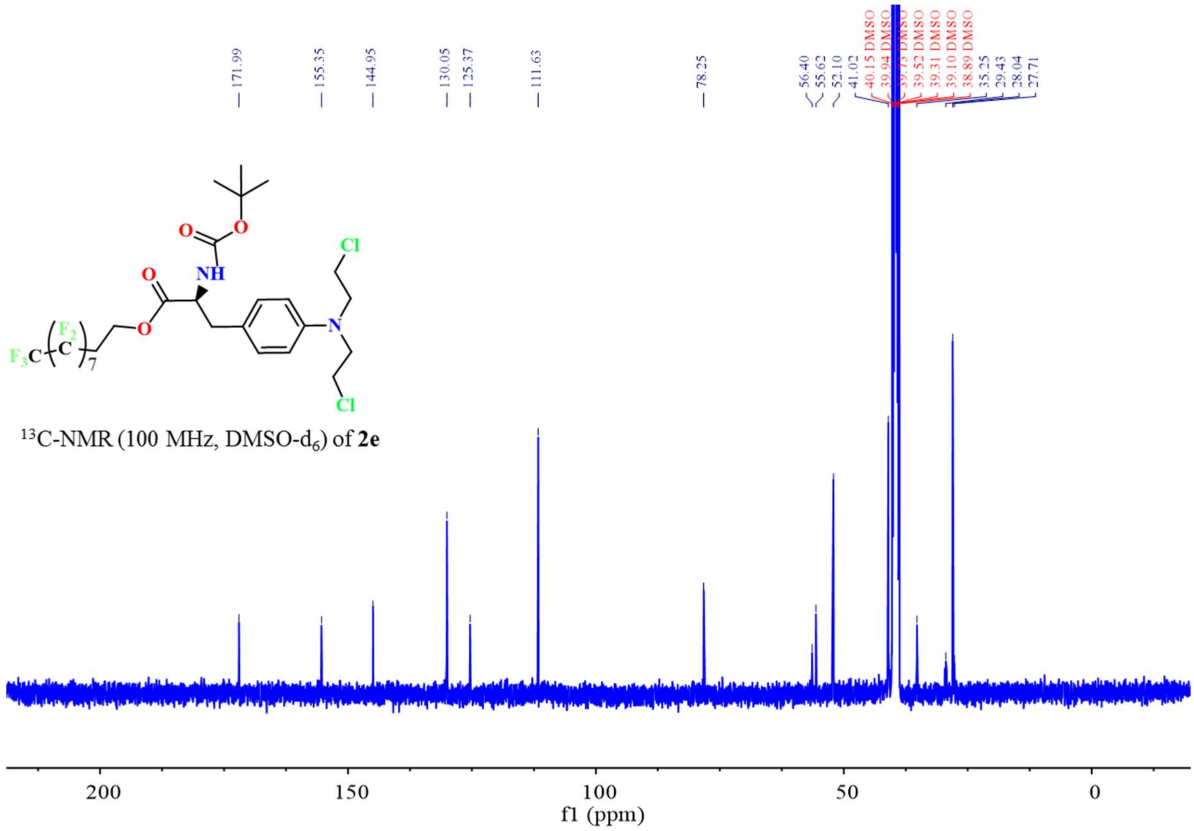


$^{13}$C-NMR (100 MHz, DMSO-$d_6$) of **2e**

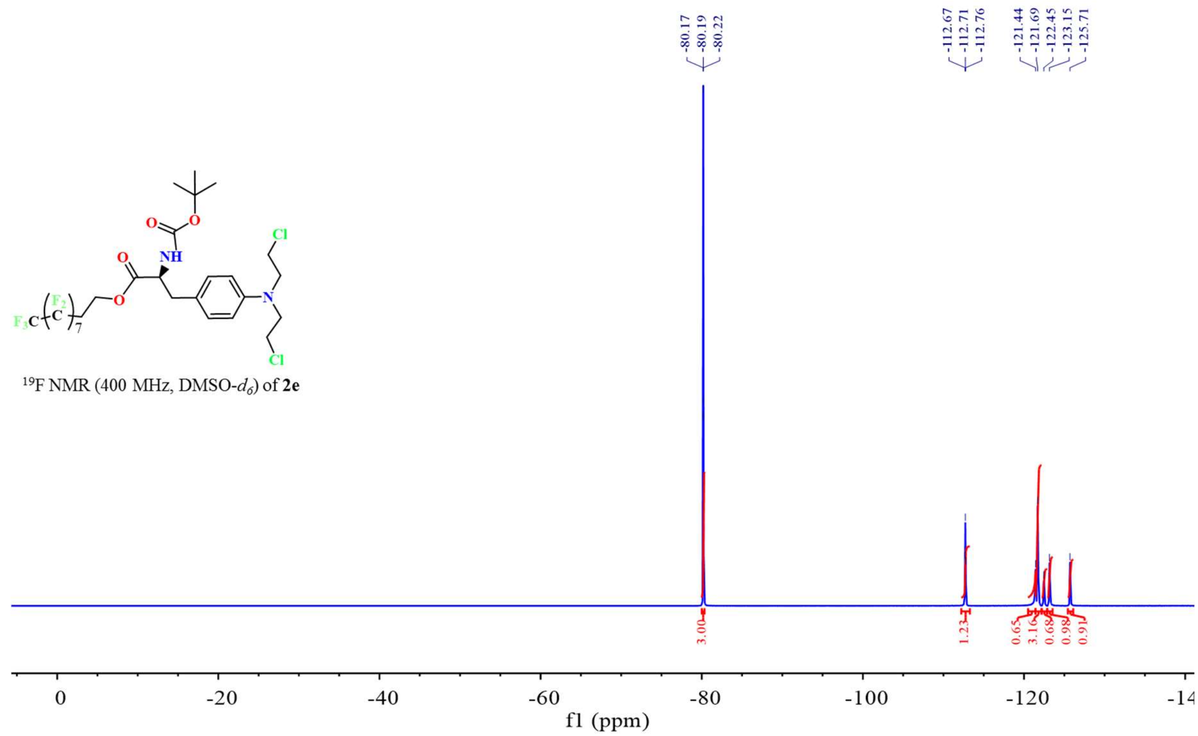


$^{19}$F NMR (400 MHz, DMSO-$d_6$) of **2e**

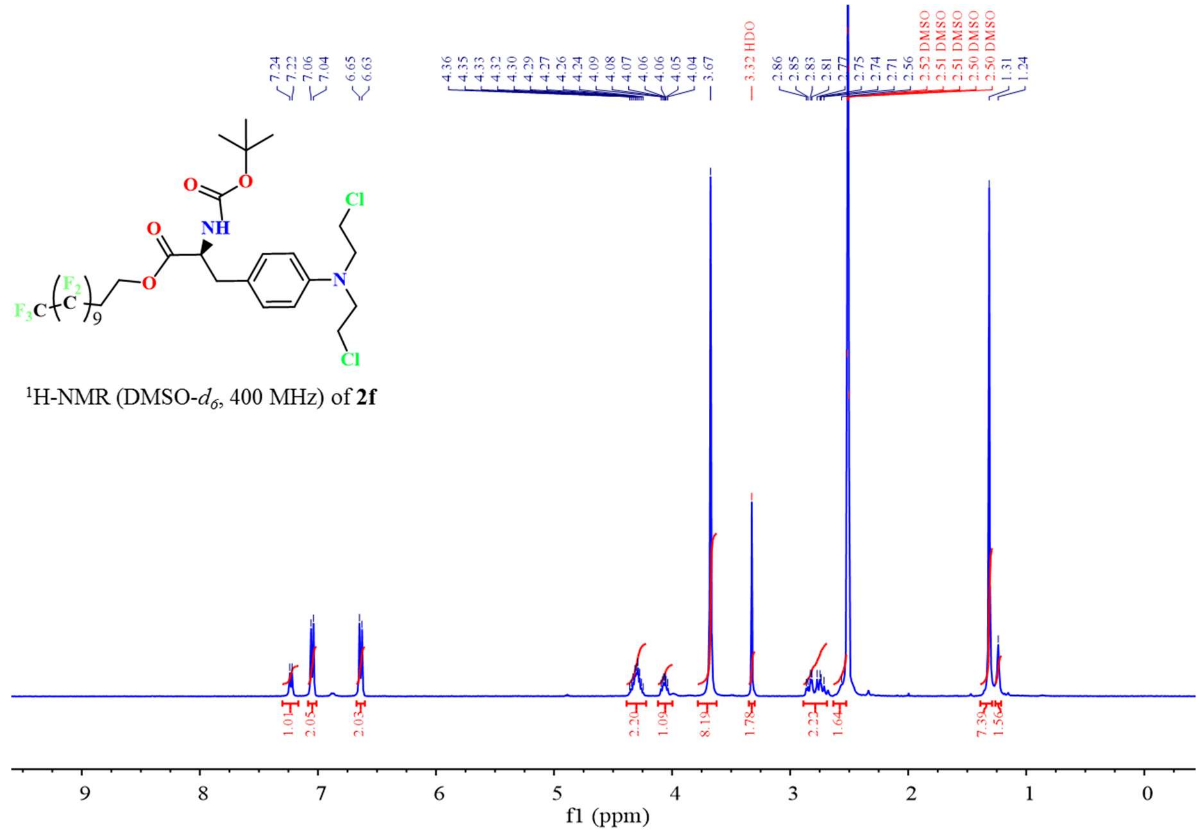


$^{1}$H-NMR (DMSO-$d_6$, 400 MHz) of **2f**

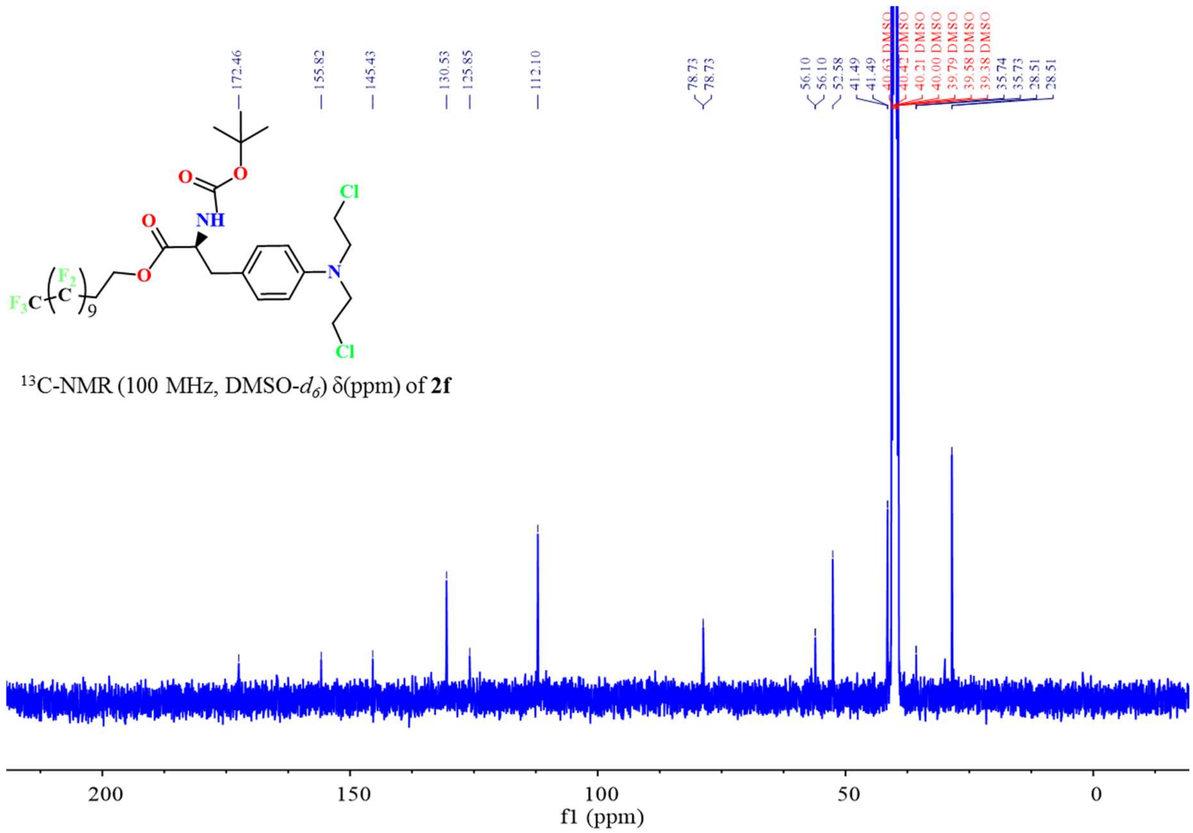


$^{13}$C-NMR (100 MHz, DMSO-$d_6$) δ(ppm) of **2f**

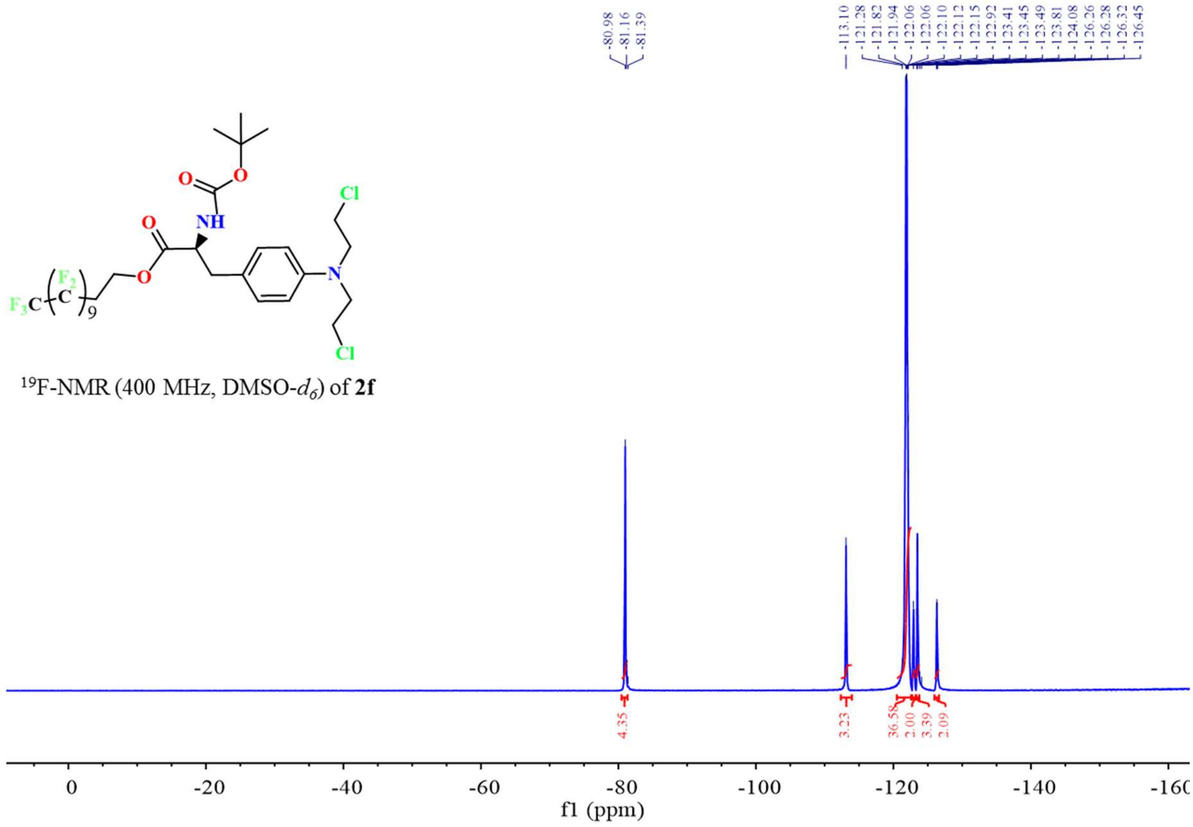


$^{19}$F-NMR (400 MHz, DMSO-$d_6$) of **2f**

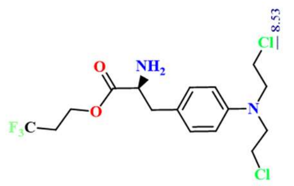

$^{1}$H-NMR (DMSO-$d_6$, 400 MHz) of **3a**

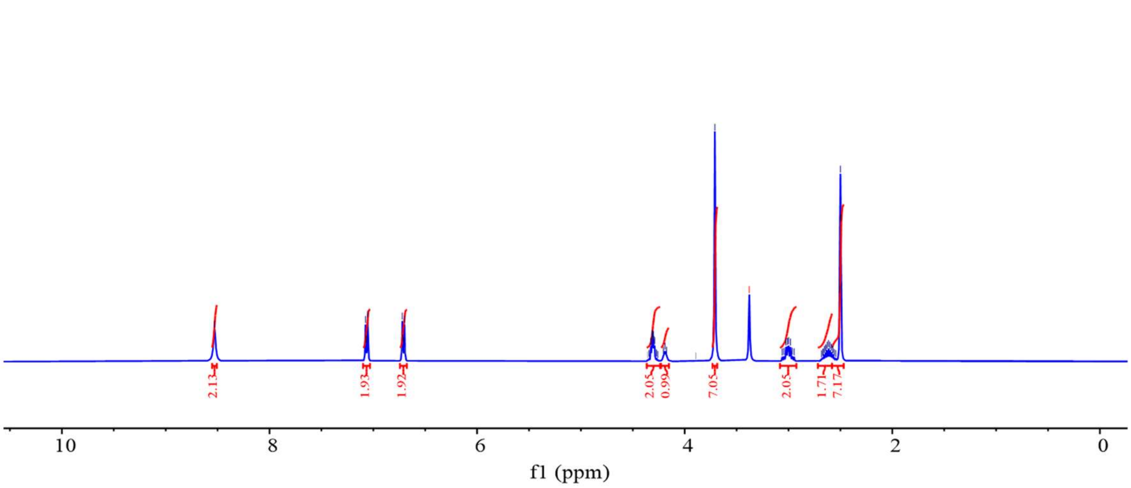

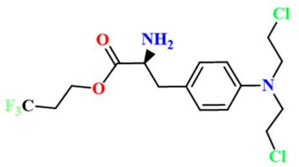

$^{13}$C-NMR (100 MHz, DMSO-$d_6$) of **3a**

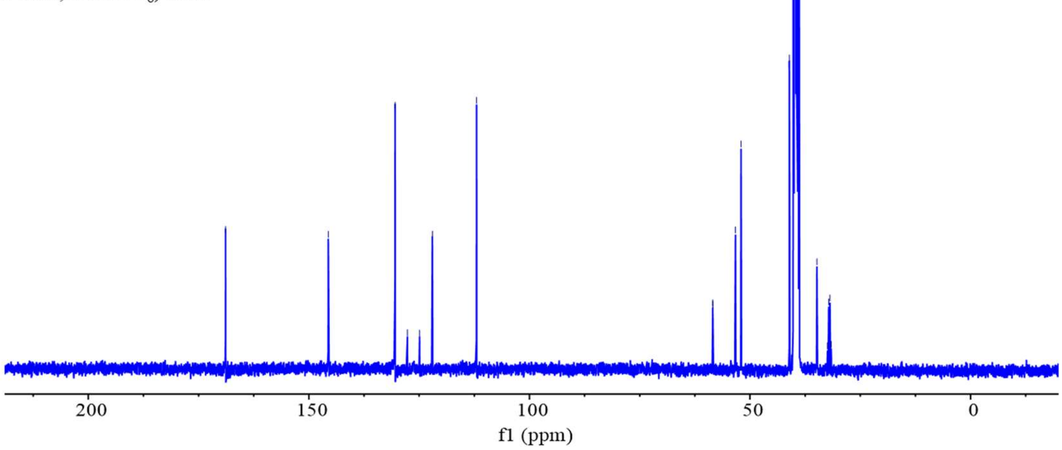

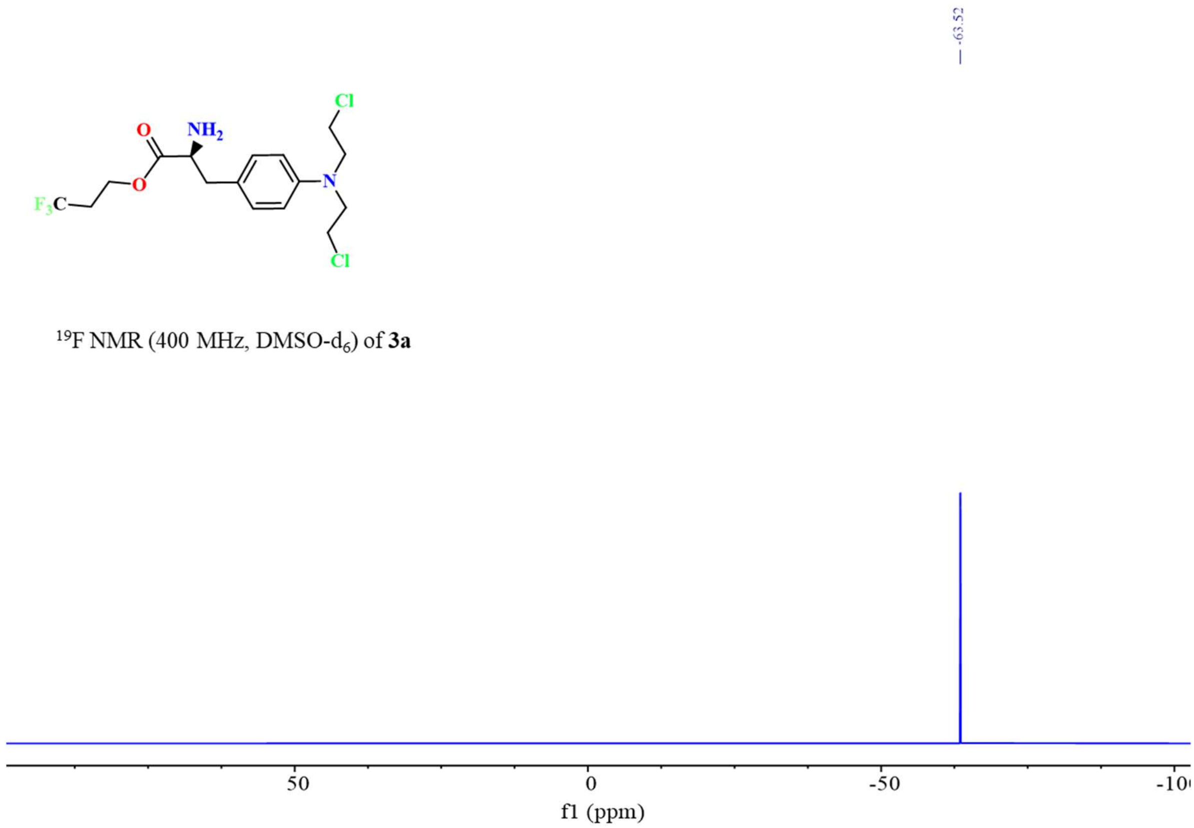

$^{19}$F NMR (400 MHz, DMSO-d$_6$) of **3a**

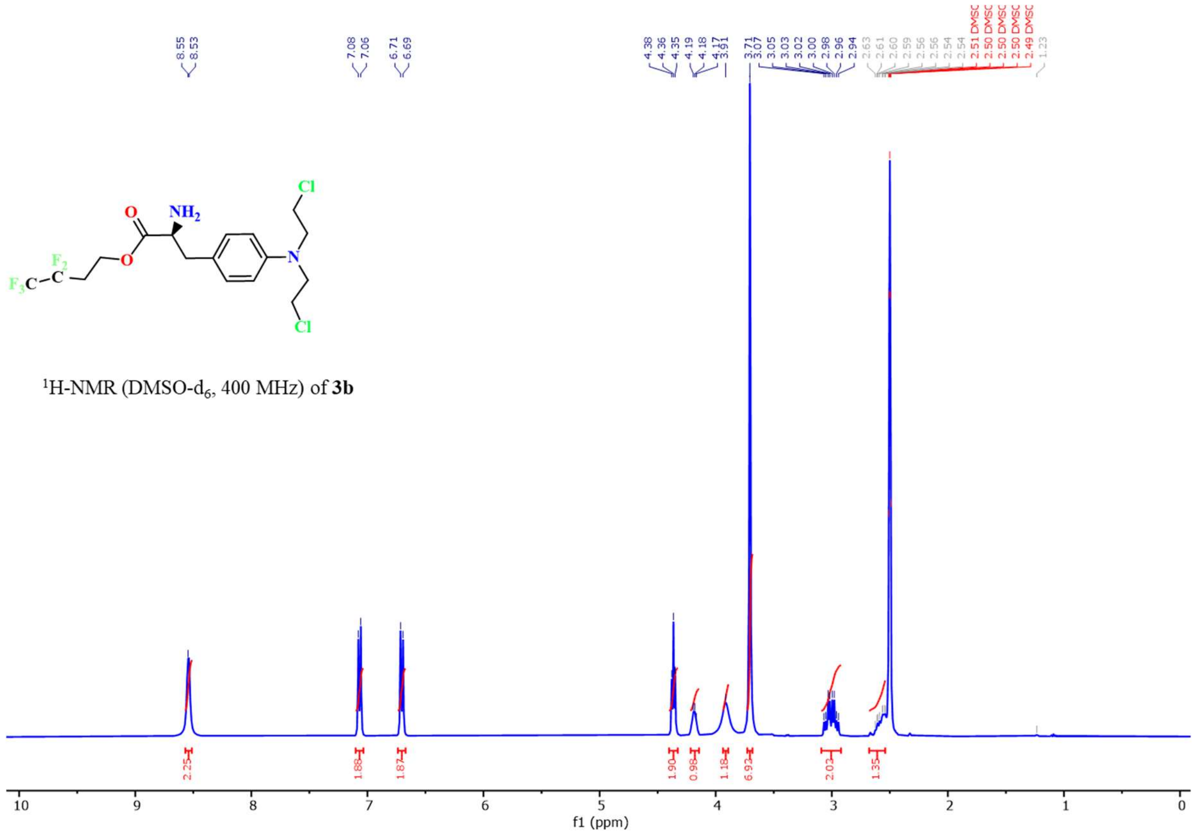

$^{1}$H-NMR (DMSO-d$_6$, 400 MHz) of **3b**

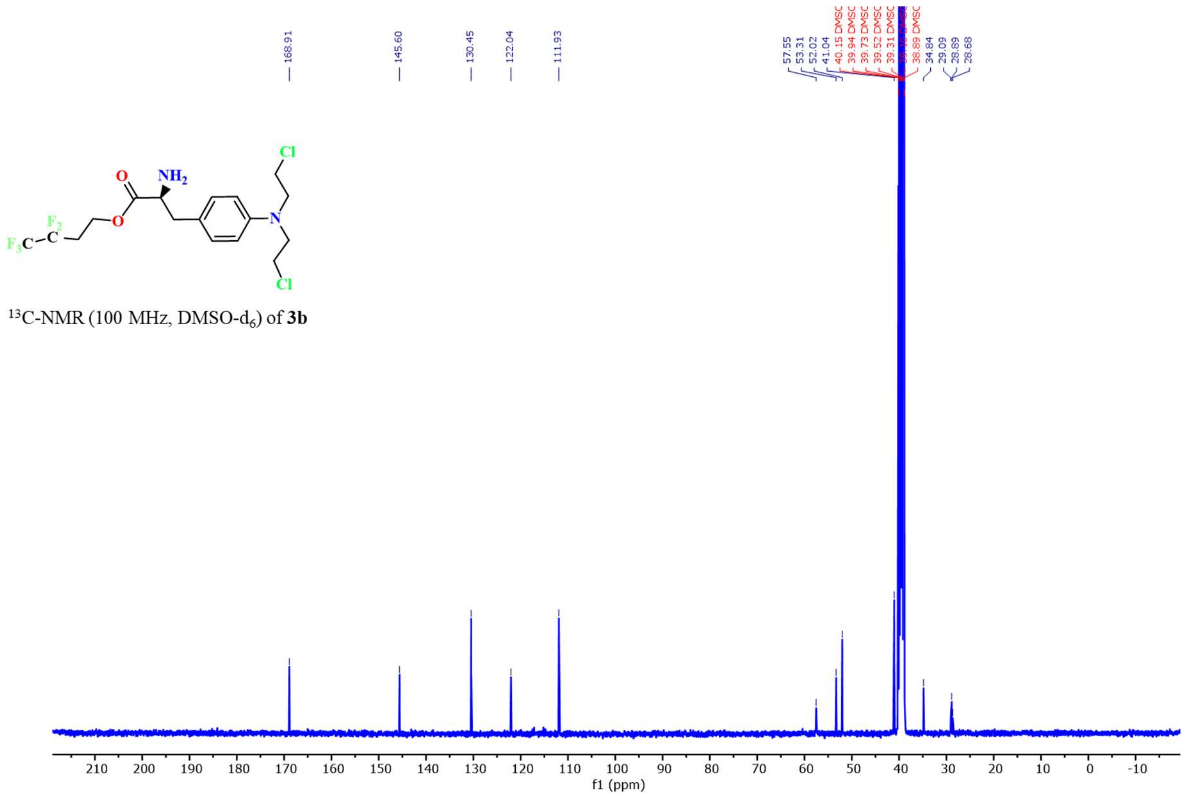


$^{13}$C-NMR (100 MHz, DMSO-d$_6$) of **3b**

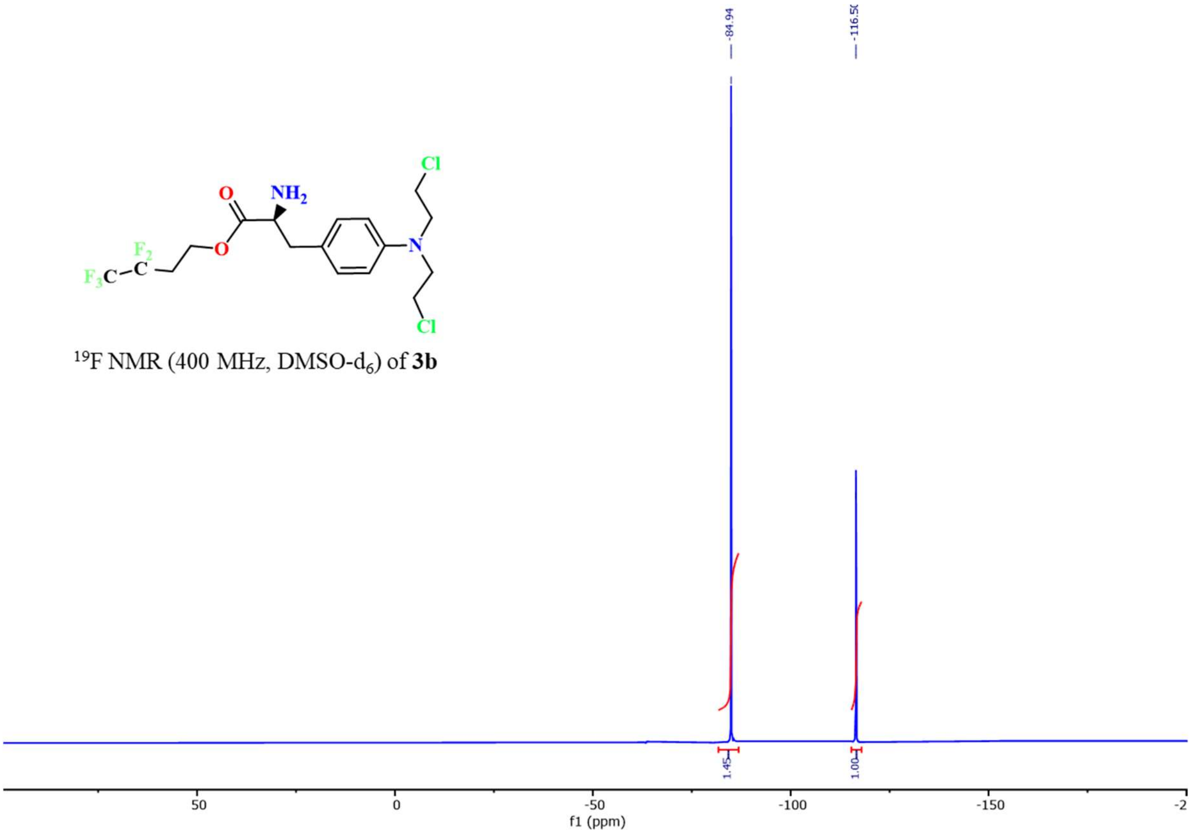


$^{19}$F NMR (400 MHz, DMSO-d$_6$) of **3b**

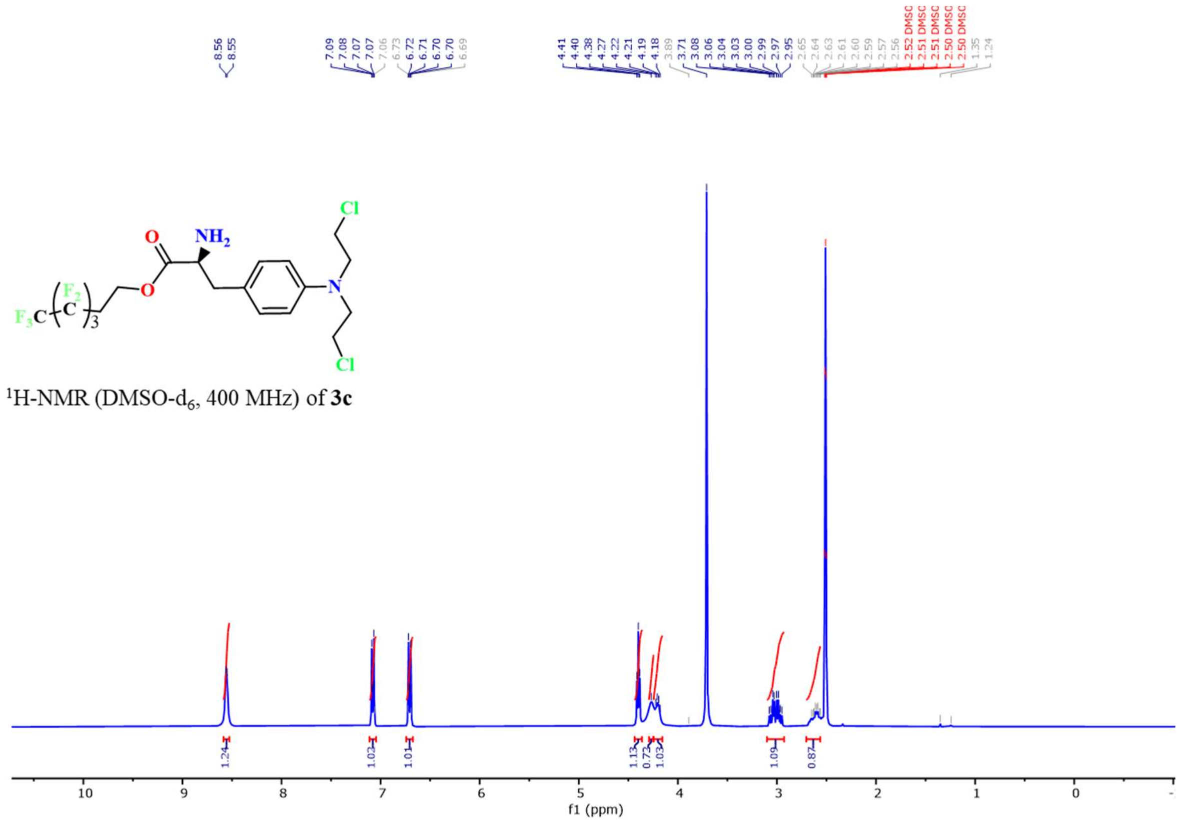


$^{1}$H-NMR (DMSO-$d_6$, 400 MHz) of **3c**

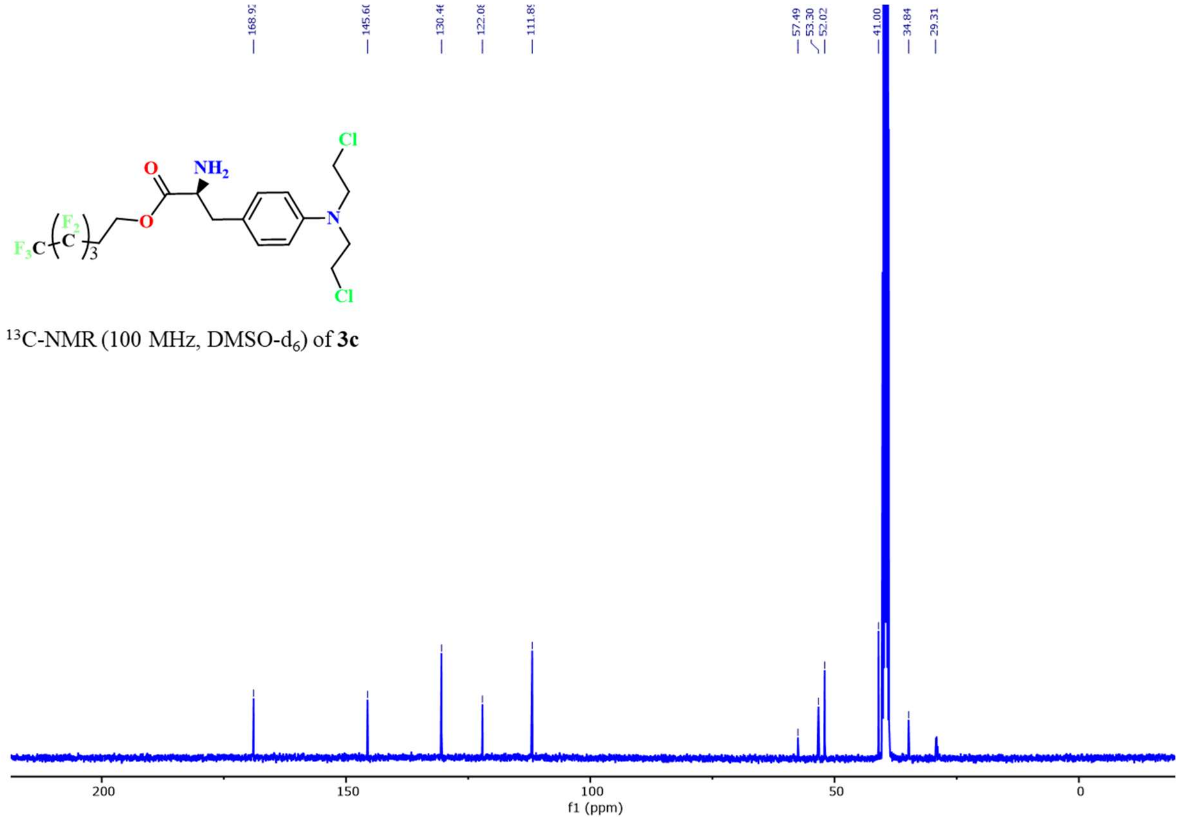


$^{13}$C-NMR (100 MHz, DMSO-$d_6$) of **3c**

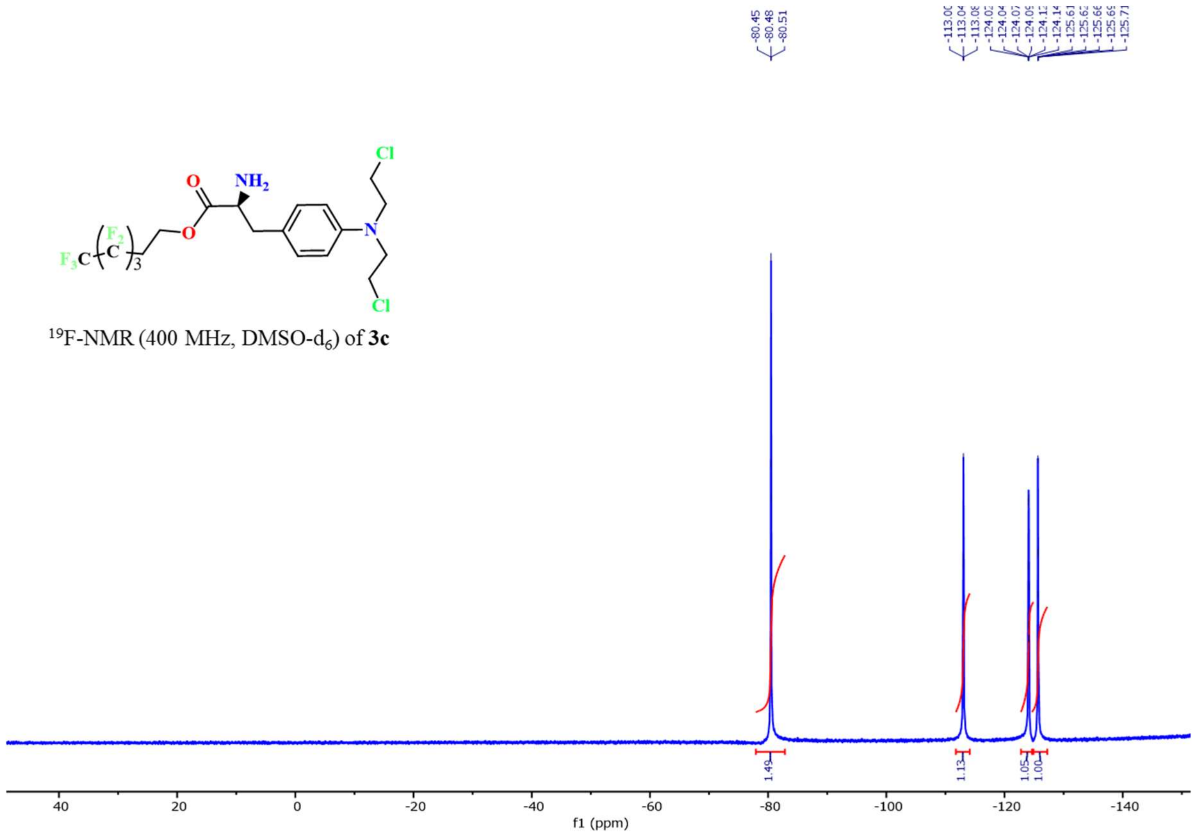


$^{19}$F-NMR (400 MHz, DMSO-d$_6$) of **3c**

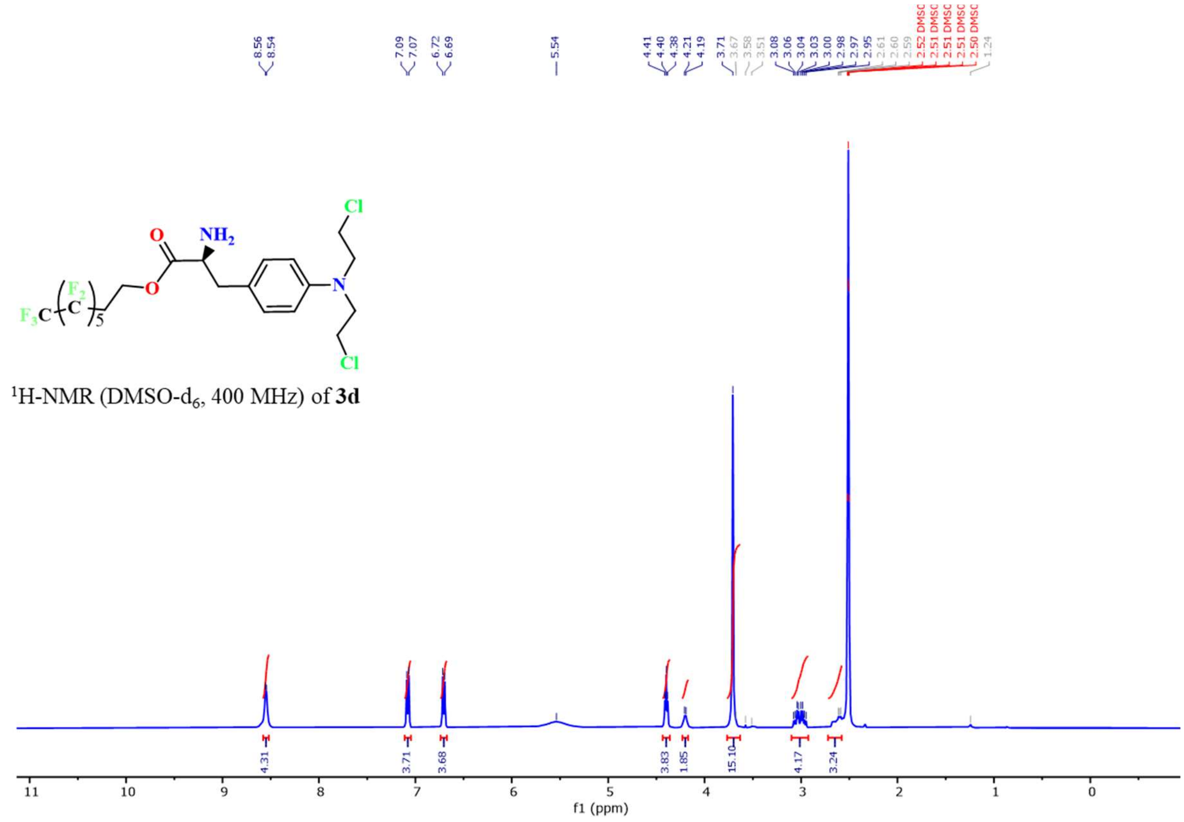


$^{1}$H-NMR (DMSO-d$_6$, 400 MHz) of **3d**

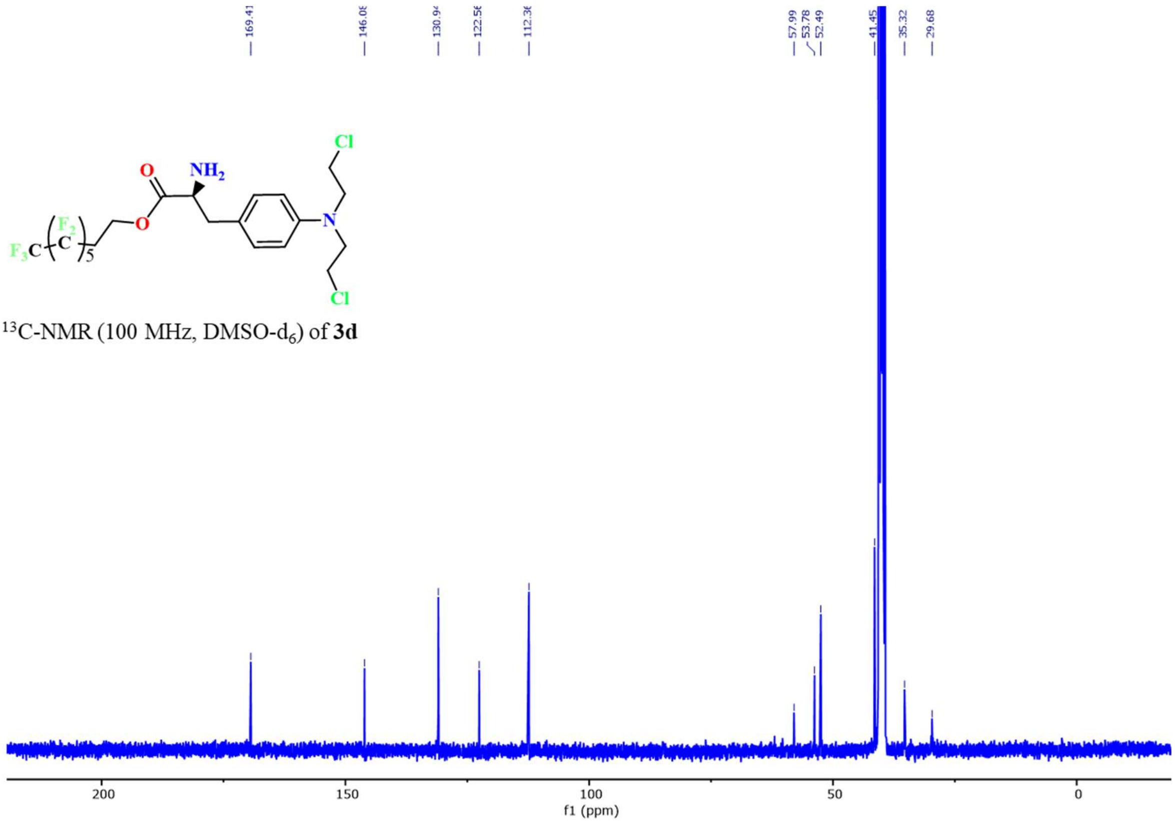


$^{13}$C-NMR (100 MHz, DMSO-d$_6$) of **3d**

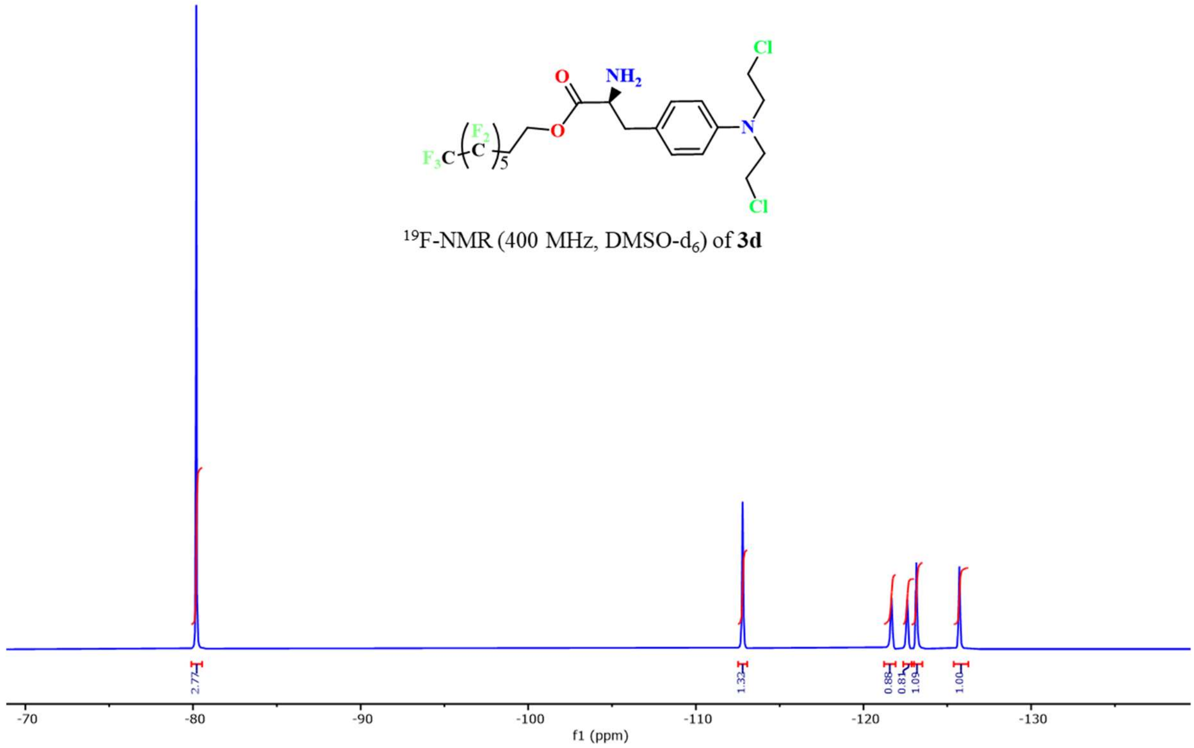


$^{19}$F-NMR (400 MHz, DMSO-d$_6$) of **3d**

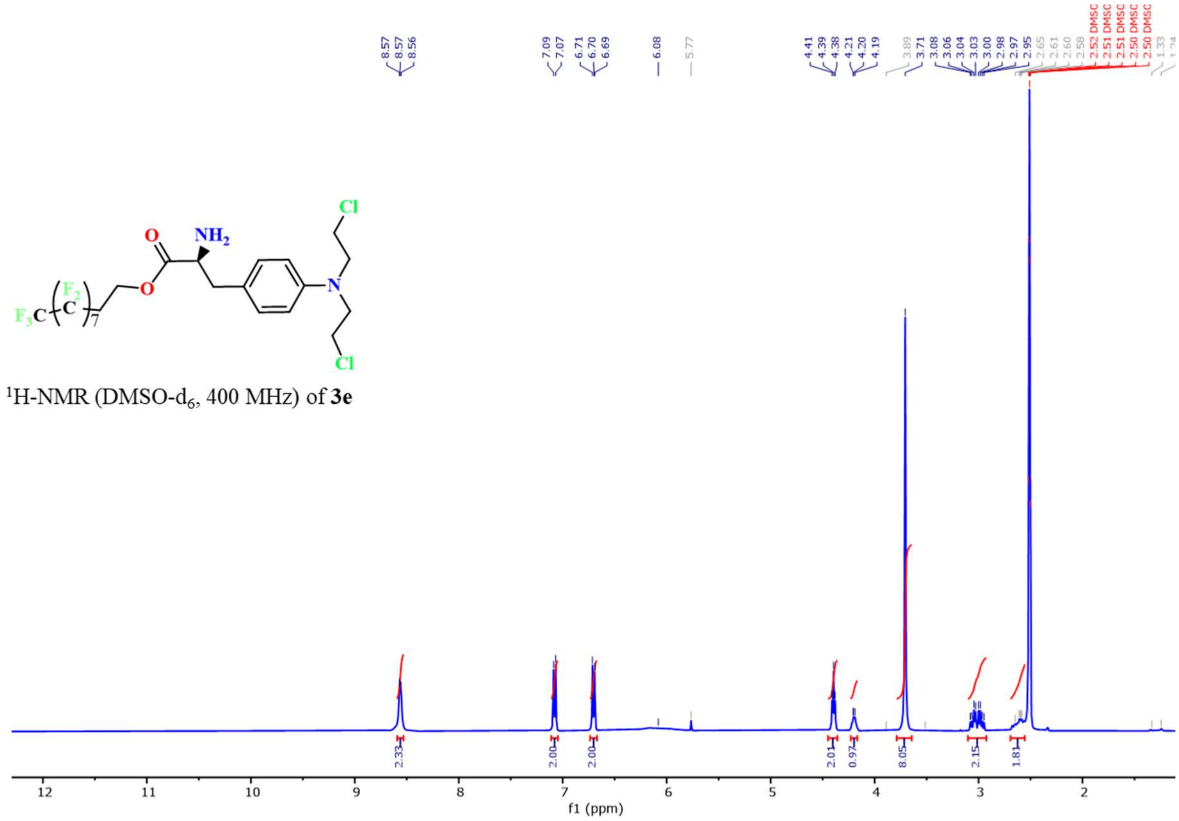


$^{1}$H-NMR (DMSO-d$_6$, 400 MHz) of **3e**

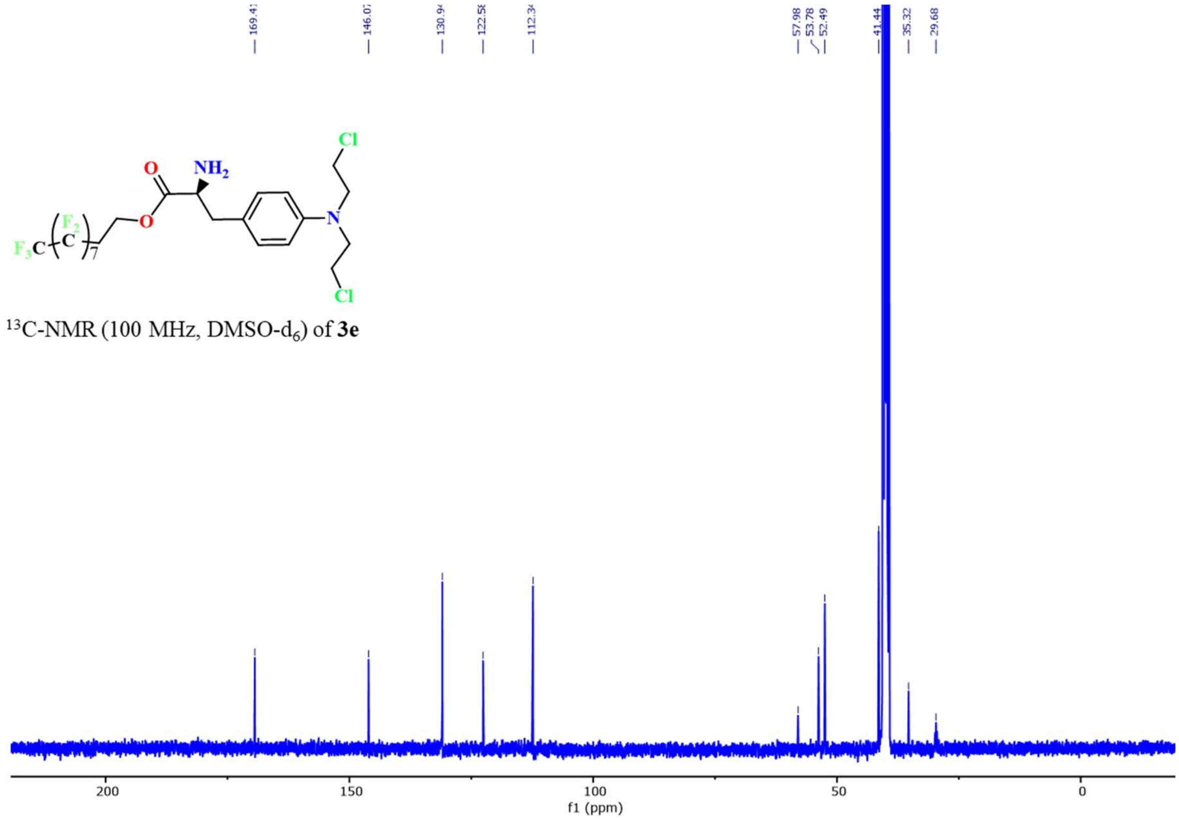


$^{13}$C-NMR (100 MHz, DMSO-d$_6$) of **3e**

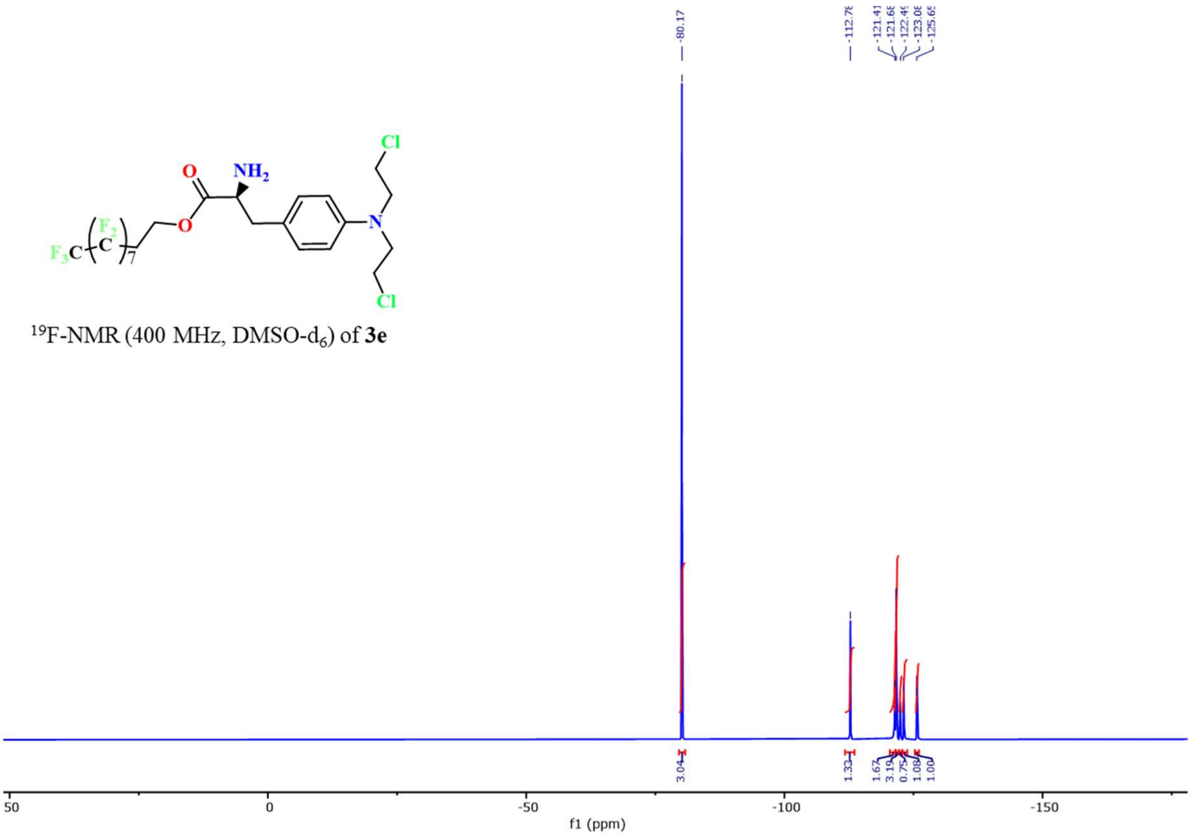


$^{19}$F-NMR (400 MHz, DMSO-d$_6$) of **3e**

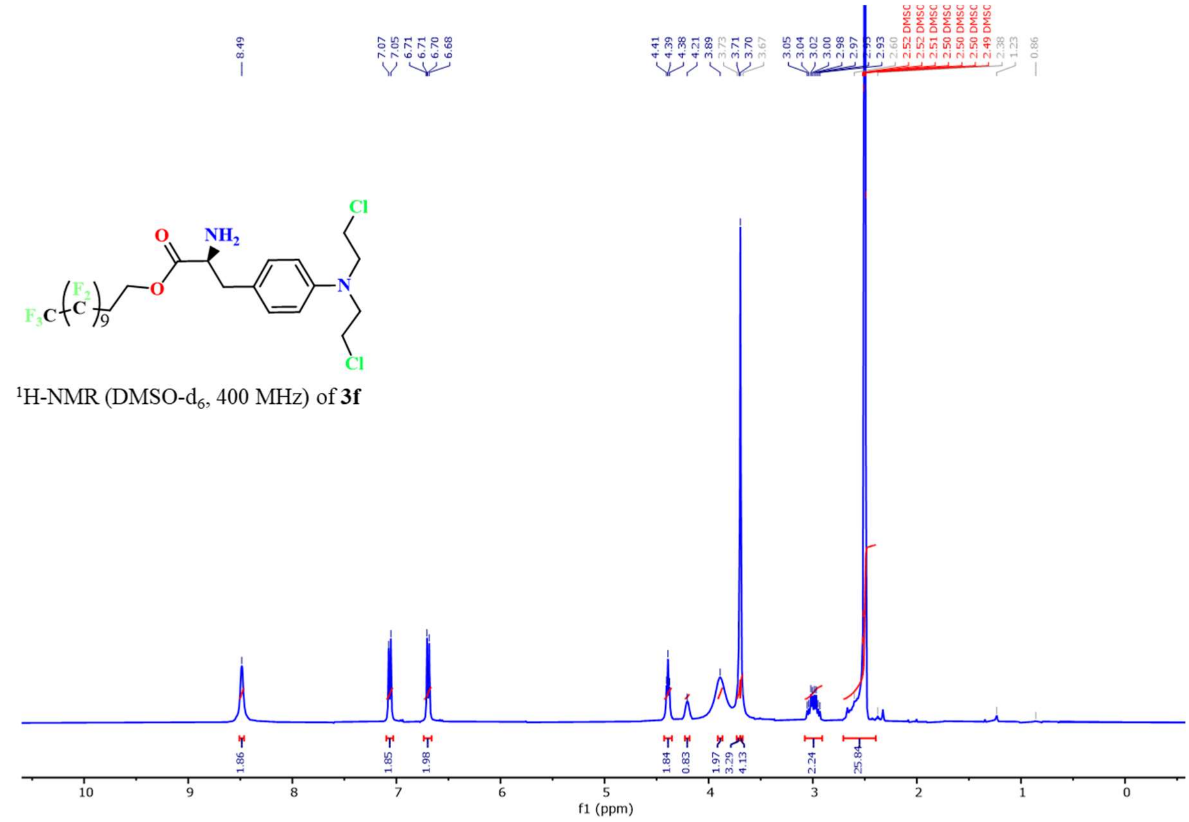


$^{1}$H-NMR (DMSO-d$_6$, 400 MHz) of **3f**

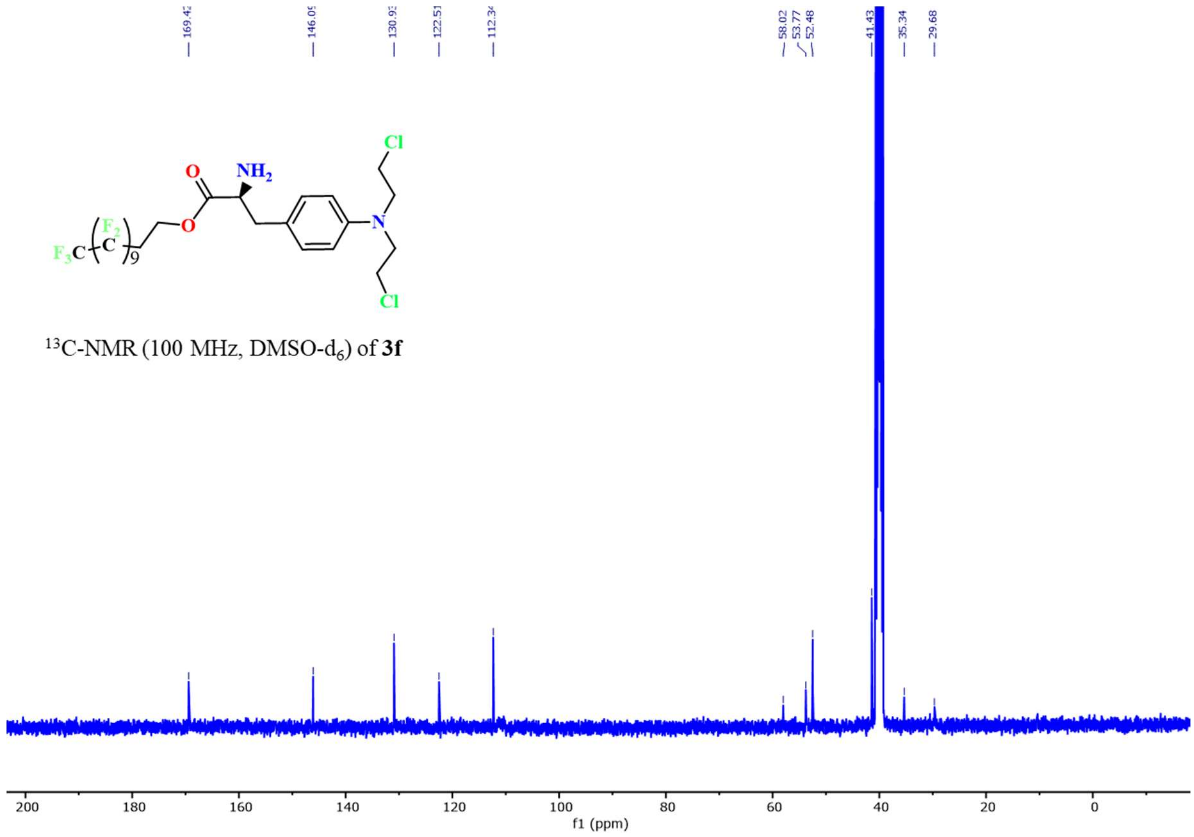


$^{13}$C-NMR (100 MHz, DMSO-$d_6$) of **3f**

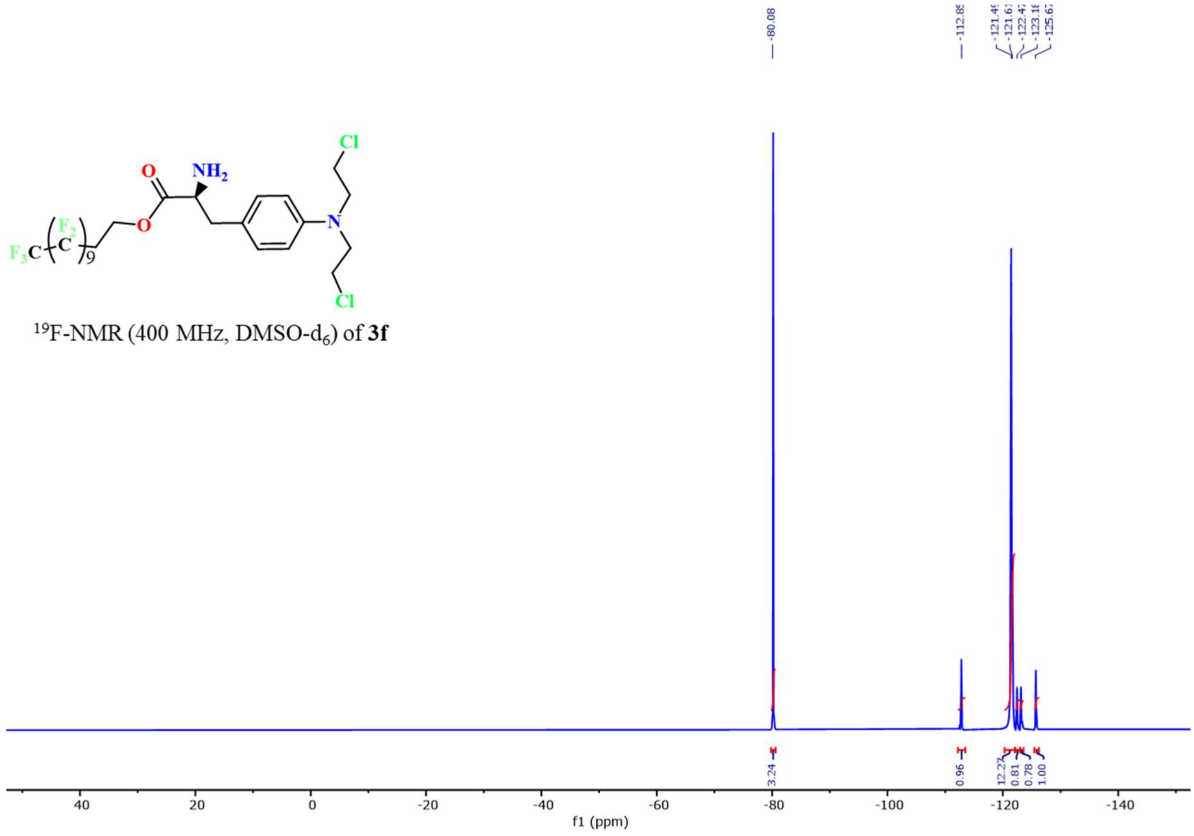


$^{19}$F-NMR (400 MHz, DMSO-$d_6$) of **3f**

## VII. Supplementary references


[1] A. Morris, G. Atassi, N. Guilbsaud, AA Cordi, *Eur. J. Med. Chem.* **1997**, *32*, 343–349.

[2] *CrysAlis$^{Pro}$*, Rigaku Oxford Diffraction, **2018**.

[3] Sheldrick, G. M. ShelXT-Integrated Space-Group and Crystal-Structure Determination. *Acta Crystallogr. Sect. A,* **2015**, *71*, 3–8.

[4] Sheldrick, G. M. Crystal Structure Refinement with ShelXL. *Acta Crystallogr. Sect. C,* **2015**, *71*, 3–8.
